# Comparative Proteomic Analysis Unveils Critical Pathways Underlying the Role of Nitrogen Fertilizer Treatment in American Elderberry

**DOI:** 10.3390/proteomes7010010

**Published:** 2019-03-20

**Authors:** Bo Yang, Andrew L. Thomas, C. Michael Greenlief

**Affiliations:** 1 Department of Chemistry, University of Missouri, Columbia, MO 65211, USA; bykm5@mail.missouri.edu; 2 Division of Plant Sciences, Southwest Research Center, University of Missouri, Mt. Vernon, MO 65712, USA; thomasal@missouri.edu

**Keywords:** elderberry, comparative proteomics, nitrogen response, *Sambucus*

## Abstract

American elderberry (*Sambucus nigra* subsp. *canadensis*) is a rapidly growing specialty crop in Missouri and eastern North America. Nitrogen (N) is a major nutrient involved in plant growth and development. However, proteome changes for different genotypes of elder in response to varying levels of N-treatment remain undefined. To reveal plant responses to N, comparative proteomic analyses were performed to determine consistent changes in three genotypes of elderberry leaves (Adams II, Bob Gordon and Wyldewood) grown under different N-fertilizer treatments. 165 proteins separated by two dimensional gel electrophoresis showed significant differences in abundance (*p* < 0.05 and greater than 2-fold). Principal component analysis of the abundance profiles of these proteins revealed Bob Gordon as a distinct genotype. The 165 proteins were identified by mass spectrometry and showed similar functional distributions in these genotypes underlying the N-treatment. Among the proteins identified, 23 are mainly involved in photosynthesis, protein metabolism and redox homeostasis. Their abundance profiles were not altered upon exposure to N or genotype. These results provide novel insights into plant responses to fertilizer treatment at the proteome level and could lead to a better understanding of molecular mechanisms of elderberry growth.

## 1. Introduction

American elderberry (*Sambucus nigra* subsp. *canadensis*) is a specialty crop grown in many parts of North America. Overall elderberry cultivation is expanding in the U.S. with the potential for further growth [1]. The cultivation of elder plants requires the use of fertilizers; most commonly nitrogen (N)–based. Therefore, improving and understanding the role of N in elder production is needed [2]. Fertilizers typically contain N in the form of nitrate and ammonium compounds, which rapidly dissolve in water, resulting in loss to the soil, and low nitrogen use efficiency (NUE) [3]. A better understanding of the molecular interactions and mechanisms that underlie elder response to different N-treatments may aid in more efficient utilization of N-fertilizer as well as furthering the understanding of elderberry growth. 

Several studies have examined gene expression changes in plants in response to N treatment. Bi and co-workers found a set of N-responsive genes in three genotypes of maize under N-limiting conditions by high throughput RNA sequencing [4]. Transcriptome analyses have identified differentially expressed genes under N-deprivation conditions and yielded a complex pathway in the model plant *Arabidopsis thaliana* [5]. However, the exact function of these genes remains unknown. Proteomic approaches can aid in the understanding of changes and functions of the plant proteome to N [6,7,8,9]. Proteomics can complement many functional genomics approaches [10] and provide useful information about post translational modifications (PTM) with annotation of the corresponding gene [11]. While several studies have found a proportional relationship between transcript levels and the abundance of proteins [12,13,14], it is still difficult to predict the relationship between cellular protein concentrations and the abundance of corresponding mRNAs [15]. Part of the difficulty is likely due to PTMs and the effects of micro RNAs on expression of proteins [16]. A comparative proteomics approach could provide a better assessment of differentially abundant proteins and metabolic processes.

Here, we present a comparative proteomics investigation of the impact of N-fertilizer treatment on American elderberry (*Sambucus nigra* subsp. *canadensis*). Three genotypes are used in the investigation; including an heirloom genotype (Adams II) and the more recently developed genotypes (Bob Gordon [17] and Wyldewood [18]). The selection of suitable N-fertilizer usage is important to ensure agriculture productivity and sustainability [2]. Elder leaves were chosen for the study due to their sensitivity to varying environmental conditions [19]. Two-dimensional electrophoresis (2-DE), followed by in-gel digestion, and mass spectrometry (MS) allowed us to identify 165 proteins that showed altered abundance upon exposure to different levels of N-fertilizer treatment. Protein functional studies helped to further our understanding of protein profile changes in response to treatment. These results provide novel insight into cellular changes which could lead to new N-treatment management strategies to improve NUE and plant production in the future.

## 2. Materials and Methods

Acrylamide, phenol (Tris-equilibrated), CHAPS, 3-(4-heptyl) phenyl-3-hydroxypropyl) dimethylammoniopropanesulfonate (C7BzO), dithiothreitol (DTT), iodoacetamide (IAA), EDTA and 2-hydroxyethyl disulfide (2-HED) were obtained from Sigma-Aldrich (St. Louis, MO, USA). Ammonium persulfate, bis-acrylamide, urea (electrophoresis grade), thiourea (electrophoresis grade), ammonium acetate, glycerol, Coomassie Brilliant Blue G-250, agarose (low EEO) and 2-mercaptoethanol were obtained from Thermo Fisher Scientific (Houston, TX, USA). *N*,*N*,*N*′,*N*′-tetramethylethylenediamine (TEMED) was obtained from BioRad Laboratories, Inc. (Hercules, CA, USA). EZQ protein quantification kits were obtained from Molecular Probes (Invitrogen, Carlsbad, CA, USA). IPG strips and IPG buffer 3–10 were obtained from GE Healthcare (Piscataway, NJ, USA). Modified porcine trypsin was acquired from Promega (Madison, WI, USA).

Study materials (leaves) were harvested from an active research trial in southwest Missouri that incorporated three elderberry genotypes and four N-fertilization treatments (applied 9 May 2014 and the two previous springs), as previously described [20]. Fresh, fully mature elder leaves from the middle portions of branches were randomly collected from 36 of the 48 plots in August 2014, promptly refrigerated, and transported under refrigeration to the laboratory for analysis. About 1000 cm^3^ of leaves were collected from each plot. The leaves were collected after fruit had been harvested. The middle portions of the compound elder leaves were then washed, ground under liquid nitrogen, and 100 mg of each replicate was aliquoted into 2 mL microcentrifuge tubes and stored at −80 °C prior to protein extraction.

The proteins were extracted according to a method described by Hurkman and Tanaka with some modifications [21]. Briefly, proteins were isolated by phenol and precipitated overnight by five volumes of pre-chilled 0.1 M ammonium acetate in 100% methanol. Protein concentration was determined by EZQ quantitation kits (Life Technologies, Grand Island, NY, USA) using the manufacturer’s instructions. Proteins were separated by 2-DE as previously described [22]. All 2-DE stained gels were scanned using a UMAX PowerLook 2100 XL scanner (UMAX Systems GmbH, Willich, Germany). At least triplicate gels were performed for each treatment, and a total of 36 gel images were analyzed by ImageMaster 2D Platinum software version 7.0 (GE Healthcare). To compensate for gel-to-gel variation, the volume of each spot was normalized as a relative volume as previously described [23].

Three replicates were performed for each genotype at each N-fertilizer treatment. One-way analysis of variance (ANOVA), part of ImageMaster 2D Platinum (Version 7.0; GE Healthcare), was initially performed for each group to identify statistical significance by using the relative volume (vol %). A significance level of 95% was chosen. To evaluate the quality of spots, several parameters were set manually. The 2-fold difference calculated as the ratio of the average spot volume of each treatment to that of the control was used; spots were observed in at least 80% of spot maps and repeated on triplicates. Once the spots were considered to be statistically significant, the average values of these spots were analyzed by Tukey’s honest significant difference test, and then filtered by two-way ANOVA by genotype, two-way ANOVA by N-treatment, and/or two-way ANOVA interaction (*p* < 0.05) using the SAS software package (Version 9.4, SAS Institute, Cary, NC, USA). Only spots of interest were selected for further MALDI-TOF/TOF or LC-MS/MS analysis. Additionally, principal component analysis (PCA) was performed using the SAS software.

Six pooled gels were generated by pooling equal amounts (200 µg) of three biological replicates onto one gel following the same steps as described above. Each protein spot of interest was excised from the pooled gel, trypsin digested and identified by MALDI-TOF/TOF (4700 Proteomics Analyzer, Applied Biosystems, Foster City, CA, USA) in the positive ion reflector mode as previously described [22]. For peptide and protein identification of MALDI-TOF/TOF data, the resulting peptide peak lists were submitted to the MASCOT database search engine against the National Center for Biotechnology Information non-redundant (NCBInr) database. A homology search was performed due to the limited sequences of *Sambucus nigra* genome (164 sequences as of May 2018). The following parameters were selected: *Viridiplantae* (Green Plant) as taxonomy, trypsin as digesting enzyme, one missed cleavage, fixed modification of carbamidomethyl (Cys), variable modification of oxidation (Met), precursor ion mass error tolerance of 100 ppm, MS/MS fragment ion mass error tolerance of 0.1 Da, peptide charge of 1+, monoisotopic and MALDI-TOF/TOF as instrument. Confident protein identifications were defined as: (1) the highest protein score on the database searching report, (2) a minimum of two matched peptides, (3) less than 15% deviation between theoretical and experimental Mr and pI values (gel-based). An in-house BlastP search at NCBI was performed to verify all matches and update annotations and identification of all hypothetical or unknown proteins (sequence similarity > 80%).

In cases where the MASCOT search did not reveal confident identification, we performed LC-MS/MS experiments as previously described [24]. For peptide and protein identification by LC-MS/MS, raw files were analyzed and quantified using the SEQUEST algorithm implemented with a Scorcerer2 integrated Data Appliance (SageN Research, Milpitas, CA, USA) against a local copy of the *Viridiplantae* database downloaded in FASTA format via file transfer protocol from NCBInr (released in November, 2013; 2,355,794 proteins). Homology searches included full trypsin specificity (KR/P), two missed cleavage sites, peptide mass tolerance of 50 ppm, fragment ion mass tolerance of 1 Da, carbamidomethylation of Cys, as a static modification and Met oxidation as variable modification. Search results were verified by Scaffold viewer V4.0.5 (Proteome Software Inc., Portland, OR, USA). For each identification, a minimum of two matched peptides; peptide threshold (*p* < 0.05) and protein threshold (*p* < 0.001); cross-correlation factor (Xcorr) 2.0, 3.0 and 3.5 for the charge states and +2, +3 and +4 respectively, and minimum Delta CN (Delta correlation) of 0.1. Results were then filtered using the Protein- and PeptideProphet [25,26] implemented in the Scaffold software to achieve a peptide and protein global false discovery rate of <5 and 0.1%, respectively. An extra BlastP search was also performed to update the best matches and align all hypothetical or unknown proteins (sequence similarity > 80%).

Hierarchical clustering was constructed using the software PermutMatrix [27]. The average abundance of identified proteins, which were classified in the same orthologous group and displayed similar trends of abundance profiles across all genotypes, were taken and the fold change calculated between each group and control, followed by log2 transformation for heat map representation. The dissimilarities were calculated based on Euclidean distances and hierarchical clustering was carried out according to Ward’s method. The trees were generated using the multiple-fragment heuristic algorithm (MF) as a seriation rule.

The function of the identified proteins was sought by transferring original sequences to the *Arabidopsis* genome and orthologous genes using the Mercator web-based pipeline (http://mapman.gabipd.org/app/mercator), which divides protein into 35 hierarchical, nonredundant functional classes using MapMan bin codes [28]. The cellular locations of identified proteins were determined by searching the best matched *Arabidopsis* orthologous proteins in the SUBA database (Version 3) [29].

## 3. Results

### 3.1. Comparative Proteome Analyses and Differentially Abundant Proteins in Elderberry Leaves

To investigate the effect of N-fertilizer treatment upon protein profiles of elder leaves, three different applications were applied to the plants (56, 112 and 169 kg N/ha). Control plants had no N-fertilizer applied. The three N-fertilizer applications can be thought of as a low, medium, and high concentration. 2-D gel electrophoretic separation of the proteins extracted from leaves of three genotypes (Adams II, Bob Gordon and Wyldewood) of American elderberry was performed. A combination of 12 treatments of genotype × N-concentration as a factorial design was developed, along with three replicates derived from separate leaf extractions giving a total of 36 images. The correlation coefficients among the biological triplicates were shown to vary from 0.9863 to 0.9993 (Figure S1). Replicate gel images were then digitally integrated to build a “master gel” for each treatment condition. Three different groups were established to identify differentially abundant proteins; Group 1: 0 (or control) vs 56 kg N/ha, Group 2: 0 vs 112 kg N/ha, and Group 3: 0 vs 169 kg N/ha. On average, 300 protein spots were initially detected on each 2-DE gel with some distinctive changes within genotypes or N-fertilizer conditions. A one-way ANOVA analysis was applied to all matched spots in each group (*p* < 0.05). Once the protein spots were determined to be differentially abundant, the spots were selected using the following stringent criteria; at least 2-fold change, correlation of all spot maps, and appearance on all replicates. Of the 3564 spots initially detected among the master gels, only 165 “high class” protein spots fit these criteria (Table 1). 

For the 165 protein spots which met the above criteria, a *post hoc* Tukey test was conducted to determine statistical differences in abundance changes between each treatment and control. A two-way ANOVA analysis was performed on the 165 protein spots to assess any significant differences due to either genotype or N-treatment on protein abundance (Table S1). The results of the two-way ANOVA analysis revealed that genotype had a significant effect on the expression of 70 protein spots, whereas N-treatment had no effect. For 10 of the protein spots N-treatment resulted in significant changes in abundance, but genotype had no effect on these spots. Both genotype and N-treatment had a significant effect on the expression level of 30 protein spots. Genotype and N-treatment had an interaction effect on the abundance level of 55 protein spots. Based on the analysis, we were able to identify significant protein abundance level changes and the conditions resulting in abundance change.

The representative 2-DE gel maps are shown in Figure S2. Pick locations of spots that have significant volume changes based on the variance of group means are labeled. The proteins that have same location (Mr and pI) throughout three groups are considered to be the same proteins which reduced the number of spots for MS analysis to 101.

### 3.2. Protein Identification

15 proteins were successfully identified by MALDI-TOF/TOF, whereas the remaining 86 proteins were determined by LC-MS/MS (Tables S2 and S3). Notably, 39 proteins were interpreted either as unknown and hypothetical proteins, or proteins without specific function in the database. This indicates that the comprehensive function of many N-responsive proteins from *Sambucus nigra* subsp. *canadensis* have yet to be elucidated. To gain further information about these unknown proteins, we performed a BLASTP search of their homologues using original sequences as queries. The corresponding homologues with the highest homology are shown in Tables S4–S6. All 39 proteins shared more than 80% positive identity with homologues at the amino acid level, showing that they might have similar function. The relative expression level for the 101 identified proteins are also listed in Tables S4–S6. In particular, 63 proteins displayed different experimental Mr and/or pI and, confirmed by the identification of at least two unique peptides, were identified as protein isoforms. For example, isoforms were observed for the 20 kDa chaperonin family protein (CPN21; spots 49, 56, 61 and 90), the ATP synthase CF1 α subunit (ATP-SCF1S; spots 13, 14 and 15), and catalase (CAT; spots 18 and 19).

### 3.3. Hierarchical Clustering of Differentially Abundant Proteins in the Three Treatment Groups

Overall, there are 52 up-regulated spots and 73 down-regulated spots for Adams II. 42 up-regulated spots and 71 down-regulated spots are detected for Bob Gordon. 55 up-regulated spots and 70 down-regulated spots are detected for Wyldewood. The number of differentially abundant proteins for down-regulated was greater than up-regulated across the three genotypes at each N-fertilizer rate (except Adams II at 169 kg N/ha and Wyldewood at 112 kg N/ha). For Bob Gordon, there were about twice as many down-regulated differentially abundant proteins, indicating that Bob Gordon is dissimilar to Adams II or Wyldewood.

Protein abundance analysis for a given fertilizer treatment and genotype can highlight proteins which may respond to N during plant growth and development. 13 proteins showed the same trend of regulation (up or down regulation) for a given N-treatment across the three genotypes. In Group 1, ATP-dependent zinc metalloprotease FTSH 1 (FTSH1; spot 11), CA2 (spot 62) and actin 11 (ACT11; spot 86) were down-regulated. In Group 2, eukaryotic initiation factor 4A-8 (EIF4A8; spot 22) and citrate synthase family protein (CS; spot 23) were up-regulated, while heat shock protein 70 (HSP70; spot 5), 30S ribosomal protein S1 (RPS1; spot 21), probable plastid-lipid-associated protein 6 (PAP6; spot 53) and AMT (spot 87) were down-regulated. In Group 3, 20S proteasome subunit PBA1 (PBA1; spot 98) was up-regulated. In contrast, V-type proton ATPase catalytic subunit A (VHA-A; spot 12), sedoheptulose-1,7-bisphosphatase (SHB; spot 97) and RPI (spot 99) were down-regulated (Table S1). 

**Figure 1:**
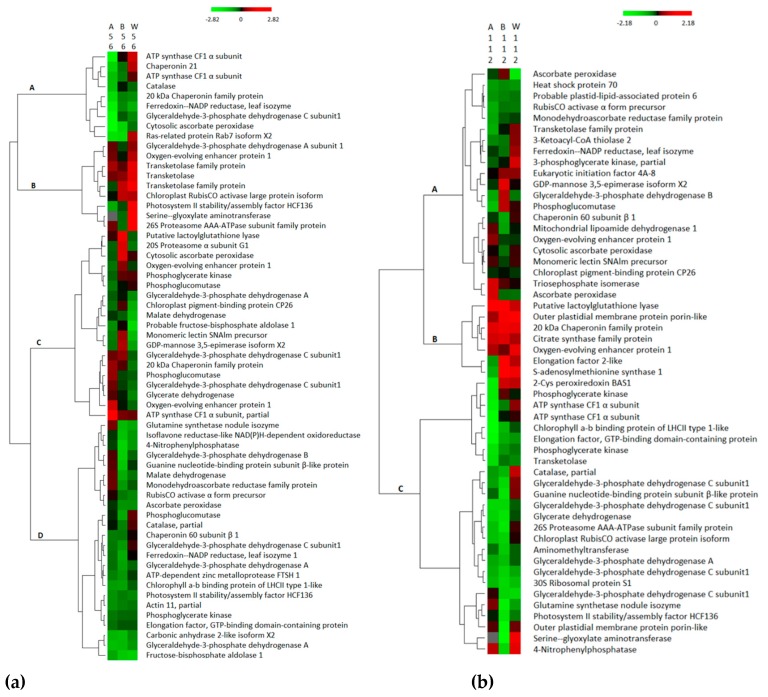

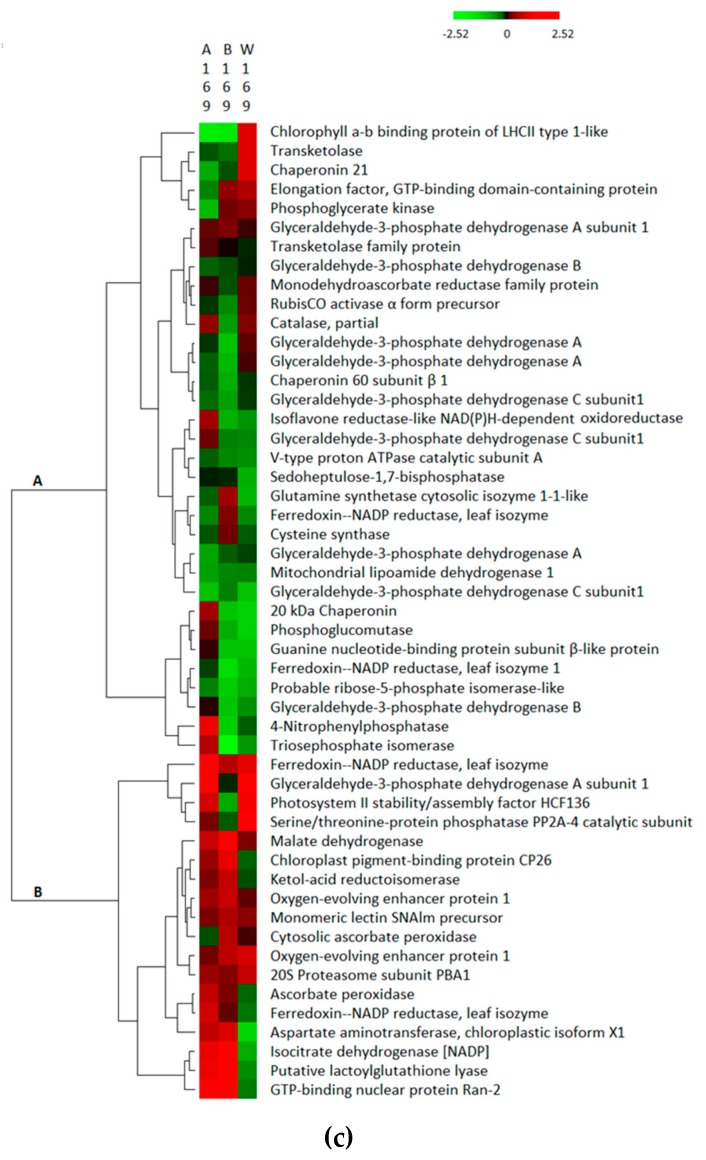
(**a**–**c**) Hierarchical clustering of 165 differentially abundant proteins (*p* < 0.05) for each N fertilizer concentration (number following A (Adams II), B (Bob Gordon), W (Wyldewood)). The abundance of each protein was transformed using log2. Every colored box represents a protein that is up-regulated (red) or down-regulated (green) in a certain treatment. Gray color means + ∞. Hierarchical clusters (A, B, C, and D) were established as described in the text.

Also, a hierarchical clustering of 165 differentially abundant proteins was performed with the aim of evaluating similarity of protein regulation in leaf organs across the different genotypes in each N-treatment group as shown in Figure 1. In Figure 1a, the low N-treatment, 61 proteins were distributed in four main clusters. Cluster A included nine proteins abundances that were mainly down-regulated in the three genotypes and all Adams II protein abundances in this cluster are down-regulated. Cluster B comprised nine protein groups with the largest abundance values of the genotypes. In Cluster D of Figure 1a, most of the protein abundances are down-regulated and all the Bob Gordon proteins abundances in this cluster are down-regulated. In Figure 1b, the medium N-treatment, 53 proteins fell into three clusters. Cluster B included seven proteins and represents the largest expression level. Cluster C proteins were mainly down-regulated in the three genotypes, but the downregulation in Bob Gordon was the strongest. In Figure 1c, the high N-treatment, two main clusters were recognized based on 51 protein expression profiles. Cluster A was mainly down-regulated in three genotypes, while cluster B was up-regulated, where the Adams II and Bob Gordon expressions were larger than that of Wyldewood.

### 3.4. Principle Component Analysis (PCA) of Each Group

Global correlation of each group is visualized by plotting the two largest sources of variance, PCA1 and PCA2, which orthogonally divide the spots maps among expression data (Figure S3). In general, the first two components of Group 1 (3 genotypes, low N-treatment), Group 2 (3 genotypes, medium N-treatment), and Group 3 (3 genotypes, high N-treatment) contained 93.64%, 95.04% and 94.32% of the total data variance, respectively. The largest variance (PCA 1) represents technical and biological variation. The second largest variance (PCA 2) represents applied treatment variation. PCA reveals that the largest variation is not due to genotype or N-fertilizer treatment. PCA of the 165 differentially abundant proteins in the three groups demonstrated that, with and without N fertilizer treatment, Bob Gordon has a protein expression profile distinct from that of Adams II and Wyldewood. This result suggests that Bob Gordon is a distinct phenotype. The correlation of Adams II and Wyldewood demonstrated a great similarity among different N fertilizer treatments. However, PCA could not distinguish between control and each N-treatment of Adams II, Bob Gordon or Wyldewood. The same trends are shown across the three groups in the PCA analysis, demonstrating a satisfactory reproducibility of the experimental processes. 

### 3.5. Functional Classification and Cellular Localization of Differentially Abundant Proteins 

**Figure 2:**
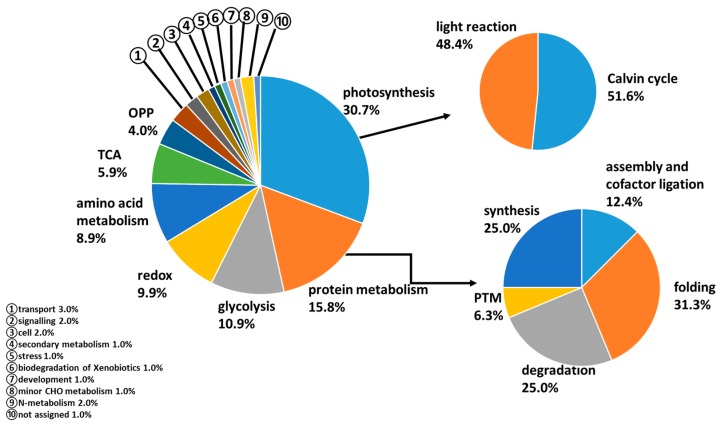
The functional category distribution of the 101 identified proteins. Proteomes identified in the leaf were matched to their *Arabidopsis* orthologues and classified into 16 major functional groups using the Mercator software (gabipd.org/biotools/mercator/). Proteins involved in photosynthesis and protein metabolism were further sub-classified as indicated by arrows. OPP: oxidative pentose phosphate; TCA: tricarboxylic acid; PTM: post translational modification.

The 101 identified proteins were classified into functional groups including the largest group photosynthesis (30.7%), followed by protein metabolism (15.8%) and glycolysis (10.9%), redox homeostasis (8.9%), amino acid metabolism (8.9%), tricarboxylic acid (TCA) cycle (5.9%), oxidative pentose phosphate (OPP) (4.0%) pathway and miscellaneous (14.9%) (Figure 2). Proteins involved in photosynthesis were classified into two categories, including Calvin cycle and light reaction. Proteins involved in protein metabolism included those involved in protein assembly and cofactor ligation, folding, synthesis, degradation and PTM. The 101 identified proteins were assigned to six major cellular locations (Tables S4–S6). Most proteins were found in the chloroplast and cytosol. The remaining proteins were discovered in the mitochondrion, peroxisome, Golgi apparatus and/or nucleus. Both functional classification and cellular localization results show that a large number of chloroplast proteins are involved in photosynthesis, which were likely greatly affected by N-treatment. 

Using the same approach, similar distributions were observed for up- or down-regulated differentially abundant proteins for each genotype and N-treatments (Figure S4). The functional classification of N characteristic-proteins exhibited expression profiles associated with key roles in photosynthesis, protein metabolism, glycolysis and redox homeostasis. The functional classes are also included for each identified protein in Table S4.

### 3.6. Expression Profiles of Unique Proteins in Response to N Across Three Genotypes

A total of 23 unique proteins representing 58 isoforms appeared in all three groups. This profile was not altered upon exposure to N or genotype. Table 2 summarized the identified proteins, spot number, and functional class. Not surprisingly, photosynthesis and protein metabolism comprise the majority of functional classes for the leaves.

**Figure 3:**
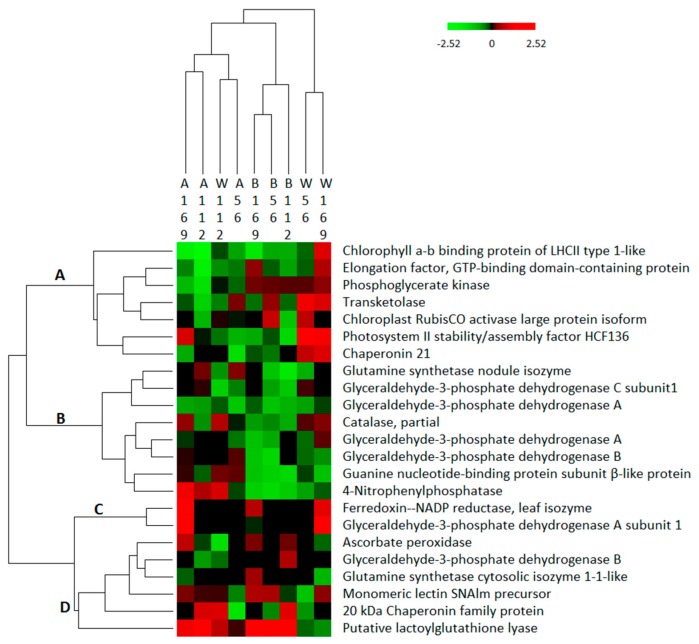
Hierarchical clustering of orthologous N characteristic-protein groups consistently enriched in three N groups compared to control (*p* < 0.05) in different genotypes. The color gradient range of the heat map indicates the proportional up-regulation (red) and down-regulation (green) of protein abundance (fold difference, log2-transformed). Hierarchical clusters (A, B, C, and D) were established as described in the text.

Among these, 18 unique proteins appeared as 23 isoforms which agrees well with the two-way ANOVA results (genotype × N interaction, *p* < 0.05). In some instances, the same proteins (or isoforms) were assigned to different effects across three groups, suggesting the concentration may change. Thus these consistently shown proteins are represented in important pathways and functional classes. We performed hierarchical clustering to compare trends of proteins enrichment in response to all N treatments among the different genotypes. These protein groups fell into four main clusters (Figure 3). Four photosynthesis related proteins (LHCII type 1, PGK, RCA and HCF136) were distributed in cluster A along with other proteins such as EF, TK and CPN21. These proteins were particularly up-regulated in Wyldewood at 56 and 169 kg N/ha. Cluster B comprised most glyceraldehyde-3-phosphate dehydrogenase proteins, CAT, 4-NPPase, one isoform of GS and GNBP, which were mainly down-regulated across genotypes and N treatments. Cluster C contained LFNR and GAPA proteins which were mainly up-regulated at 169 kg N/ha regardless of genotype analyzed. Finally, in cluster D, APX, GAPB, SNAlm, one isoform of GS, one isoform of CPN21 and LGL were grouped. Also, expression profiles from all genotypes and N treatments were clustered and are represented at the top of the columns in Figure 3. Bob Gordon under different N-treatments were clustered together, indicating similarity of expression of Bob Gordon among N fertilizers. In summary, the global abundance of proteins related to OPP pathway, detoxification, carbohydrate recognition, protein metabolism (elongation, folding and assembly) and redox homeostasis were upregulated at 169 kg N/ha compared to control, while proteins associated with development, glycolysis, photosynthesis, minor CHO metabolism and ammonium assimilation were down-regulated.

**Figure 4:**
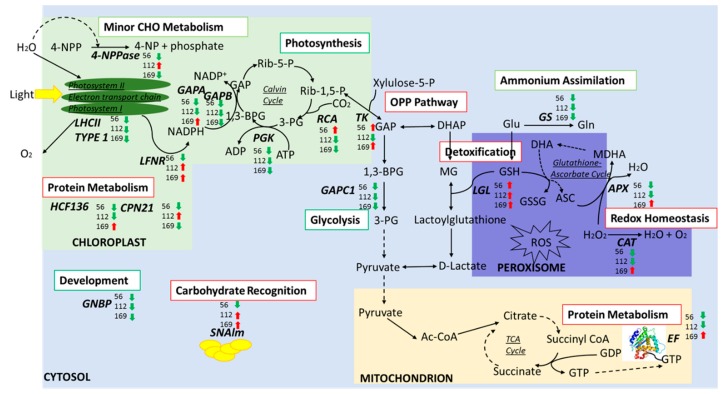
Schematic representation of key pathways that regulate N fertility. These differentially abundant proteins (interaction *p* < 0.05) across different genotypes and N treatments were also classified in functional categories using MapMan bin codes (http://mapman.gabipd.org). The cellular locations of each protein were determined by the SUBA database (Version 3). Arrows indicate up- and down-regulation of proteins within each set of isoforms compared to control group at a given N condition. Red squares in the functional category indicate an overall regulation of the category at 169 kg N/ha compared to control (0 kg N/ha) in different genotypes, while green squares means down regulation.

Figure 4 summarizes the major pathways identified and the protein abundance changes with treatment. Our results showed that 13 proteins representing 21 isoforms had similar up- or down-regulated change patterns in response to all N-treatment groups. For example, the protein GLO (spot 36) was up-regulated in both Adams II and Bob Gordon. In contrast, CPN 60 (spot 10) and LHCII type 1 (spot 46) were down-regulated in both Adams II and Bob Gordon. For each genotype, three isoforms of PGK (spots 27, 30 and 31) and EF (spot 32) were down-regulated in Adams II. Two isoforms of CAT (spots 18 and 19), MDAR (spot 29), two isoforms of HCF136 (spots 37 and 40), 4-NPPase (spot 43) and GNBP (spot 72) were down-regulated in Bob Gordon. Three isoforms of GAPB (spots 26, 28 and 75), two isoforms of GS (spots 33 and 34) and two isoforms of CP26 (spots 48 and 52) were down-regulated in Wyldewood (Table S1).

On the other hand, nine proteins appeared as 36 isoforms, and consistently exhibited opposite expression patterns within each set of isoforms across all genotype and N groups. These proteins included three isoforms of TK (spots 2, 3 and 4), four isoforms of PGM (spots 6, 7, 8 and 9), two isoforms of RCA (spots 20 and 35), four isoforms of OEE1 (spots 41, 44, 57 and 58), four isoforms of APX (spots 51, 54, 55 and 59), five isoforms of LFNR (spots 64, 69, 70, 91 and 92), five isoforms of GAPA (spots 65, 66, 73, 76 and 101), five isoforms of GAPC1 (spots 74, 77, 78, 81 and 89) and four isoforms of CPN21 (spots 49, 56, 61 and 90). In addition, one protein, SNAlm (spot 42), which was identified in European elder (*Sambucus nigra* subsp. *nigra*) [30], showed opposite expression patterns in different N groups regardless of genotype analyzed. Three isoforms of GAPB (spots 26, 28 and 75), two isoforms of GS (spots 33 and 34) and two isoforms of CP26 (spots 48 and 52) were also consistently observed to have opposite abundance levels for Adams II and Bob Gordon. Two isoforms of CAT (spots 18 and 19) and two isoforms of HCF136 (spots 37 and 40) were found for Adams II and Wyldewood. Three isoforms of PGK (spots 27, 30 and 31) were represented for Bob Gordon and Wyldewood (Table S1).

## 4. Discussion

It is essential to develop a more comprehensive understanding of how different plants respond to different levels of N-fertilizer in the field. In this study, we exploited a comparative proteomics approach to identify specificity and dynamic changes in leaf proteomes of American elderberry affected by N-treatment (Table 1). Protein functional classification with their *Arabidopsis* orthologues was adopted to enable us to evaluate the expression level of the same proteins in different genotypes. Understanding how American elderberry responds to different levels of fertilizer not only permits us to better manage its utilization, but also to understand the growth and development of elder, possibly improving the yields of different genotypes with optimized choice of N-fertilizer usage.

2-DE coupled with MALDI-TOF/TOF and LC-MS/MS and advanced statistical methods were used in this study as an efficient proteomics approach, allowing us to observe spatial difference in protein expression. 165 protein spots (approximately 5% of all detectable protein spots) showed significant changes in response to N-treatment compared to control. PCA analysis revealed that the protein expression profile of Bob Gordon differs from that of the other two genotypes, indicating that Bob Gordon is a distinct phenotype and sensitive to N-treatment. This is consistent with the observation that Bob Gordon has the highest yield among the three genotypes under same growing conditions [31]. Our study suggests that Bob Gordon may possess widespread usefulness as a commercial elder cultivar and is worth further study.

According to the results of the two-way ANOVA analysis (Table S1), some of the differentially abundant proteins were assigned to N-treatment as the main effect. Phenotypically, three genotypes behave differently when cultivated side-by-side, suggesting that the different inherent genetic backgrounds play a role [31]. However, some differentially abundant proteins still had the same pattern of regulation across different genotypes with the same N-treatment indicating common pathways among genotypes.

Proteins tied to photosynthesis were differentially abundant in the study. These proteins have been previously highlighted in different species upon varied N-treatment [9,32,33,34,35,36,37]. Most of these photosynthetic proteins showed a lower expression level with increasing N-fertilizer concentrations, regardless of genotype (Figure 4). Our findings are consistent with earlier work which showed that the Rubisco activation state decreased at high N-concentrations and that Rubisco may serve as a storage protein in apple leaves [38]. A previous study on maize found lower intercellular CO_2_ concentrations in response to high N supply also consistent with our results [39]. A recent genetic study showed that the OsPIPs gene may be responsible for mediating insufficient CO_2_ assimilation [40]. We speculate one of the outcomes of decreased level of RCA in elderberry leaves with a high N-treatment could lower the CO_2_ concentration in the chloroplasts, that may be an indication of over-fertilization.

In addition to photosynthetic proteins, we could observe a considerable distribution of proteins involved in protein metabolism (synthesis, PTM, degradation, folding, assembly and cofactor ligation). Several components of the protein synthesis machinery (translation elongation and initiation factors and ribosomal proteins) except EF, were only expressed at a treatment of 112 kg N/ha. Elongation factors are known for regulating the translocation step in polypeptide chain elongation. The VAJ/GFA1/CLO gene encodes a translational elongation factor-2 family protein, which controls directional floral organ growth in *Arabidopsis thaliana* possibly through pre-mRNA splicing [41]. Similarly, eukaryotic initiation factors (EIF) are phosphorylated to modulate the rate of mRNA binding [42]. In tobacco, the expression of elongation factor EF-1 alpha increased in meristems, rapidly growing tissues, and developing gametophytes [43]. In our study, EF2 and EIF4A8 were found to be enriched in Bob Gordon and Wyldewood, suggesting enhanced protein synthesis to support the development and growth of plant. In *Arabidopsis thaliana*, mutations in genes encoding ribosomal proteins affect leaf developmental processes [44]. We suspect deficiency of the EF protein may play a role in perturbing the development of leaf under high N-treatment. Chaperone proteins (CPN21 and 60) are essential for facilitating protein folding, unfolding and preventing protein aggregation [45]. Others suggest it may regulate increased oxidative stress [46]. Consequently, the increased amounts of CPN21 may help to lower oxidative stress at a treatment of 112 kg N/ha. A comparative genetics study of mutants in *Arabidopsis thaliana* has implicated an inverse relationship between 26S proteasome (26SP) and 20S proteasome (20SP) abundance, with defectiveness 26SP level invariably causing the elevated abundances of free 20SP lead to increased tolerance to oxidative stress [47]. Our results agree well with the established correlation between 26SP and 20SP. We constantly observed enhanced expression of 20SP concurrently with the down-regulation of 26SP at different N levels. The enriched abundance of serine/threonine-protein phosphatase PP2A-4 catalytic subunit (PP2A-4) was presented for Adams II and Wyldewood at a treatment of 169 kg N/ha. Many functional roles of PP2A in stress signal pathways were discussed under biotic and abiotic conditions [48]. Its role in N stress is yet to be determined.

Proteins involved in amino acid metabolism, such as ketol-acid reductoisomerase (KARI), s-adenosylmethionine synthase 1, aspartate aminotransferase (AST), and GDH, were differently abundant in the three N groups. Amino acid biosynthesis and degradation tightly correlate with N-availability [49]. These proteins are involved in the biosynthesis of the branched-chain amino acids [50], glycine, serine, aspartate [51] and cysteine, respectively. KARI was also identified in maize ear with 2-DE gels under different N-conditions, which correlates with ear growth and grain yield [52]. In the case of AST, it is involved the signal transduction pathway to regulate asparagine to glutamine ratio in cobs and that ratio shows the plant N status [53]. Additionally, the ascending amount of AST likely coordinates with increasing maize productivity [54]. Taken together, the increased amount of these two proteins in Bob Gordon at a treatment of 169 kg N/ha may help explain the higher yield that is often observed under field conditions [31]. GDH and AMT, which are involved in amino acid degradation were found down-regulated for all three genotypes at a treatment of 112 kg N/ha.

Several proteins involved in redox homeostasis were found to exhibit altered expressions, including CAT, GDP-mannose 3,5-epimerase isoform X2 (GME), MDAR, 2-cys peroxiredoxin BAS1 (BAS1) and APX. Redox changes show well-organized interactions to prevent tissue damage and death under environmental disturbance [55]. Ascorbate-glutathione cycle plays a key role in H_2_O_2_ metabolism in plant cells [56]. H_2_O_2_ is reduced to water by APX using ascorbate as the electron donor and the reducing agent ascorbate is regenerated by MDAR and dehydroascorbate reductase using reduced glutathione [57]. The increased levels of APX and MDAR for Adams II were found at a treatment of 169 kg N/ha. Similarly, GME is involved in the regulation of ascorbate biosynthesis [58] and was found enriched for Bob Gordon at a treatment of 112 kg N/ha. APX was increased in the same genotype. Both BAS1 and CAT participate in the reduction and degradation of H_2_O_2_ into water [59,60]. There was an enhanced abundance of BAS1 and CAT in Wyldewood at a treatment of 112 kg N/ha. In summary, these results suggest a possible enhanced defense in the plant against oxidative stress at higher N-conditions.

One notable feature of this study was the identification of multiple protein isoforms associated with N-treatment [61]. About 62% of 101 non-redundant proteins were recognized as isoforms. These isoforms show different Mr and/or pI changes on the 2-DE gels in response to N-treatment. There are several reasons for this phenomena, including genetic variation, alternative splicing of RNA transcripts, and PTMs (ubiquitination, acetylation, glycosylation, and/or phosphorylation) [62]. Many of the protein isoforms reported in the present study would be omitted using the label free mass spectrometry method (i.e. geLC-MS), which provides overall changes in protein abundance [63]. Different protein isoforms have been found to play an important role in mediating N. In maize, three GS2 isoforms were identified in the root and the accumulation trend of phosphorylated isoform is consistent with plastidial GS activity. Moreover, nitrate and ammonium have been found to induce the accumulation of different isoforms [64]. Similarly, the physiological role of a new isoform of GS has been reported in the leaf of wheat, suggesting a complex and flexible regulation for GS isoforms underlying the wheat that is associated with N utilization and plant growth [65]. In the present study, two isoforms of GS were consistently found to have opposite expression levels for Adams II and Bob Gordon among the three N-treatments, while the same down-regulated changes were observed for Wyldewood. The modification of multiple protein isoforms may give us novel perspectives to understand plant response to N-treatment.

As described above, the comparison among different levels of N in all genotypes reveled 23 unique proteins representing 58 isoforms. Among these, 18 unique proteins illustrating 23 isoforms showed a significant genotype × N level interaction (*p* < 0.05). These conserved proteins were then classified into protein orthologous groups and summarized in the cellular critical pathways. As protein modification may play an important role in this study, the detection of PTMs by three-dimensional gel electrophoresis may warrant further investigation [66]. However, we note the stringent conditions used in our study for the identification of differential abundant proteins by 2DE should help to diminish issues with co-migration of proteins.

It is well known that under N starvation, accumulation of lipid degradation and toxic compounds in plants can induce ROS [67]. To protect the cell from oxidative damage, ROS-scavenging pathways are activated [68]. Our proteomics results revealed that a number of enzymes involved in protein metabolism, redox homeostasis and transportation (indicated as red squares in Figure 4) were up-regulated for the three genotypes at a treatment of 169 kg N/ha, suggesting that oxidative stress could be more efficiently reduced at high N-concentrations. As part of the plant defense system, detoxification of non-enzymatic components (i.e. GSH) is also activated to destroy ROS [69]. LGL, which is found up-regulated with N-treatment in our study, is involved in the GSH-based detoxification of methylglyoxal [70]. 

Changes in protein expression profiles in photosynthesis, the OPP pathway and glycolysis suggest that plants may require a certain amount of N to enable these proteins to produce energy for plant growth in its most efficient way. Similar results were found in maize leaf [37]. GS is a key enzyme in ammonium assimilation [71] and an overall decrease in abundance of GS among the three genotypes in response to increasing levels of N lower its utilization. SNAlm plays a role in carbohydrate recognition [30]. The abundance of SNAlm decreased at the lower treatment level and increased at the higher levels. Other proteins associated with minor CHO metabolism and development were down-regulated at highest fertilizer levels.

## 5. Conclusions

This is the first time that the critical response pathways in American elderberry leaves have been revealed at the proteome level as a function of fertilizer treatment. Determining the protein expression profiles of the three genotypes in response to N-treatment, suggests that Bob Gordon is a distinct genotype from the other two. The use of three commercial genotypes of elder allows us to find similar functional distributions underlying the N-treatment and to explore the conserved expression patterns of critical proteins. Moreover, identification of proteins specifically expressed in photosynthesis, protein metabolism, and redox homeostasis across three genotypes, indicates these pathways played major role in response to different N-treatments. The identification of proteins and critical pathways provides a novel perspective into the use of N-fertilizers.

## Figures and Tables

**Table 1:** Frequency of differentially abundant protein spots detected from 2-DE gel analysis among three elderberry genotypes (Adams II, Bob Gordon and Wyldewood) and three N fertilizer treatments ^a^.

N Concentration Comparison	Initial Detected Protein Spots ^b^	Differentially Abundant Protein Spots ^c^	High Class Spots ^d^	Effect ^e^				MS/MS Analyzed Spots ^f^
				G	N	G and N	G × N	
0 vs 56	306	86	61	30	4	11	16	28
0 vs 112	295	69	53	21	2	11	19	37
0 vs 169	281	68	51	19	4	8	20	36
Total	882	223	165	70	10	30	55	101

^a^ Protein spots that exhibited differential abundance were identified by MALDI TOF/TOF or LC-MS/MS. ^b^ Protein spots were detected by ImageMaster. ^c^ The number of protein spots was determined by a one-way ANOVA analysis. ^d^ Manually excluded spots that had less than a 2-fold change or absence within any of the triplicate gels. ^e^ Four effects were separated based on *p* values (G: genotype as main effect; N: N as main effect; G and N: genotype and N as main effect, but no interaction; G × N: interaction effect between genotype and N.) ^f^ Included those spots of interest that were excised, digested and analyzed by MS.

**Table 2:** Expression profiles of unique proteins in response to N across three genotypes.

Protein Name	Spot Number	Functional Class
RubisCO activase α form precursor (RCA)	20 and 35	Photosynthesis (34.8%)
Glyceraldehyde-3-phosphate dehydrogenase B (GAPB)	26, 28 and 75	
Phosphoglycerate kinase (PGK)	27, 30 and 31	
Oxygen-evolving enhancer protein 1 (OEE1)	41, 44, 57 and 58	
Chlorophyll a–b binding protein of LHCII type 1-like (LHCII type1)	46	
Chloroplast pigment-binding protein CP26 (CP26)	48 and 52	
Ferredoxin-NADP reductase, leaf isozyme (LFNR)	64, 69, 70, 91 and 92	
Glyceraldehyde-3-phosphate dehydrogenase A (GAPA)	65, 66, 73, 76 and 101	
Chaperonin 60 subunit β 1 (CPN 60)	10	Protein Metabolism (17.4%),
Elongation factor-GTP-binding domain-containing protein (EF)	32	
Photosystem II stability/assembly factor HCF136 (HCF136)	37 and 40	
20 kDa Chaperonin family protein (CPN21)	49, 56, 61 and 90	
Catalase (CAT)	18 and 19	Redox Homeostasis (13.0%)
Monodehydroascorbate reductase family protein (MDAR)	29	
Ascorbate peroxidase (APX)	51, 54, 55 and 59	
Phosphoglucomutase (PGM)	6, 7, 8 and 9	Glycolysis (8.7%)
Glyceraldehyde-3-phosphate dehydrogenase C subunit1 (GAPC1)	74, 77, 78, 81 and 89	
Transketolase family protein (TK)	2, 3 and 4	OPP pathway (4.3%)
Glutamine synthetase cytosolic isozyme 1-1-like (GS)	33 and 34	N-metabolism (4.3%)
Putative lactoylglutathione lyase (LGL)	36	Biodegradation of xenobiotics (4.3%)
4-Nitrophenylphosphatase (4-NPPase)	43	Minor CHO metabolism (4.3%)
Guanine nucleotide-binding protein subunit β-like protein (GNBP)	72	Development (4.3%)
Monomeric lectin SNAlm precursor (SNAlm)	42	Not assigned (4.3%)

## Supplementary Materials

The following are available online at https://www.mdpi.com/2227-7382/7/1/10/s1, Figure S1: Scatter graph of relative spots volumes detected on 2-DE gels, Figure S2: Representative 2-DE maps of differentially expressed proteins, Figure S3: PCA analysis of differentially expressed proteins, Figure S4: Mercator protein functional categorization of each genotype, Table S1: Statistical analysis of differentially expressed proteins, Table S2: Protein report for MALDI-TOF/TOF, Table S3: Protein report for LC-MS/MS (separate Excel file), Table S4: Identification of altered proteins after 56 kg N/ha treatment, Table S5: Identification of altered proteins after 112 kg N/ha treatment, Table S6: Identification of altered proteins after 169 kg N/ha treatment.

## References

[1] Cernusca M.M., Gold M.A., Godsey L.D. (2012). Using the Porter model to analyze the US elderberry industry. Agrofor. Syst..

[2] Masclaux-Daubresse C., Daniel-Vedele F., Dechorgnat J., Chardon F., Gaufichon L., Suzuki A. (2010). Nitrogen uptake, assimilation and remobilization in plants: Challenges for sustainable and productive agriculture. Ann. Bot..

[3] Miller A.J., Fan X., Orsel M., Smith S.J., Wells D.M. (2007). Nitrate transport and signalling. J. Exp. Bot..

[4] Bi Y.M., Meyer A., Downs G.S., Shi X., El-Kereamy A., Lukens L., Rothstein S.J. (2014). High throughput RNA sequencing of a hybrid maize and its parents shows different mechanisms responsive to nitrogen limitation. BMC Genom..

[5] Scheible W.R., Morcuende R., Czechowski T., Fritz C., Osuna D., Palacios-Rojas N., Schindelasch D., Thimm O., Udvardi M.K., Stitt M. (2004). Genome-wide reprogramming of primary and secondary metabolism, protein synthesis, cellular growth processes, and the regulatory infrastructure of Arabidopsis in response to nitrogen. Plant Physiol..

[6] Islam N., Li G., Garrett W.M., Lin R., Sriram G., Cooper B., Coleman G.D. (2015). Proteomics of nitrogen remobilization in poplar bark. J. Proteome Res..

[7] Wang X., Bian Y., Cheng K., Zou H., Sun S.S., He J.X. (2012). A comprehensive differential proteomic study of nitrate deprivation in Arabidopsis reveals complex regulatory networks of plant nitrogen responses. J. Proteome Res..

[8] Ma L., Sun X., Kong X., Galvan J.V., Li X., Yang S., Yang Y., Yang Y., Hu X. (2015). Physiological, biochemical and proteomics analysis reveals the adaptation strategies of the alpine plant *Potentilla saundersiana* at altitude gradient of the Northwestern Tibetan Plateau. J. Proteom..

[9] Jespersen D., Huang B. (2015). Proteins associated with heat-induced leaf senescence in creeping bentgrass as affected by foliar application of nitrogen, cytokinins, and an ethylene inhibitor. Proteomics.

[10] Tyers M., Mann M. (2003). From genomics to proteomics. Nature.

[11] Baginsky S., Gruissem W. (2006). *Arabidopsis thaliana* proteomics: From proteome to genome. J. Exp. Bot..

[12] Griffin T.J., Gygi S.P., Ideker T., Rist B., Eng J., Hood L., Aebersold R. (2002). Complementary profiling of gene expression at the transcriptome and proteome levels in *Saccharomyces cerevisiae*. Mol. Cell. Proteom..

[13] Tian Q., Stepaniants S.B., Mao M., Weng L., Feetham M.C., Doyle M.J., Yi E.C., Dai H., Thorsson V., Eng J. (2004). Integrated genomic and proteomic analyses of gene expression in Mammalian cells. Mol. Cell. Proteom..

[14] Washburn M.P., Koller A., Oshiro G., Ulaszek R.R., Plouffe D., Deciu C., Winzeler E., Yates J.R. (2003). Protein pathway and complex clustering of correlated mRNA and protein expression analyses in Saccharomyces cerevisiae. Proc. Natl. Acad. Sci. USA.

[15] Hajduch M., Hearne L.B., Miernyk J.A., Casteel J.E., Joshi T., Agrawal G.K., Song Z., Zhou M., Xu D., Thelen J.J. (2010). Systems analysis of seed filling in Arabidopsis: Using general linear modeling to assess concordance of transcript and protein expression. Plant Physiol..

[16] Selbach M., Schwanhausser B., Thierfelder N., Fang Z., Khanin R., Rajewsky N. (2008). Widespread changes in protein synthesis induced by microRNAs. Nature.

[17] Byers P.L., Thomas A.L. (2011). ‘Bob Gordon’ Elderberry. J. Am. Pomol. Soc..

[18] Byers P.L., Thomas A.L., Millican M. (2010). ‘Wyldewood’ Elderberry. Hortscience.

[19] Loomis R.S. (1997). On the utility of nitrogen in leaves. Proc. Natl. Acad. Sci. USA.

[20] Johnson M.C., Thomas A.L., Greenlief C.M. (2015). Impact of frozen storage on the anthocyanin and polyphenol contents of American elderberry fruit juice. J. Agric. Food Chem..

[21] Hurkman W.J., Tanaka C.K. (1986). Solubilization of plant membrane proteins for analysis by two-dimensional gel electrophoresis. Plant Physiol..

[22] Mooney B.P., Miernyk J.A., Greenlief C.M., Thelen J.J. (2006). Using quantitative proteomics of Arabidopsis roots and leaves to predict metabolic activity. Physiol. Plant..

[23] Hajduch M., Ganapathy A., Stein J.W., Thelen J.J. (2005). A systematic proteomic study of seed filling in soybean. Establishment of high-resolution two-dimensional reference maps, expression profiles, and an interactive proteome database. Plant Physiol..

[24] Dahal D., Newton K.J., Mooney B.P. (2016). Quantitative proteomics of *Zea mays* hybrids exhibiting different levels of heterosis. J. Proteome Res..

[25] Nesvizhskii A.I., Keller A., Kolker E., Aebersold R. (2003). A statistical model for identifying proteins by tandem mass spectrometry. Anal. Chem..

[26] Keller A., Nesvizhskii A.I., Kolker E., Aebersold R. (2002). Empirical statistical model to estimate the accuracy of peptide identifications made by MS/MS and database search. Anal. Chem..

[27] Caraux G., Pinloche S. (2005). PermutMatrix: A graphical environment to arrange gene expression profiles in optimal linear order. Bioinformatics.

[28] Usadel B., Nagel A., Thimm O., Redestig H., Blaesing O.E., Palacios-Rojas N., Selbig J., Hannemann J., Piques M.C., Steinhauser D. (2005). Extension of the visualization tool MapMan to allow statistical analysis of arrays, display of corresponding genes, and comparison with known responses. Plant Physiol..

[29] Heazlewood J.L., Verboom R.E., Tonti-Filippini J., Small I., Millar A.H. (2007). SUBA: The Arabidopsis subcellular database. Nucleic Acids Res..

[30] Shang C., Van Damme E.J. (2014). Comparative analysis of carbohydrate binding properties of *Sambucus nigra* lectins and ribosome-inactivating proteins. Glycoconj. J..

[31] Finn C.E., Thomas A.L., Byers P.L., Serce S. (2008). Evaluation of American (*Sambucus canadensis*) and European (*S. nigra*) elderberry genotypes grown in diverse environments and implications for cultivar development. Hortscience.

[32] Wase N., Black P.N., Stanley B.A., Di Russo C.C. (2014). Integrated quantitative analysis of nitrogen stress response in *Chlamydomonas reinhardtii* using metabolite and protein profiling. J. Proteome Res..

[33] Bahrman N., Le Gouis J., Negroni L., Amilhat L., Leroy P., Laine A.L., Jaminon O. (2004). Differential protein expression assessed by two-dimensional gel electrophoresis for two wheat varieties grown at four nitrogen levels. Proteomics.

[34] Yousuf P.Y., Ganie A.H., Khan I., Qureshi M.I., Ibrahim M.M., Sarwat M., Iqbal M., Ahmad A. (2016). Nitrogen-efficient and nitrogen-inefficient Indian mustard showed differential expression pattern of proteins in response to elevated CO_2_ and low nitrogen. Front. Plant Sci..

[35] Turkina M.V., Kargul J., Blanco-Rivero A., Villarejo A., Barber J., Vener A.V. (2006). Environmentally modulated phosphoproteome of photosynthetic membranes in the green alga *Chlamydomonas reinhardtii*. Mol. Cell. Proteom..

[36] Juergens M.T., Deshpande R.R., Lucker B.F., Park J.J., Wang H., Gargouri M., Holguin F.O., Disbrow B., Schaub T., Skepper J.N. (2015). The regulation of photosynthetic structure and function during nitrogen deprivation in *Chlamydomonas reinhardtii*. Plant Physiol..

[37] Wei S., Wang X., Zhang J., Liu P., Zhao B., Li G., Dong S. (2015). The role of nitrogen in leaf senescence of summer maize and analysis of underlying mechanisms using comparative proteomics. Plant Sci..

[38] Cheng L., Fuchigami L.H. (2000). Rubisco activation state decreases with increasing nitrogen content in apple leaves. J. Exp. Bot..

[39] Chen J.W., Yang Z.Q., Zhou P., Hai M.R., Tang T.X., Liang Y.L., An T.X. (2013). Biomass accumulation and partitioning, photosynthesis, and photosynthetic induction in field-grown maize (*Zea mays* L.) under low- and high-nitrogen conditions. Acta Physiol. Plant..

[40] Ding L., Gao L., Liu W., Wang M., Gu M., Ren B., Xu G., Shen Q., Guo S. (2016). Aquaporin plays an important role in mediating chloroplastic CO_2_ concentration under high-N supply in rice (*Oryza sativa*) plants. Physiol. Plant..

[41] Yagi N., Takeda S., Matsumoto N., Okada K. (2009). VAJ/GFA1/CLO is involved in the directional control of floral organ growth. Plant Cell Physiol..

[42] Jackson R.J., Hellen C.U., Pestova T.V. (2010). The mechanism of eukaryotic translation initiation and principles of its regulation. Nat. Rev. Mol. Cell Biol..

[43] Ursin V.M., Irvine J.M., Hiatt W.R., Shewmaker C.K. (1991). Developmental analysis of elongation factor-1 alpha expression in transgenic tobacco. Plant Cell.

[44] Horiguchi G., Molla-Morales A., Perez-Perez J.M., Kojima K., Robles P., Ponce M.R., Micol J.L., Tsukaya H. (2011). Differential contributions of ribosomal protein genes to *Arabidopsis thaliana* leaf development. Plant J..

[45] Saibil H. (2013). Chaperone machines for protein folding, unfolding and disaggregation. Nat. Rev. Mol. Cell Biol..

[46] Niforou K., Cheimonidou C., Trougakos I.P. (2014). Molecular chaperones and proteostasis regulation during redox imbalance. Redox Biol..

[47] Kurepa J., Toh E.A., Smalle J.A. (2008). 26S proteasome regulatory particle mutants have increased oxidative stress tolerance. Plant J..

[48] Pais S.M., Tellez-Inon M.T., Capiati D.A. (2009). Serine/threonine protein phosphatases type 2A and their roles in stress signaling. Plant Signal. Behav..

[49] Pratelli R., Pilot G. (2014). Regulation of amino acid metabolic enzymes and transporters in plants. J. Exp. Bot..

[50] Tyagi R., Lee Y.T., Guddat L.W., Duggleby R.G. (2005). Probing the mechanism of the bifunctional enzyme ketol-acid reductoisomerase by site-directed mutagenesis of the active site. FEBS J..

[51] Miesak B.H., Coruzzi G.M. (2002). Molecular and physiological analysis of Arabidopsis mutants defective in cytosolic or chloroplastic aspartate aminotransferase. Plant Physiol..

[52] Liao C., Peng Y., Ma W., Liu R., Li C., Li X. (2012). Proteomic analysis revealed nitrogen-mediated metabolic, developmental, and hormonal regulation of maize (*Zea mays* L.) ear growth. J. Exp. Bot..

[53] Seebauer J.R., Moose S.P., Fabbri B.J., Crossland L.D., Below F.E. (2004). Amino acid metabolism in maize earshoots. Implications for assimilate preconditioning and nitrogen signaling. Plant Physiol..

[54] Canas R.A., Amiour N., Quillere I., Hirel B. (2011). An integrated statistical analysis of the genetic variability of nitrogen metabolism in the ear of three maize inbred lines (*Zea mays* L.). J. Exp. Bot..

[55] Kocsy G., Tari I., Vankova R., Zechmann B., Gulyas Z., Poor P., Galiba G. (2013). Redox control of plant growth and development. Plant Sci..

[56] Foyer C.H., Noctor G. (2011). Ascorbate and glutathione: The heart of the redox hub. Plant Physiol..

[57] Begara-Morales J.C., Sanchez-Calvo B., Chaki M., Mata-Perez C., Valderrama R., Padilla M.N., Lopez-Jaramillo J., Luque F., Corpas F.J., Barroso J.B. (2015). Differential molecular response of monodehydroascorbate reductase and glutathione reductase by nitration and S-nitrosylation. J. Exp. Bot..

[58] Wheeler G.L., Jones M.A., Smirnoff N. (1998). The biosynthetic pathway of vitamin C in higher plants. Nature.

[59] Rhee S.G., Kang S.W., Jeong W., Chang T.S., Yang K.S., Woo H.A. (2005). Intracellular messenger function of hydrogen peroxide and its regulation by peroxiredoxins. Curr. Opin. Cell Biol..

[60] Yang T., Poovaiah B.W. (2002). Hydrogen peroxide homeostasis: Activation of plant catalase by calcium/calmodulin. Proc. Natl. Acad. Sci. USA.

[61] Smith L.M., Kelleher N.L., Linial M., Goodlett D., Langridge-Smith P., Goo Y.A., Safford G., Bonilla L., Kruppa G., Zubarev R. (2013). Proteoform: A single term describing protein complexity. Nat. Methods.

[62] Toby T.K., Fornelli L., Kelleher N.L. (2016). Progress in top-down proteomics and the analysis of proteoforms. Annu. Rev. Anal. Chem..

[63] Dahal D., Mooney B.P., Newton K.J. (2012). Specific changes in total and mitochondrial proteomes are associated with higher levels of heterosis in maize hybrids. Plant J..

[64] Prinsi B., Espen L. (2015). Mineral nitrogen sources differently affect root glutamine synthetase isoforms and amino acid balance among organs in maize. BMC Plant Biol..

[65] Wang X., Wei Y., Shi L., Ma X., Theg S.M. (2015). New isoforms and assembly of glutamine synthetase in the leaf of wheat (*Triticum aestivum* L.). J. Exp. Bot..

[66] Colignon B., Raes M., Dieu M., Delaive E., Mauro S. (2013). Evaluation of three-dimensional gel electrophoresis to improve quantitative profiling of complex proteomes. Proteomics.

[67] Buchanan B.B., Balmer Y. (2005). Redox regulation: A broadening horizon. Annu. Rev. Plant Biol..

[68] Bykova N.V., Rampitsch C. (2013). Modulating protein function through reversible oxidation: Redox-mediated processes in plants revealed through proteomics. Proteomics.

[69] Racchi M.L. (2013). Antioxidant defenses in plants with attention to prunus and citrus spp. Antioxidants.

[70] Thornalley P.J. (1996). Pharmacology of methylglyoxal: Formation, modification of proteins and nucleic acids, and enzymatic detoxification—A role in pathogenesis and antiproliferative chemotherapy. Gen. Pharmacol..

[71] Krapp A. (2015). Plant nitrogen assimilation and its regulation: A complex puzzle with missing pieces. Curr. Opin. Plant Biol..

