# Supplementary material for: Comparative Proteomic Analysis Unveils Critical Pathways Underlying the Role of Nitrogen Fertilizer Treatment in American Elderberry
Source: Proteomes. 2019 Mar 20;7(1):10. doi: 10.3390/proteomes7010010 (PMC6473435; doi:10.3390/proteomes7010010)
Supplement: Supplementary file 1 [file proteomes-07-00010-s001.pdf]

SUPPORTING INFORMATION

Comparative proteomic analysis unveils critical pathways underlying the role of nitrogen fertilizer treatment in American elderberry

Bo Yang[1], Andrew L. Thomas[2], and C. Michael Greenlief [1, †]

[1]Department of Chemistry, University of Missouri, Columbia, Missouri 65211, USA

[2]Division of Plant Sciences, Southwest Research Center, University of Missouri, Mt. Vernon, Missouri 65712, USA

[†]Corresponding author: C. Michael Greenlief

Phone: 573-882-3288; Fax: 573-882-2754;

E-mail: greenliefm@missouri.edu

TABLE OF CONTENTS

Figure S1. Scatter graph of relative spots volumes detected on 2-DE gels

Figure S2. Representative 2-DE maps of differentially expressed proteins

Figure S3. PCA analysis of differentially expressed proteins

Figure S4. Mercator protein functional categorization of each genotype

Table S1. Statistical analysis of differentially expressed proteins

Table S2. Protein report for MALDI-TOF/TOF

Table S3. Protein report for LC-MS/MS

Table S4. Identification of altered proteins after 56 kg N/ha treatment

Table S5. Identification of altered proteins after 112 kg N/ha treatment

Table S6. Identification of altered proteins after 169 kg N/ha treatment

Figure S1. Scatter graph based on the ratios of relative spots volumes detected in the master gel (y-axis) and the respective replicates (x-axis). (**A-D**): 0, 56, 112 and 169 kg N/ha treated Adams II. (**E-H**): 0, 56, 112 and 169 kg N/ha treated Bob Gordon. (**I-L**): 0, 56, 112 and 169 kg N/ha treated Wyldewood.

A

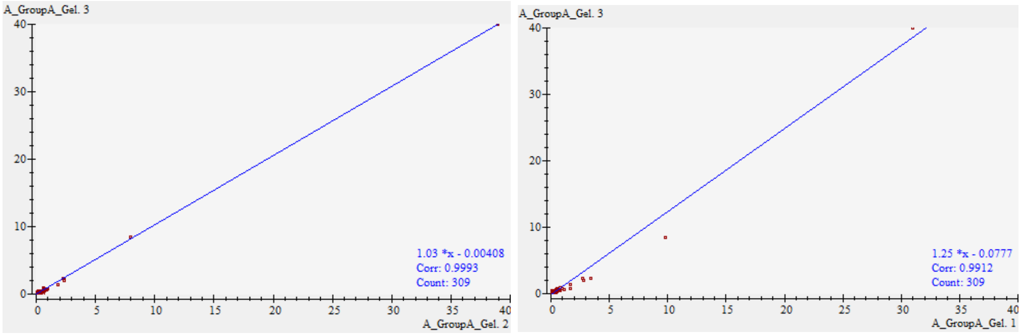

B

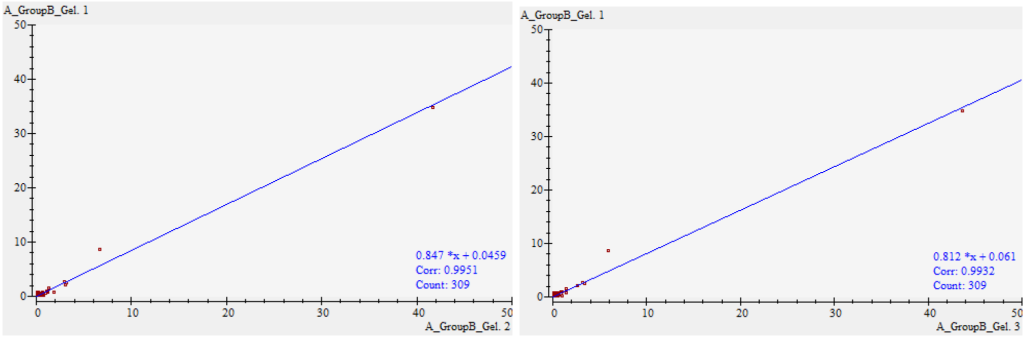

C

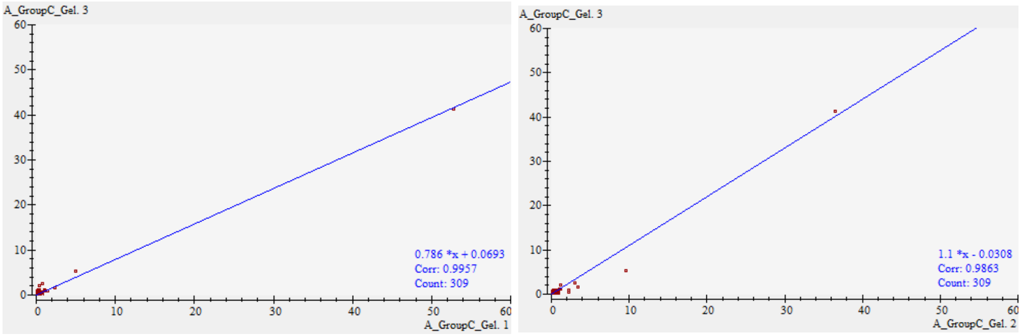

D

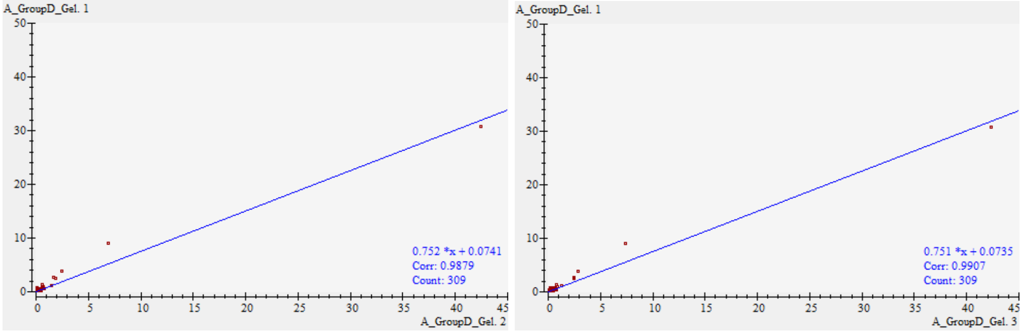
A_GroupD_Gel. 1
0.752 *x + 0.0741
Corr: 0.9879
Count: 309
A_GroupD_Gel. 2
A_GroupD_Gel. 1
0.751 *x + 0.0735
Corr: 0.9907
Count: 309
A_GroupD_Gel. 3

E

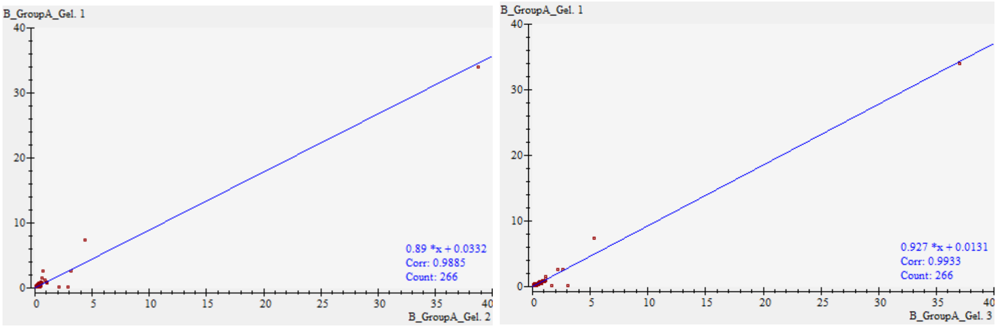
B_GroupA_Gel. 1
0.89 *x + 0.0332
Corr: 0.9885
Count: 266
B_GroupA_Gel. 2
B_GroupA_Gel. 1
0.927 *x + 0.0131
Corr: 0.9933
Count: 266
B_GroupA_Gel. 3

F

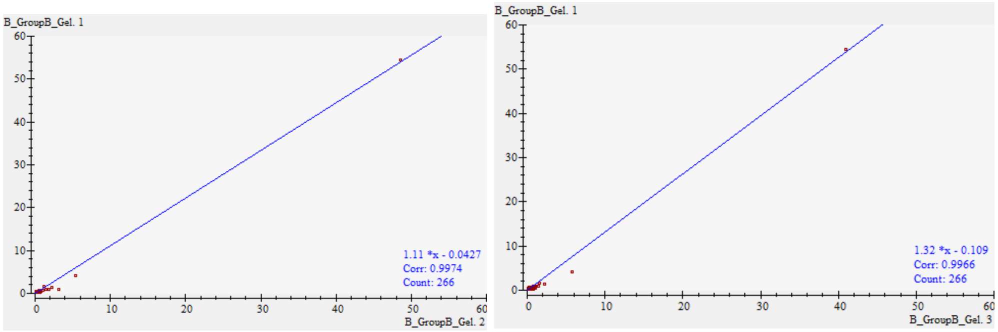
B_GroupB_Gel. 1
1.11 *x - 0.0427
Corr: 0.9974
Count: 266
B_GroupB_Gel. 2
B_GroupB_Gel. 1
1.32 *x - 0.109
Corr: 0.9966
Count: 266
B_GroupB_Gel. 3

G

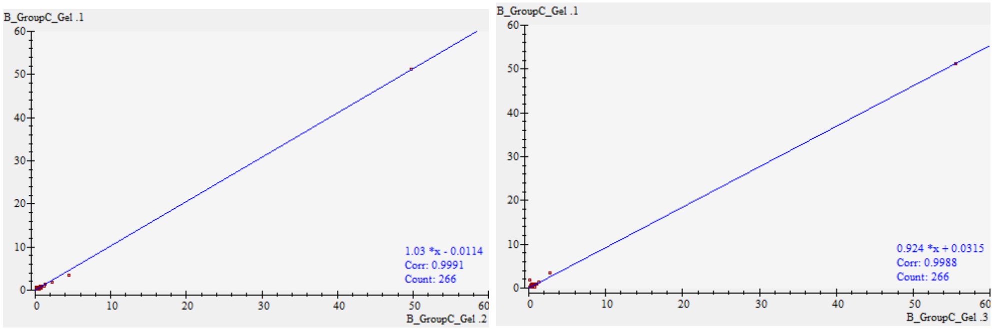
B_GroupC_Gel .1
1.03 *x - 0.0114
Corr: 0.9991
Count: 266
B_GroupC_Gel .2
B_GroupC_Gel .1
0.924 *x + 0.0315
Corr: 0.9988
Count: 266
B_GroupC_Gel .3

H

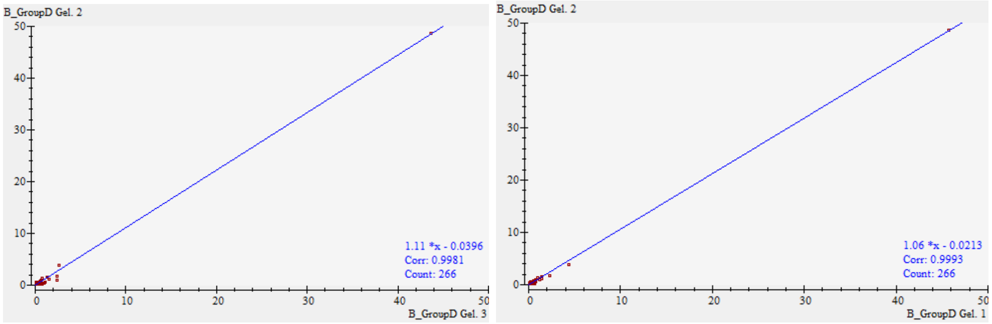
B_GroupD Gel. 2
B_GroupD Gel. 3
1.11 *x - 0.0396
Corr: 0.9981
Count: 266
B_GroupD Gel. 2
B_GroupD Gel. 1
1.06 *x - 0.0213
Corr: 0.9993
Count: 266

I

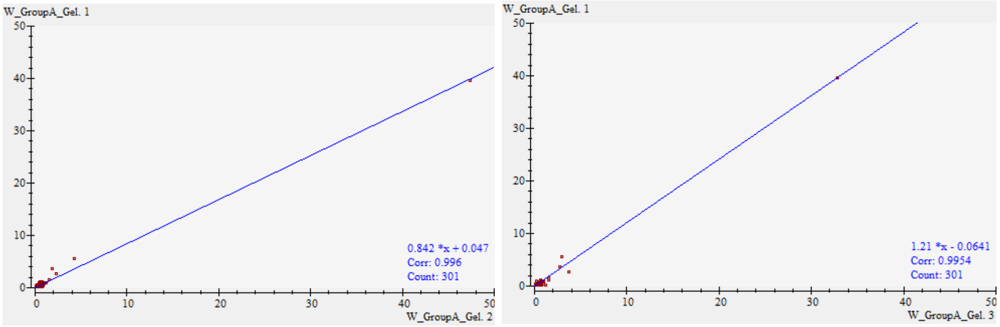
W_GroupA_Gel. 1
W_GroupA_Gel. 2
0.842 *x + 0.047
Corr: 0.996
Count: 301
W_GroupA_Gel. 1
W_GroupA_Gel. 3
1.21 *x - 0.0641
Corr: 0.9954
Count: 301

J

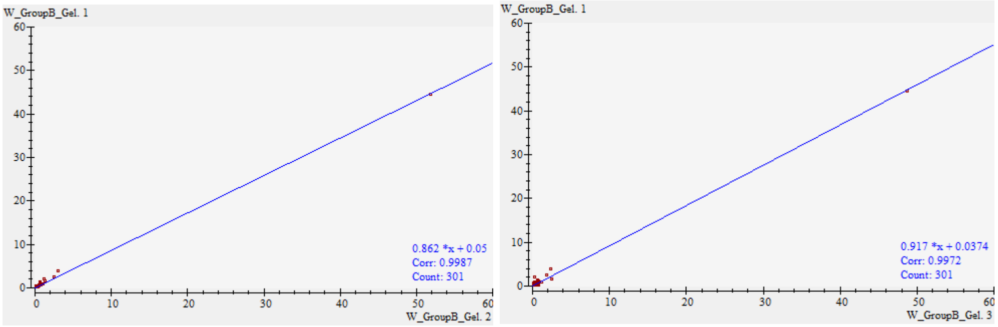
W_GroupB_Gel. 1
W_GroupB_Gel. 2
0.862 *x + 0.05
Corr: 0.9987
Count: 301
W_GroupB_Gel. 1
W_GroupB_Gel. 3
0.917 *x + 0.0374
Corr: 0.9972
Count: 301

K

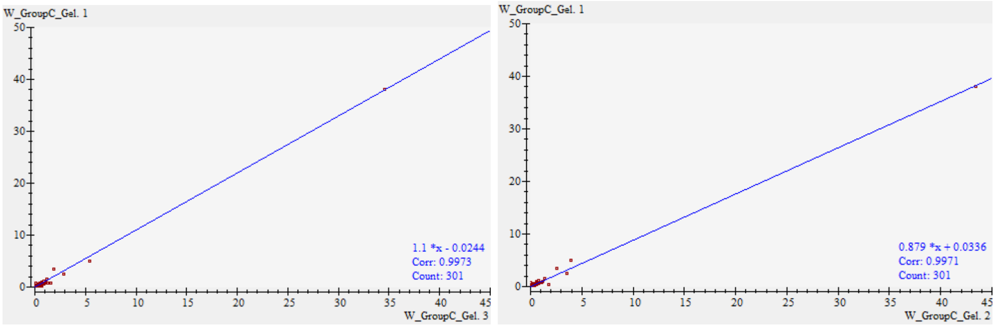
W_GroupC_Gel. 1
W_GroupC_Gel. 3
1.1 *x - 0.0244
Corr: 0.9973
Count: 301
W_GroupC_Gel. 1
W_GroupC_Gel. 2
0.879 *x + 0.0336
Corr: 0.9971
Count: 301

L

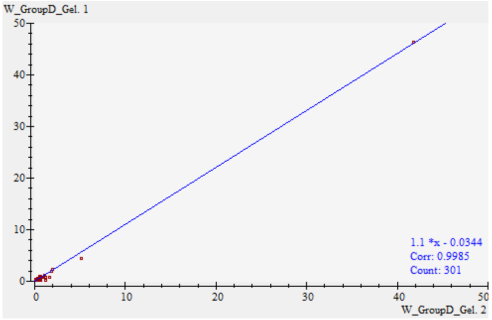
W_GroupD_Gel. 1
1.1 *x - 0.0344
Corr: 0.9985
Count: 301
W_GroupD_Gel. 2

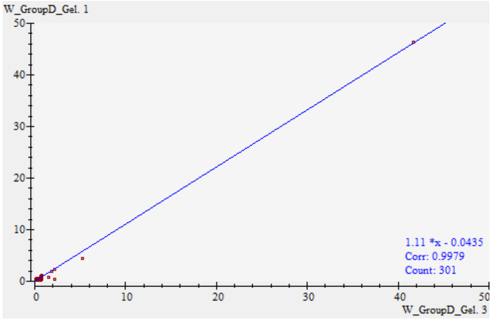
W_GroupD_Gel. 1
1.11 *x - 0.0435
Corr: 0.9979
Count: 301
W_GroupD_Gel. 3

Figure S2. Representative 2-DE maps of proteins extracted from American elderberry leaves. In IEF, a total of 600 μg of protein was loaded onto each pH 3-10 IPG strip (13 cm, linear). 13.5% SDS-PAGE gels were used for second dimension separation. Coomassie brilliant blue G-250 was used to visualize protein spots. Two-way ANOVA analysis indicated a total of 165 spots had significant intensity changes under different N fertilizer conditions ($p < 0.05$, more than 2-fold). 2D gels from samples (**A**) 0 kg N/ha treated Bob Gordon, (**B**) 0 kg N/ha treated Wyldewood, (**C**) 0 kg N/ha treated Adams II, (**D**) 56 kg N/ha treated Adams II, (**E**) 169 kg N/ha treated Wyldewood, and (**F**) 112 kg N/ha treated Adams II.

A

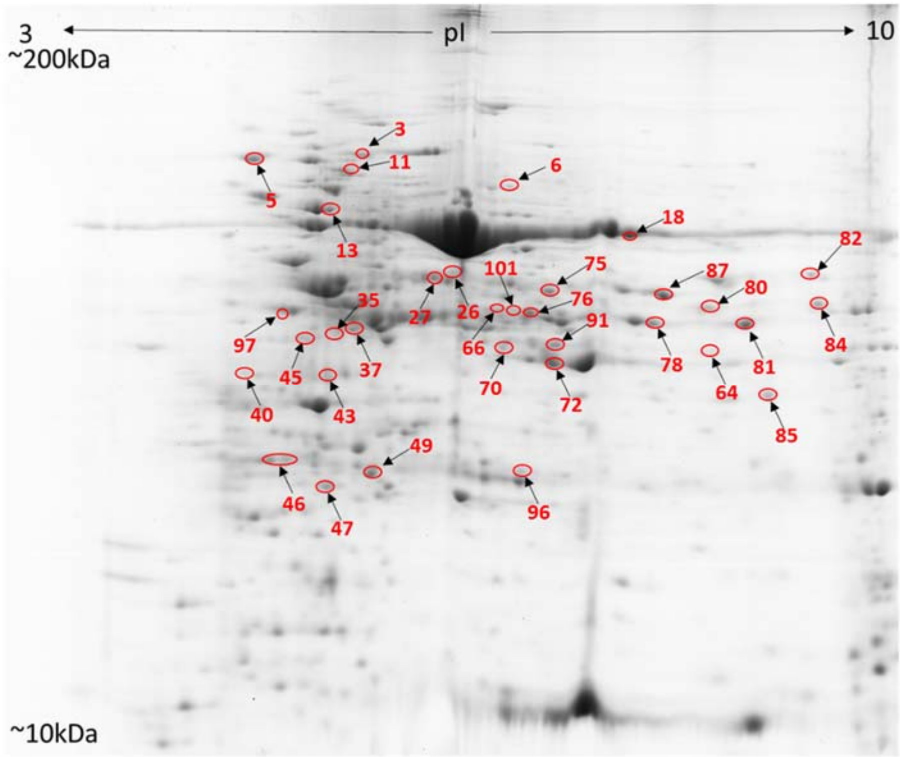

B

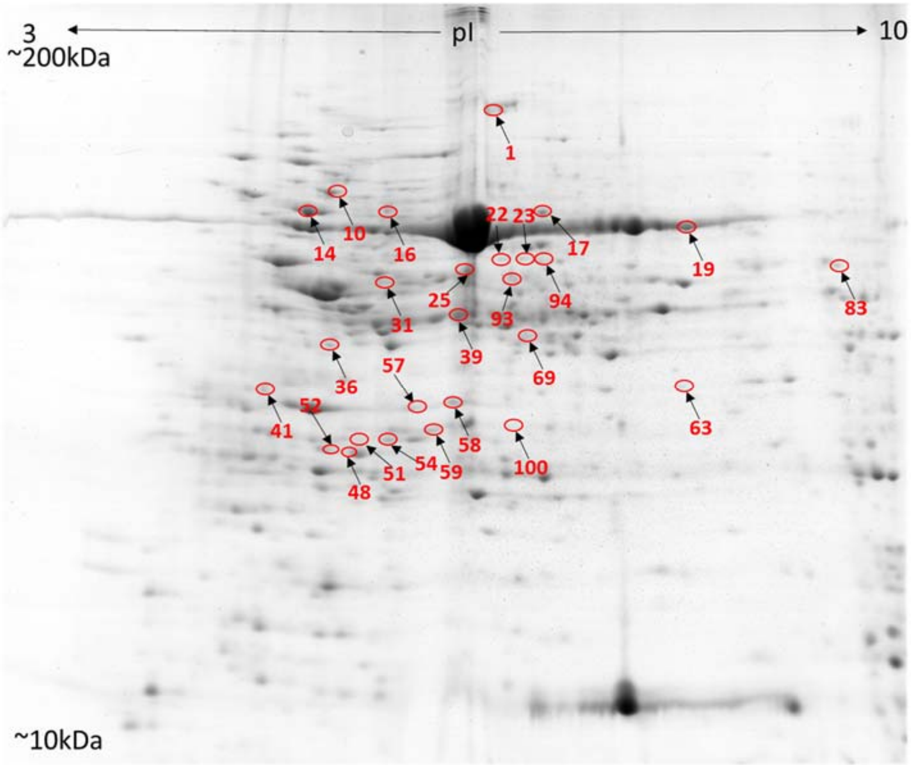

3
pI
10
~200kDa
~10kDa
1
10
14
16
22
23
17
19
83
25
93
94
31
39
69
36
57
41
52
58
63
100
51
54
59
48

C

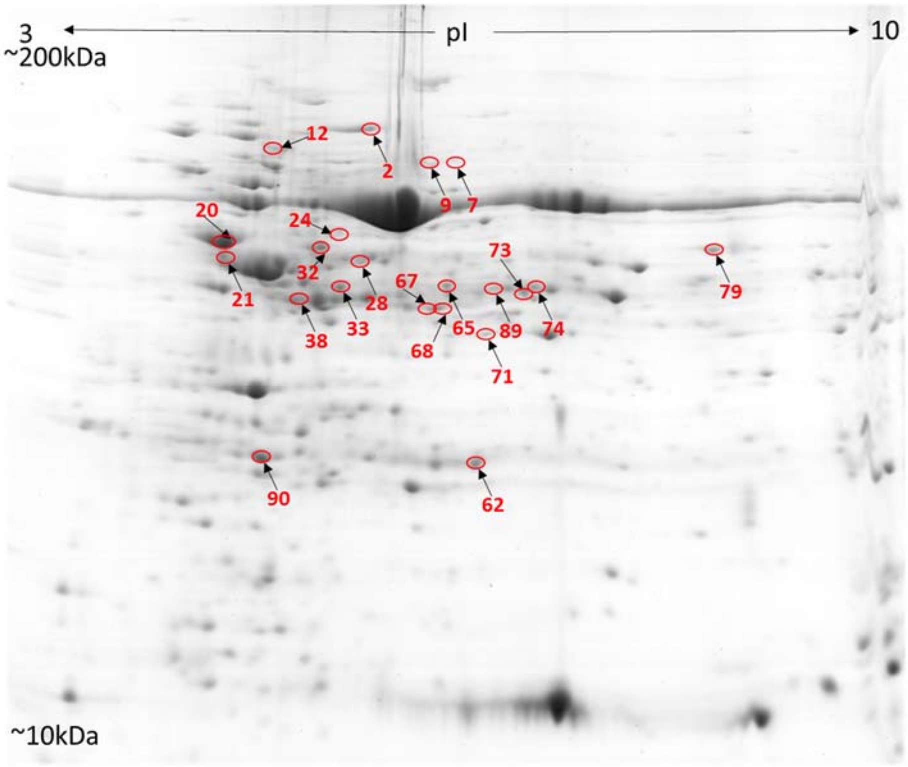
3
pI
10
~200kDa
~10kDa
12
2
9
7
20
24
32
21
28
33
38
67
65
68
73
89
74
71
79
90
62

D

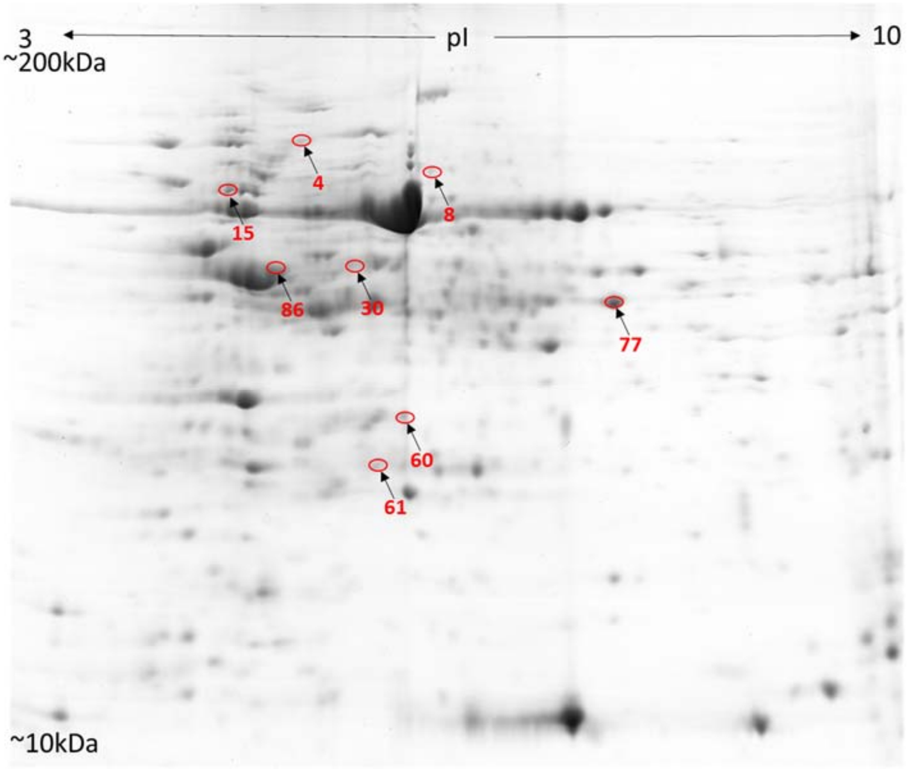
3
pI
10
~200kDa
~10kDa
4
8
15
86
30
77
60
61

E

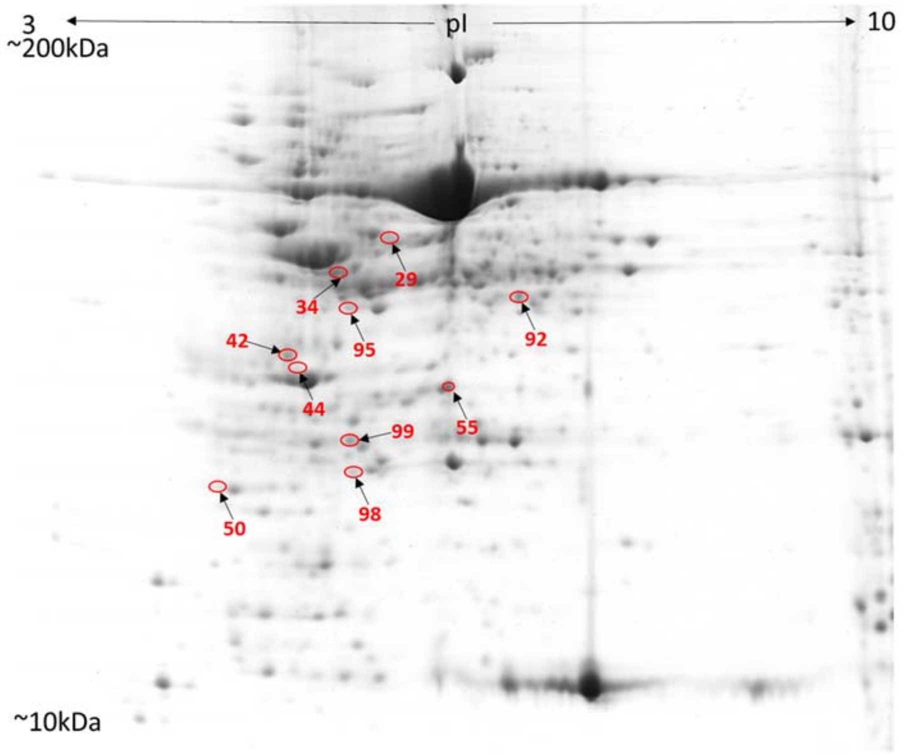
3
pI
10
~200kDa
29
34
95
92
42
44
55
99
98
50
~10kDa

F

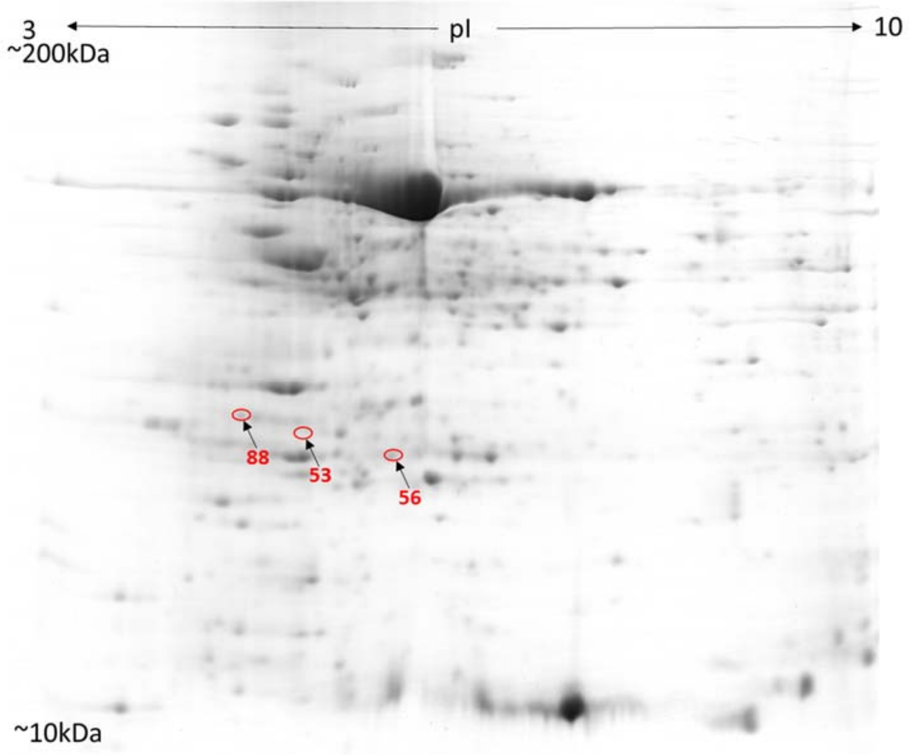
3
pI
10
~200kDa
88
53
56
~10kDa

Figure S3. Principal component analysis (PCA) of the 165 differential protein expression profile across three groups (Group 1: 0 (or control) *vs* 56 kg N/ha; Group 2: 0 *vs* 112 kg N/ha; Group 3: 0 *vs* 169 kg N/ha). PCA reveals almost indistinguishable features of N treatment of Adams II and Wyldewood, whereas a clear separation was detected between Bob Gordon and Adams II or Wyldewood. PC1 represents the variance as a result of technical and biological variations. PC2 represents the variance as a result of applied treatment. The ratios for x and y axis indicate the percentage of variance explained by PC1 and PC2.

Group 1:

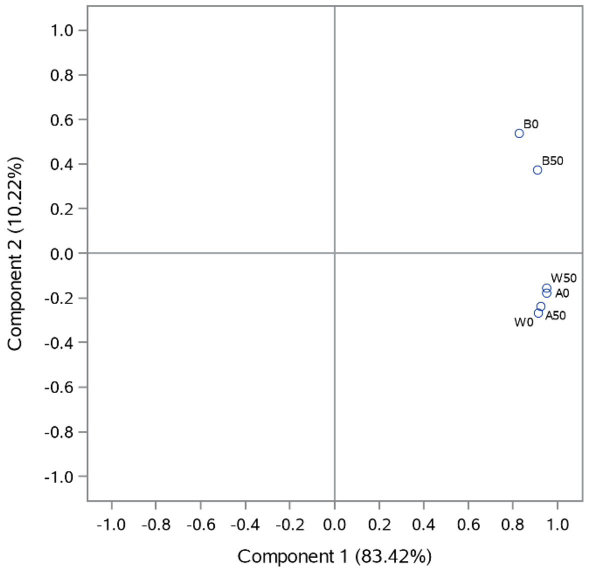

Group 2:

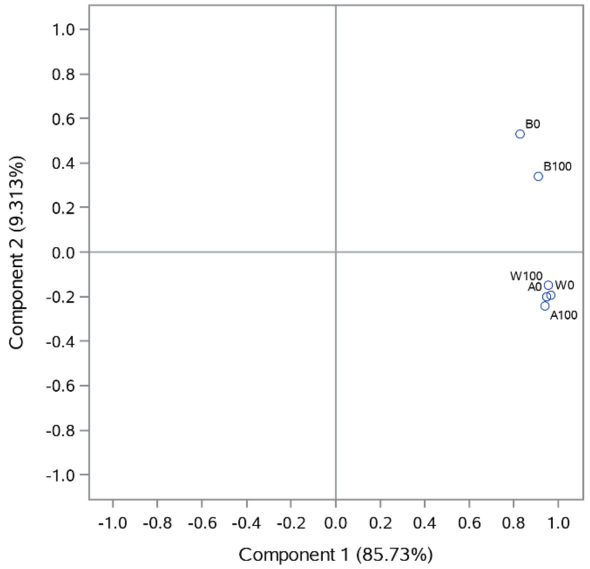

Group 3:

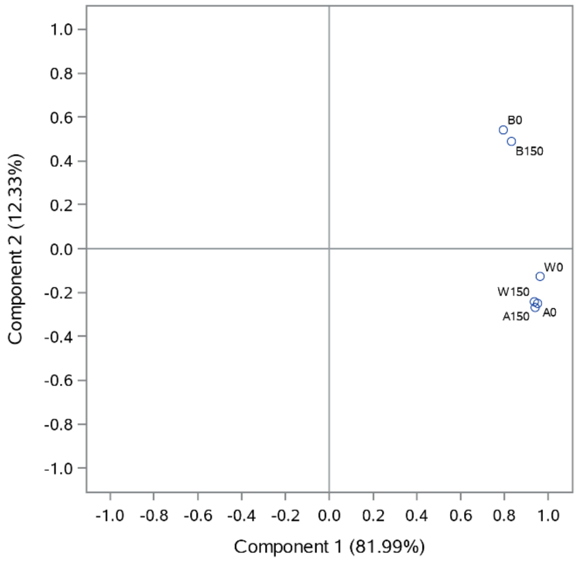

1.0
0.8
0.6
0.4
0.2
0.0
-0.2
-0.4
-0.6
-0.8
-1.0
Component 2 (12.33%)
B0
B150
W0
W150
A150
A0
-1.0
-0.8
-0.6
-0.4
-0.2
0.0
0.2
0.4
0.6
0.8
1.0
Component 1 (81.99%)

Figure S4. Mercator protein functional categorization of up or down-regulated differentially expressed proteins across three N treatments of each genotype analyzed.

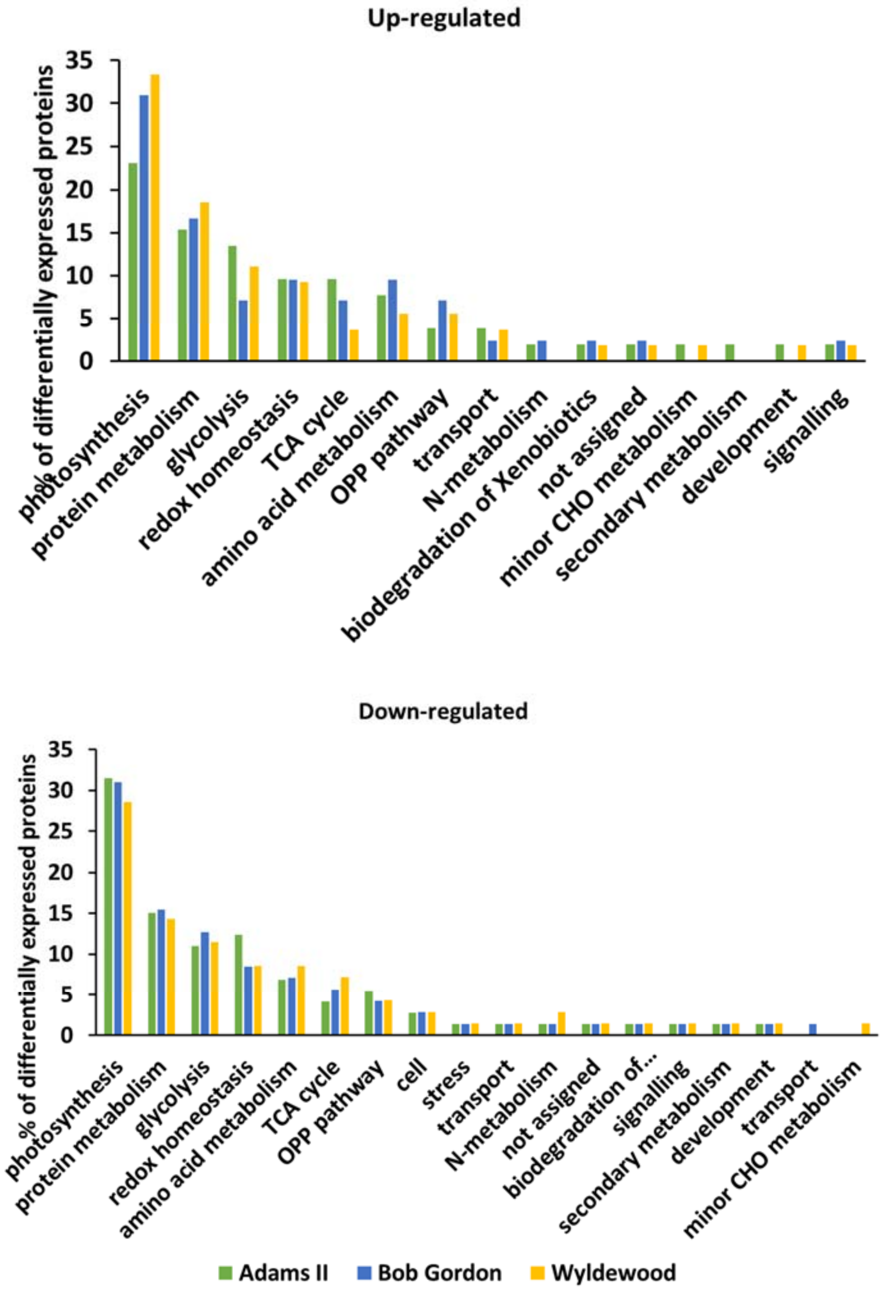

Table S1. Statistical Analysis of 165 differentially expressed proteins in three groups (Group 1: 0 (or control) *vs* 56 kg N/ha; Group 2: 0 *vs* 112 kg N/ha; Group 3: 0 *vs* 169 kg N/ha).

Group 1:

Pos. No. [a]	Log_2(Average Ratio) [b]									One-way ANOVA p Values [c]	Two-way ANOVA p Values [d]		
	W56/W0	B56/B0	A56/A0	B0/A0	W0/A0	B0/W0	W56/A56	B56/A56	B56/W56		Genotype	Nitrogen	Interact
2	**1.19**	0.18	0.57	0.45	-0.66	1.12	-0.04	0.06	0.10	0.0268	0.1120	**0.0065**	0.2642
3	**1.16**	0.40	0.28	0.40	-0.95	**1.35**	-0.06	0.53	0.59	0.0025	**0.0020**	**0.0046**	0.3881
4	1.41	0.79	-0.18	0.10	-1.07	1.16	0.53	**1.07**	0.54	0.0488	0.0812	**0.0401**	0.1696
6	0.22	-0.82	-0.06	-0.12	**-1.35**	**1.23**	**-1.08**	-0.89	0.19	0.0021	**0.0006**	0.1616	0.0976
8	-0.26	-0.16	0.46	-1.41	0.10	**-1.51**	-0.63	**-2.03**	-1.41	0.0024	**0.0004**	0.6713	0.2053
9	0.08	-0.01	-0.42	-1.06	0.09	-1.15	0.58	-0.66	**-1.24**	0.0192	**0.0031**	0.5766	0.5326
10	0.02	-0.41	-0.45	0.71	-0.36	**1.07**	0.11	0.75	0.64	0.0335	**0.0089**	0.1716	0.5415
11	-0.11	-0.64	-0.47	**0.93**	-0.21	**1.13**	0.16	0.76	0.60	0.0097	**0.0040**	0.0506	0.3041
13	0.90	0.03	**-2.40**	-1.26	**-2.12**	0.86	1.19	1.17	-0.02	0.0145	0.1855	0.0946	**0.0067**
14	0.18	-0.45	**-1.08**	-0.30	**-1.35**	**1.06**	-0.09	0.34	0.43	0.0008	0.0028	0.0036	**0.0103**
15	0.24	0.31	**1.81**	0.31	-0.94	1.25	**-2.51**	-1.19	1.32	0.0141	**0.0211**	**0.0394**	0.0664
18	0.15	-0.47	-0.03	0.55	-0.86	**1.41**	-0.69	0.11	0.80	0.0074	**0.0015**	0.3119	0.2601
19	-0.32	-0.03	-1.10	-0.69	0.44	**-1.13**	1.23	0.38	-0.84	0.0199	**0.0083**	0.0745	0.3399
20	-0.53	-0.41	0.04	**-1.00**	-0.55	-0.44	**-1.11**	**-1.44**	-0.33	0.0001	**0.0001**	0.1241	0.3325
25	**-0.75**	0.66	-0.39	**-1.46**	0.53	**-1.99**	0.18	-0.41	-0.59	0.0016	0.0008	0.0827	**0.0342**
27	-0.31	-0.31	-0.41	**0.91**	0.14	0.76	0.25	1.00	0.76	0.0195	**0.0040**	0.1280	0.9253
29	-0.24	-0.68	0.25	**1.72**	0.76	0.96	0.27	0.79	0.52	0.0457	**0.0148**	0.2724	0.3254
30	0.16	0.20	-0.28	**-1.40**	**-0.79**	-0.60	-0.36	**-0.92**	-0.56	0.0001	**0.0001**	0.7310	0.1338
32	-0.23	-0.23	-0.37	**-1.13**	-0.43	-0.71	-0.29	**-0.99**	-0.70	0.0035	**0.0007**	0.0845	0.6526
33	-0.79	**-1.02**	0.31	0.20	-0.56	0.76	**-1.66**	**-1.14**	0.52	0.0010	0.0010	0.0452	**0.0113**
35	0.66	**0.75**	-0.02	0.57	-0.46	**1.03**	0.22	**1.34**	**1.12**	0.0001	0.0001	0.0034	**0.0229**
36	-0.27	1.32	0.09	0.24	**2.26**	**-2.02**	**1.90**	1.48	-0.43	0.0020	**0.0004**	0.5040	0.1333
37	-0.52	-0.45	-0.61	**0.73**	0.12	0.61	0.21	0.89	0.68	0.0068	**0.0036**	**0.0105**	0.9182
38	**-1.50**	0.01	-0.56	-0.84	-0.14	-0.70	-1.08	-0.27	0.81	0.0106	0.0630	**0.0064**	0.0631
39	**-1.30**	-1.21	-0.79	1.09	**2.00**	**-0.91**	1.49	0.67	-0.82	0.0008	**0.0012**	**0.0018**	0.0953
40	**1.42**	-0.18	-0.82	**1.71**	-0.10	**1.81**	**2.15**	**2.35**	0.20	0.0000	0.0001	0.2845	**0.0043**
41	-0.13	0.39	-0.38	0.33	1.05	-0.72	**1.29**	1.09	-0.20	0.0398	**0.0072**	0.9800	0.4962
42	**-1.02**	0.53	-0.44	0.80	1.20	-0.40	0.62	**1.77**	**1.15**	0.0007	0.0006	0.2882	**0.0052**
43	-0.68	**-1.35**	-0.14	**1.44**	-0.07	**1.51**	-0.61	0.23	0.84	0.0029	0.0047	0.0114	**0.0419**
46	-0.27	-0.74	-0.70	-0.02	**-1.02**	**1.00**	-0.60	-0.07	0.53	0.0050	**0.0070**	**0.0048**	0.2664
47	0.67	**-1.56**	-1.68	0.32	-0.58	0.90	1.78	0.45	-1.32	0.0338	0.5346	0.0416	**0.0192**
48	-0.85	0.13	-0.35	-0.38	1.22	**-1.60**	0.72	0.10	-0.61	0.0306	**0.0136**	0.1518	0.2256
49	0.71	-0.37	-1.42	**0.92**	-0.24	**1.15**	**1.89**	**1.97**	0.08	0.0003	0.0001	0.2061	**0.0062**
54	**-0.58**	-0.61	-0.10	**1.07**	**1.45**	-0.37	0.96	0.56	-0.40	0.0003	**0.0001**	**0.0050**	0.1324
55	0.10	1.11	-0.24	-1.41	0.26	**-1.67**	0.60	-0.06	-0.67	0.0407	**0.0117**	0.4112	0.2908

56	-0.57	-0.46	**-1.53**	**-1.08**	**-1.35**	0.28	-0.39	-0.01	0.38	0.0014	0.0078	0.0018	**0.0225**
57	-0.31	-0.02	0.95	1.24	**1.82**	-0.57	0.56	0.27	-0.28	0.0148	**0.0037**	0.8420	0.1670
58	0.67	-0.03	0.28	-0.36	1.22	-1.58	1.61	-0.66	**-2.28**	0.0428	**0.0095**	0.3413	0.5322
59	-0.39	-1.40	-2.82	-0.86	0.74	-1.60	**3.17**	0.56	-2.61	0.0090	**0.0034**	**0.0318**	0.5816
60	-0.40	1.07	-0.28	-1.88	0.67	**-2.55**	0.56	-0.53	-1.09	0.0221	**0.0043**	0.6549	0.3582
61	-0.42	0.16	0.47	-0.54	0.01	-0.55	**-0.89**	-0.85	0.03	0.0422	**0.0198**	0.5437	0.1246
62	-0.56	**-1.04**	-0.94	0.15	-0.34	0.49	0.04	0.05	0.02	0.0148	0.4262	**0.0009**	0.4397
65	-0.55	-0.03	-0.25	-0.94	0.06	**-1.00**	-0.24	-0.72	-0.48	0.0332	**0.0115**	0.1194	0.4436
66	0.41	-0.16	0.23	0.74	-0.33	**1.07**	-0.15	0.35	0.50	0.0350	**0.0071**	0.5599	0.4445
67	-1.03	-0.26	-0.10	-0.36	**1.40**	**-1.76**	0.47	-0.52	-0.99	0.0070	**0.0039**	0.0643	0.1062
68	-0.38	-0.84	0.23	-0.03	**1.43**	**-1.46**	0.82	-1.10	-1.92	0.0133	**0.0022**	0.3537	0.5490
69	-0.90	-0.53	-2.03	1.44	**2.00**	-0.56	3.13	2.94	-0.19	0.0054	**0.0021**	**0.0229**	0.5378
70	0.01	-0.84	-0.37	**1.19**	0.99	0.20	**1.36**	0.72	-0.64	0.0060	**0.0024**	0.0744	0.1607
71	-0.65	**-1.11**	-0.10	**1.51**	0.79	0.72	0.24	0.50	0.26	0.0147	**0.0205**	**0.0204**	0.1385
72	-0.11	**-1.17**	0.18	**2.44**	0.44	**2.00**	0.15	1.09	0.94	0.0001	0.0001	0.0148	**0.0052**
73	-0.30	-0.73	-0.35	0.18	-0.71	**0.90**	-0.67	-0.20	0.46	0.0304	**0.0257**	**0.0271**	0.4173
74	-0.26	0.42	0.25	-1.12	**-1.38**	0.26	**-1.89**	**-0.95**	0.95	0.0018	**0.0002**	0.3776	0.5685
75	-0.29	**-1.29**	0.15	**0.66**	-0.54	**1.20**	-0.98	-0.77	0.20	0.0009	0.0044	0.0097	**0.0037**
76	-0.66	**-1.01**	-1.06	**1.77**	-0.02	**1.79**	0.37	**1.81**	**1.44**	0.0000	0.0001	0.0001	**0.0070**
77	-0.37	-0.13	0.27	**-0.77**	-0.28	-0.49	**-0.92**	**-1.17**	-0.25	0.0002	**0.0001**	0.8663	0.0654
78	-0.53	-0.24	-2.68	1.06	-0.12	1.18	2.03	**3.50**	1.47	0.0055	**0.0013**	0.0676	0.5779
79	1.97	-0.34	0.27	-0.15	**-4.44**	**4.30**	**-2.74**	**-0.76**	**1.98**	0.0000	0.0001	0.3276	**0.0135**
80	-0.51	-0.04	0.17	0.89	0.18	0.70	-0.49	0.68	**1.17**	0.0351	**0.0059**	0.6536	0.6166
81	0.11	-0.66	-0.41	**3.95**	0.59	**3.36**	1.11	**3.70**	**2.60**	0.0001	**0.0001**	0.1465	0.1474
82	**1.85**	-0.33	$+\infty$	$+\infty$	$+\infty$	**2.22**	**2.11**	**2.14**	0.03	0.0003	0.0001	0.0993	**0.0270**
86	-0.39	-0.46	-0.59	0.49	-0.16	**0.65**	0.04	0.62	0.59	0.0020	**0.0018**	**0.0023**	0.6936

Group 2:

Pos. No. [a]	Log_2(Average Ratio) [b]									One-way ANOVA p Values [c]	Two-way ANOVA p Values [d]		
	W112/W0	B112/B0	A112/A0	B0/A0	W0/A0	B0/W0	W112/A112	B112/A112	B112/W112		Genotype	Nitrogen	Interact
1	0.55	1.50	-0.54	-0.44	0.62	-1.06	1.72	**1.61**	-0.11	0.0411	0.0478	**0.0800**	0.1227
2	0.28	-0.02	-0.67	0.51	-0.68	**1.18**	0.26	**1.15**	0.89	0.0115	**0.0021**	0.5385	0.3338
3	-0.41	-0.29	**-1.21**	-0.11	**-1.25**	1.14	-0.45	0.80	1.25	0.0091	**0.0080**	**0.0193**	0.1843
5	-0.50	-0.43	-0.54	0.12	-0.54	**0.65**	-0.50	0.23	0.73	0.0243	0.0206	**0.0131**	0.8809
7	0.06	0.57	-0.81	**-1.82**	-0.69	-1.13	0.19	-0.44	-0.63	0.0365	**0.0187**	0.4240	0.1120
10	0.08	-0.62	-0.09	**0.71**	-0.37	**1.09**	-0.21	0.18	0.39	0.0121	**0.0063**	0.1175	0.1114
13	0.05	-0.02	**-2.09**	**-1.25**	**-2.13**	0.88	0.01	0.82	0.81	0.0019	0.0126	0.0134	**0.0050**
14	0.31	-0.36	**-1.60**	-0.29	-1.37	**1.08**	0.54	0.95	0.41	0.0223	0.0987	0.0350	**0.0366**
17	-0.02	-0.57	0.17	1.88	**2.81**	-0.93	**2.62**	1.14	**-1.48**	0.0013	**0.0001**	0.6116	0.7524
18	0.61	-0.75	-0.54	0.55	-0.88	**1.43**	0.27	0.34	0.07	0.0206	0.0279	0.1340	**0.0432**
20	-0.33	-0.31	-0.65	**-0.99**	-0.57	-0.43	-0.25	-0.65	-0.41	0.0111	**0.0069**	**0.0229**	0.3435
21	-0.85	-1.14	**-0.79**	**-0.84**	-0.65	-0.19	-0.70	-1.19	-0.49	0.0012	**0.0026**	**0.0006**	0.7059
22	0.32	0.31	0.00	-0.26	**0.74**	-1.00	1.06	0.05	-1.01	0.0251	**0.0043**	0.3328	0.7181
23	0.47	0.84	0.72	-0.76	0.96	-1.72	0.71	-0.64	**-1.35**	0.0223	**0.0062**	0.0685	0.9312
24	**1.04**	1.60	-0.72	-1.97	-0.21	-1.76	**1.55**	0.35	**-1.21**	0.0017	0.0026	0.0378	**0.0105**
25	0.01	0.90	-0.08	-1.46	0.51	**-1.97**	0.60	-0.47	**-1.07**	0.0020	**0.0003**	0.4944	0.4393
26	-0.31	0.56	-0.61	0.20	0.40	-0.21	0.70	**1.38**	0.67	0.0182	0.0130	0.8928	**0.0387**
27	-0.32	-0.50	-1.31	**0.91**	0.13	0.78	1.12	**1.72**	0.60	0.0030	**0.0013**	**0.0116**	0.5604
29	-0.11	-0.18	-0.61	**1.72**	0.74	**0.98**	1.24	**2.15**	**0.91**	0.0002	**0.0001**	0.2487	0.8847
30	-0.03	0.15	**-1.46**	**-1.39**	**-0.81**	-0.58	0.62	0.22	-0.40	0.0008	0.0126	0.0081	**0.0015**
31	0.74	-0.03	-0.20	0.00	0.41	-0.40	**1.35**	0.18	**-1.17**	0.0094	**0.0045**	0.1974	0.0787
32	-0.50	-0.67	**-1.88**	**-1.13**	-0.44	-0.69	0.94	0.08	-0.86	0.0004	0.0148	0.0002	**0.0121**
33	-0.57	-1.60	0.26	-0.20	-0.57	0.38	-1.40	**-2.05**	-0.65	0.0416	**0.0232**	0.2097	0.1588
35	0.03	**-1.05**	-0.85	**0.88**	-0.61	**1.48**	0.28	0.68	0.41	0.0012	0.0021	0.0045	**0.0363**
36	0.66	1.44	1.87	0.25	**2.25**	-2.00	1.05	-0.18	-1.22	0.0152	**0.0072**	**0.0208**	0.9426
40	-0.34	**-1.31**	-0.03	**1.72**	-0.11	**1.83**	-0.43	0.44	0.86	0.0000	0.0001	0.0003	**0.0002**
42	0.08	-0.10	0.10	0.81	1.19	-0.38	**1.17**	0.61	-0.56	0.0264	**0.0034**	0.9182	0.9109
43	0.90	**-1.11**	0.57	**1.44**	-0.01	**1.45**	0.32	-0.23	-0.55	0.0196	0.0897	0.8802	**0.0078**
44	**0.97**	0.19	0.49	-0.40	0.06	-0.46	0.54	-0.69	**-1.24**	0.0060	**0.0100**	**0.0087**	0.1088
46	-0.16	-0.75	**-2.18**	-0.02	**-1.04**	**1.02**	0.99	1.41	0.42	0.0015	0.0392	0.0007	**0.0204**
50	0.60	0.69	-1.49	**-1.92**	**-2.52**	0.60	-0.43	0.26	0.69	0.0296	0.0325	0.3254	**0.0443**
51	**-1.40**	0.22	-0.12	0.01	**1.22**	**-1.21**	-0.06	0.35	0.41	0.0150	0.0653	0.0539	**0.0194**
52	-0.08	-0.01	-0.07	0.71	**1.07**	-0.36	1.05	0.76	-0.29	0.0353	**0.0047**	0.7848	0.9773
53	-0.31	-0.38	-0.48	**-0.98**	**-1.06**	0.08	-0.89	-0.88	0.01	0.0156	**0.0038**	0.0981	0.6639
54	-0.28	-0.26	0.79	1.08	**1.43**	-0.35	0.37	0.03	-0.34	0.0294	**0.0105**	0.8978	0.1300
55	0.07	-0.27	0.01	-1.41	0.24	-1.65	0.31	-1.68	**-1.99**	0.0131	**0.0015**	0.9906	0.9463
56	0.93	1.00	**0.90**	-1.07	-1.37	0.30	**-1.34**	-0.97	0.37	0.0008	**0.0007**	**0.0019**	0.3392
57	-0.08	-0.11	0.31	1.25	**1.80**	-0.55	1.41	0.83	-0.58	0.0183	**0.0023**	0.9134	0.8856
63	1.20	1.12	0.42	-2.26	0.17	-2.43	0.95	-1.56	**-2.51**	0.0103	**0.0053**	**0.0445**	0.2327
69	0.40	-0.29	-0.09	1.73	2.27	-0.54	**2.77**	1.53	-1.23	0.0155	**0.0023**	0.7273	0.5605
72	0.25	**-1.30**	-0.24	**2.44**	0.42	**2.02**	0.91	1.38	0.47	0.0007	0.0005	0.0403	**0.0168**
76	-0.21	**-0.82**	-0.62	**1.77**	-0.04	**1.81**	0.37	**1.58**	**1.21**	0.0000	0.0001	0.0009	**0.0065**
77	0.28	-1.13	-0.56	-0.77	-0.30	-0.47	0.54	-1.34	**-1.89**	0.0311	**0.0136**	0.2150	0.1847

78	-0.62	**-1.04**	-0.70	**1.06**	-0.14	**1.20**	-0.06	0.72	0.78	0.0018	**0.0025**	**0.0032**	0.1470
79	0.07	**-1.37**	-0.85	-0.14	**-4.46**	**4.32**	**-3.54**	-0.67	2.87	0.0001	**0.0001**	**0.0033**	0.0632
81	-1.16	**-1.08**	0.06	**3.95**	0.57	**3.38**	-0.65	**2.82**	**3.46**	0.0000	0.0001	0.0007	**0.0004**
82	1.03	**-2.03**	$+\infty$	$+\infty$	$+\infty$	**2.23**	**3.92**	3.10	-0.82	0.0000	0.0001	0.0142	**0.0001**
83	0.30	-0.31	-0.40	-0.90	0.53	-1.43	1.22	-0.81	**-2.03**	0.0372	**0.0061**	0.9817	0.5932
84	-0.15	**-1.33**	**-1.07**	0.20	-0.73	**0.93**	0.19	-0.05	-0.25	0.0014	0.1083	0.0003	**0.0249**
85	0.19	**-1.90**	0.13	0.30	-0.08	0.37	-0.01	**-1.73**	**-1.71**	0.0147	0.1881	0.0896	**0.0069**
87	-0.21	**-0.95**	-0.29	0.86	0.23	0.63	0.31	0.20	-0.11	0.0320	0.0783	**0.0215**	0.1387
88	0.02	0.12	0.78	-0.01	1.02	**-1.03**	0.26	-0.67	-0.93	0.0467	**0.0131**	0.2597	0.4342
89	-0.20	-1.12	**-1.14**	-0.23	-0.31	0.08	0.62	-0.22	-0.84	0.0314	0.5506	**0.0033**	0.2120

Group 3:

Pos. No. [a]	Log$_2$(Average Ratio) [b]									One-way ANOVA p Values [c]	Two-way ANOVA p Values [c]		
	W169/W0	B169/B0	A169/A0	B0/A0	W0/A0	B0/W0	W169/A169	B169/A169	B169/W169		Genotype	Nitrogen	Interact
2	-0.05	0.01	0.14	0.51	-0.66	**1.17**	-0.85	0.38	**1.24**	0.0121	**0.0014**	0.8304	0.9291
3	0.93	-0.32	-0.19	0.41	-0.95	**1.36**	0.18	0.29	0.11	0.0120	0.0106	0.7837	**0.0236**
6	-1.17	-0.77	0.22	-0.11	-1.35	1.24	**-2.75**	-1.10	1.64	0.0105	**0.0022**	0.3324	0.3038
10	-0.10	**-0.76**	-0.22	**0.72**	-0.36	**1.07**	-0.24	0.18	0.41	0.0048	**0.0044**	**0.0225**	0.0816
12	-0.52	-0.46	-0.24	-0.13	-0.61	0.48	**-0.88**	-0.35	0.54	0.0263	**0.0117**	**0.0334**	0.8986
16	-0.17	**0.72**	0.30	1.32	**1.62**	-0.30	**1.14**	**1.73**	0.59	0.0003	0.0001	0.0963	**0.0362**
17	-0.44	-0.45	-0.63	1.04	**1.98**	-0.94	**2.17**	1.23	-0.94	0.0018	**0.0003**	0.0991	0.7338
18	0.32	-0.63	0.40	0.56	-0.87	**1.42**	**-0.94**	-0.47	0.47	0.0035	0.0017	0.8521	**0.0242**
20	0.23	-0.48	-0.09	**-0.99**	-0.55	-0.44	-0.24	**-1.38**	**-1.14**	0.0009	**0.0001**	0.6854	0.3199
28	-0.04	-0.16	-0.25	**-0.97**	-0.08	-0.88	0.13	-0.88	**-1.01**	0.0113	**0.0016**	0.3737	0.8081
29	0.21	-0.18	0.09	**1.73**	0.75	**0.97**	0.88	**1.46**	0.58	0.0008	**0.0001**	0.9345	0.5635
30	0.36	0.25	**-0.90**	**-1.39**	-0.80	-0.59	0.47	-0.24	-0.70	0.0102	0.0129	0.3614	**0.0176**
32	0.58	0.39	-0.44	**-1.13**	-0.43	-0.70	0.60	-0.29	**-0.88**	0.0050	**0.0035**	0.3279	0.0228
34	-0.86	0.46	-0.24	0.92	**1.06**	-0.14	0.44	**1.63**	**1.19**	0.0014	0.0006	0.5431	**0.0182**
36	-0.51	**1.91**	1.18	0.25	**2.26**	**-2.01**	0.57	**0.98**	0.41	0.0002	0.0007	0.0163	**0.0009**
40	1.26	-0.74	0.89	**1.72**	-0.10	**1.82**	0.28	0.09	-0.18	0.0139	0.0232	0.4104	**0.0158**
41	0.18	0.75	0.46	0.34	**1.05**	-0.71	0.77	0.64	-0.13	0.0070	**0.0034**	**0.0212**	0.4213
42	0.36	0.56	0.28	0.76	**1.25**	-0.49	**1.33**	**1.04**	-0.29	0.0011	**0.0002**	**0.0250**	0.5367
43	-0.22	**-1.16**	**1.17**	**1.45**	0.27	**1.17**	**-1.12**	-0.88	0.24	0.0015	0.0190	0.5156	**0.0006**
44	**0.88**	0.66	0.24	-0.39	0.08	-0.47	0.71	0.03	-0.68	0.0366	0.0791	**0.0142**	0.2933
45	**1.35**	-0.22	0.28	1.37	0.48	0.89	**1.56**	0.88	-0.67	0.0164	0.0206	0.1040	**0.0463**
46	0.97	**-1.60**	**-1.82**	0.39	-0.92	**1.32**	**1.87**	0.62	-1.25	0.0031	0.3531	0.0143	**0.0014**
48	-0.25	1.07	0.43	-0.37	1.22	**-1.59**	0.54	0.27	-0.27	0.0423	**0.0166**	0.3154	0.2089
49	**0.95**	-0.18	-0.74	**0.93**	-0.23	**1.16**	**1.45**	**1.48**	0.03	0.0001	0.0001	0.6386	**0.0025**
51	-0.27	0.28	0.69	-0.03	**1.40**	**-1.42**	0.44	-0.43	-0.87	0.0022	**0.0004**	0.5599	0.1410
55	0.10	0.64	-0.17	-1.40	0.26	**-1.66**	0.53	-0.59	-1.12	0.0278	**0.0042**	0.6786	0.6709
64	1.05	0.60	**2.52**	0.19	-0.42	0.61	**-1.89**	**-1.72**	0.17	0.0097	0.0404	0.0107	**0.0476**
65	0.11	-0.88	-0.24	-0.94	0.03	-0.96	0.38	**-1.58**	**-1.96**	0.0018	**0.0003**	0.2942	0.3945
66	0.08	0.30	0.19	**0.75**	-0.33	**1.08**	-0.44	**0.86**	**1.30**	0.0002	**0.0001**	0.1332	0.5067
67	0.30	1.35	0.76	-0.14	1.40	**-1.54**	0.94	0.45	-0.49	0.0188	**0.0091**	**0.0307**	0.6348
69	-0.35	0.20	0.84	1.73	**2.29**	-0.56	1.10	1.09	-0.01	0.0174	**0.0030**	0.8998	0.3878
71	-0.55	-0.79	0.48	**1.52**	0.79	0.72	-0.24	0.25	0.48	0.0086	0.0082	0.0762	**0.0489**
72	-1.00	**-1.03**	0.06	**2.45**	0.45	**2.00**	-0.61	1.36	1.98	0.0001	0.0001	0.0131	**0.0317**
73	0.18	**-1.00**	-0.08	0.19	-0.71	**0.91**	-0.46	-0.73	-0.28	0.0244	0.0526	0.0839	**0.0408**
74	-0.10	-0.63	-0.30	**-1.11**	**-1.38**	0.27	**-1.18**	**-1.44**	-0.26	0.0006	**0.0001**	0.1131	0.6400
75	-0.51	**-1.03**	0.05	**0.67**	-0.54	**1.21**	**-1.10**	-0.41	0.69	0.0002	0.0004	0.0027	**0.0055**
76	-0.12	-0.24	-0.69	**1.78**	-0.02	**1.80**	0.54	**2.23**	**1.69**	0.0000	**0.0001**	**0.0184**	0.3455
77	-0.46	-0.41	0.22	-0.76	-0.28	-0.48	-0.96	**-1.40**	-0.43	0.0259	**0.0053**	0.5626	0.3716
78	-0.99	-0.42	-1.00	**1.07**	-0.12	**1.19**	-0.12	**1.65**	**1.77**	0.0002	**0.0001**	**0.0050**	0.9812
90	-1.19	-1.02	0.49	-1.52	0.07	-1.60	**-1.61**	**-3.04**	-1.43	0.0088	**0.0028**	0.4943	0.0988
91	-0.86	**-1.41**	-0.12	0.59	0.55	0.04	-0.19	-0.69	-0.50	0.0116	0.5932	**0.0016**	0.0691
92	-0.48	0.31	-0.45	-0.45	0.97	**-1.43**	0.95	0.31	-0.64	0.0455	**0.0117**	0.3442	0.4174
93	**-1.29**	0.96	0.70	-0.47	0.76	**-1.23**	-1.22	-0.21	1.01	0.0172	0.5221	0.5776	**0.0028**

94	-0.76	1.55	1.10	-1.45	0.77	**-2.21**	-1.09	-0.99	0.09	0.0414	0.0694	0.1932	**0.0475**
95	-0.22	0.25	-0.20	0.98	1.10	-0.13	1.08	**1.42**	0.34	0.0085	**0.0012**	0.9201	0.4481
96	-0.58	-2.44	0.61	0.05	-1.79	1.84	**-2.97**	**-3.00**	-0.03	0.0219	**0.0102**	0.5518	0.0852
97	-0.81	-0.06	-0.03	0.06	-0.19	0.25	**-0.96**	0.04	**1.00**	0.0214	**0.0114**	0.1009	0.1823
98	0.71	0.31	0.43	0.03	0.81	-0.78	**1.08**	-0.09	**-1.18**	0.0107	**0.0044**	**0.0464**	0.3541
99	-0.76	**-1.12**	-0.42	0.51	0.01	0.50	-0.33	-0.20	0.13	0.0389	0.3800	**0.0044**	0.3169
100	-0.39	**2.26**	1.66	-0.93	0.37	-1.30	-1.68	-0.33	1.35	0.0450	0.2922	**0.0177**	0.0928
101	**1.48**	-0.05	**1.50**	0.94	-0.13	1.07	-0.15	-0.62	-0.46	0.0045	0.8195	0.0008	**0.0191**

[a] Position numbers correspond to the numbers on the six representative gels in **Figure S2**. [b] Protein normalized intensities between each treatment and control are expressed as average ratio that are calculated by taking the means of normalized volumes, from three independent biological replicates per treatment, for the corresponding proteins. Values are log transformed and displayed of up-regulated (positive values) or down-regulated (negative values). Boldface for ratios indicating significant fold changes based on Tukey test ($p < 0.05$). [c, d] One-way and two-way analysis of variances (ANOVA) are calculated using SAS software (Version 9.4). Boldface for p values showing main effects (genotype, N and interaction).

Table S2. The identified proteins are listed based on the spectra acquired on the MALDI-TOF/TOF. Homology searches were performed with Matrix Science (www. matrixscience.com) online server with following searched parameters: National Center for Biotechnology Information (NCBInr); *Viridiplantae* (green plants); $p < 0.05$; at least two peptides matched; at most one missed cleavage site; precursor ion mass error tolerance was set as 100 ppm and MS/MS fragment ion mass error tolerance was set as 0.1 Da; peptide charge is +1; monoisotopic and MALDI-TOF/TOF as instrument; fixed modification was carbamidomethyl (Cys) and variable modification was oxidation (Met); and cleavage by trypsin was the C-terminal side of Lys and Arg unless the next residue was Pro.

Spot No.: 81 Representative gel number: A

Protein: glyceraldehyde-3-phosphate dehydrogenase C subunit 1 [*Arabidopsis thaliana*]

Mascot score: 230 Sequence coverage %: 15

NCBI accession No.: gi|15229231

Matched peptides No.: 4

Calculated MW: 37005 Calculated pI: 6.62

Matched peptide sequences: shown in Bold Red

**1**	MADKKIRIGI	NGFGRIGRLV	ARVVLQRDDV	ELVAVNDPFI	TTEYMTYMFK
**51**	**YDSVHGQWK**H	NELKIKDEK**T**	**LLFGEKPVTV**	**FGIR**NPEDIP	WAEAGADYVV
**101**	ESTGVFTDKD	KAAAHLKGGA	KKVVISEPSK	DAPMFVVGVN	EHEYKSDLDI
**151**	VSNASCTTNC	LAPLAKVIND	RFGIVEGLMT	TVHSITATQK	TVDGPSMKDW
**201**	RGGRAASFNI	IPSSTGAAKA	VGKVLPALNG	KLTGMSFR**VP**	**TVDVSVVDLT**
**251**	**VR**LEKAATYE	EIKKAIKEES	EGKLKGILGY	TEDDVVSTDF	VGDNRSSIFD
**301**	AKAGIALSDK	FVK**LVSWYDN**	**EWGYSSR**VVD	LIVHMSKA	

Matched peptides information:

Start	End	Observed	Mr(expt)	Mr(calc)	ppm	Miss	Peptide
51	59	1119.5122	1118.5049	1118.5145	-8.57	0	K.YDSVHGQWK.H
70	84	1676.9735	1675.9662	1675.9661	0.050	0	K.TLLFGEKPVTVFGIR.N
239	252	1498.8425	1497.8352	1497.8403	-3.39	0	R.VPTVDVSVVDLTVR.L
314	327	1761.7810	1760.7737	1760.7794	-3.24	0	K.LVSWYDNEWGYSSR.V

Spot No.: 18   Representative gel number: A

Protein: catalase [*Brassica juncea*]

Mascot score: 211   Sequence coverage %: 11

NCBI accession No.: gi|4336758

Matched peptides No.: 5

Calculated MW: 57252   Calculated pI: 6.63

Matched peptide sequences: shown in Bold Red

**1**	MDPYKYRPAS	SYNSPFFTTN	FGAPVWNNNS	SMTVGPRGPI	LLEDYHLVEK
**51**	LANFDRERIP	ERVVHARGAS	AKGFFEVTHD	ISNLTCADFL	R**APGVQTPVI**
**101**	**VRFSTVIHER**	GSPETLRDPR	GFAVKFYTR**E**	**GNFDLVGNNF**	**PVFFIR**DGMK
**151**	FPDMVHALKP	NPK**SHIQENW**	**R**VLDFFSHHP	ESLNMFTFLF	DDIGIPQDYR
**201**	HMEGSGVNTY	MLINKSGKAH	YVKFHWKPTC	GVKSLLEEDA	IRVGGTNHSH
**251**	ATQDLYDSIA	AGNYPEWKLF	VQIIDPADED	RFDFDPLDVT	K**TWPEDILPL**
**301**	**QPVGR**MVLNK	NIDNFFAENE	QLAFCPAIIV	PGIHYSDDKL	LQTRVFSYAD
**351**	TQRHRLGPNY	LQLPVNAPKC	AHHNNHHEGF	MNFMHRDEEV	NYFPSRYDPV
**401**	RHAEKYPTPP	AICSGKRERC	IIEKENNFKE	PGERYRSFTP	ERQERFIGRW
**451**	IDALSDPRIT	HEIRSIWISY	WSQADKSLGQ	KLANRLNVRP	SI

Matched peptides information:

Start	End	Observed	Mr(expt)	Mr(calc)	ppm	Miss	Peptide
92	102	1136.6705	1135.6632	1135.6714	-7.16	0	R.APGVQTPVIVR.F
103	110	988.5146	987.5073	987.5138	-6.55	0	R.FSTVIHER.G
130	146	1984.9810	1983.9737	1983.9843	-5.34	0	R.EGNFDLVGNNFPVFFIR.D
164	171	1069.5150	1068.5077	1068.5101	-2.20	0	K.SHIQENWR.V
292	305	1620.8621	1619.8548	1619.8671	-7.60	0	K.TWPEDILPLQPVGR.M

Spot No.: 87 Representative gel number: A

Protein: RecName: Full=Aminomethyltransferase, mitochondrial; AltName: Full=Glycine cleavage system T protein; Short=GCVT; Flags: Precursor

Mascot score: 208 Sequence coverage %: 11

NCBI accession No.: gi|3334202

Matched peptides No.: 3

Calculated MW: 44834 Calculated pI: 8.73

Matched peptide sequences: shown in Bold Red

**1**	MRGGGLWQLG	QSVTRRLAQA	EKKVIARRCF	ASEADLKKTA	LYDFHVANGG
**51**	KMVPFAGWSM	PIQYKDSIMD	STINCRENGS	LFDVAHMCGL	SLKGKDCIPF
**101**	LEKLVVGDIA	GLAPGTGTLS	VLTNEKGGAI	DDTVITK**VTD**	**DHIYLVVNAG**
**151**	**CR**EKDLAHIE	EHMKAFKAK**G**	**GDVSWHIHDE**	**RSLLALQGPL**	**AAPVLQHLTK**
**201**	EDLSKFYFGQ	FTFLDINGFP	CYLTRTGYTG	EDGFEISVPN	EYAVDLAKAM
**251**	LEKSEGKVRL	TGRGARDSLR	LEAGLCLYGN	DLEQHITPIE	AGLTWAVGKR
**301**	RRAEGGFLGA	EVILKQIADG	PPQRRVGFIS	SGPPARGHSE	IQNEKGESIG
**351**	EITSGGFSPC	LKKNIAMGYV	KSGNHKAGTK	VNILVRGKPY	EGVVTKMPFV
**401**	PTKYYKSP				

Matched peptides information:

Start	End	Observed	Mr(expt)	Mr(calc)	ppm	Miss	Peptide
138	152	1731.8357	1730.8284	1730.8410	-7.28	0	K.VTDDHIYLVVNAGCR.E
170	181	1407.6245	1406.6172	1406.6327	-11.0	0	K.GGDVSWHIHDER.S
182	200	1970.1707	1969.1634	1969.1724	-4.56	0	R.SLLALQGPLAAPVLQHLTK.E

Spot No.: 46 Representative gel number: A

Protein: chlorophyll a-b binding protein of LHCII type 1-like [*Vigna radiata*]

Mascot score: 122 Sequence coverage %: 14

NCBI accession No.: gi|955078065

Matched peptides No.: 3

Calculated MW: 28003 Calculated pI: 5.14

Matched peptide sequences: shown in Bold Red

**1**	MAASTMALSS	PSLAGQAIKL	SPATPDISGG	RISMRKTASK	**SVSSGSPWYG**
**51**	**PDR**VKYLGPF	SGEAPSYLTG	QFPGDYGWDT	AGLSADPETF	AKNR**ELEVIH**
**101**	**SRWAMLGALG**	**CVFPELLAR**N	GVKFGEAVWF	KAGSQIFSEG	GLDYLGNPSL
**151**	VHAQSILAIW	ATQVILMGAV	EGYRIAGGPL	GEVTDPIYPG	GSFDPLGLAD
**201**	DPEAFAELKV	KELKNGRLAM	FSMFGFFVQA	IVTGKGPLEN	LADHLADPVN
**251**	NNAWAYATNF	VPGK			

Matched peptides information:

Start	End	Observed	Mr(expt)	Mr(calc)	ppm	Miss	Peptide
41	53	1394.6237	1393.6164	1393.6263	-7.05	0	K.SVSSGSPWYGPDR.V
95	102	982.5267	981.5195	981.5243	-4.96	0	R.ELEVIHSR.W
103	119	1919.9669	1918.9596	1918.9797	-10.5	0	R.WAMLGALGCVFPELLAR.N + Oxidation (M)

Spot No.: 5 Representative gel number: A

Protein: heat shock protein 70 [*Lactuca sativa*]

Mascot score: 293 Sequence coverage %: 14

NCBI accession No.: gi|432140649

Matched peptides No.: 6

Calculated MW: 74289 Calculated pI: 5.26

Matched peptide sequences: shown in Bold Red

**1**	MASSTAQIHA	LGGTPFASTQ	NSPKTVFFGK	GLTKSVSFSH	QKSFVKLKKT
**51**	RRNGPLRVVA	EKVVGIDLGI	TNSAVGAMEG	GKPVIVTNAE	GQRTTPSVVA
**101**	YTKNGDRLVG	QIAKRQAVVN	PENTFFSVKR	FIGRKMSEVD	EESKQVSYTV
**151**	VRDENGNVKL	DCPAIGK**QFA**	**AEEISAQVLR**	KLVDDASKFL	NDKVTK**AVVT**
**201**	**VPAYFNDSQR**	TATKDAGRIA	GLEVLR**IINE**	**PTAASLAYGF**	**ER**KNNETILV
**251**	FDLGGGTFDV	SVLEVGDGVF	EVLSTSGDTH	LGGDDFDKRI	VDWLAASFKK
**301**	DEGIDLLKDK	QALQRLTETA	EKAKMELSTL	TQANISLPFI	TATADGPKHI
**351**	DTTLTRAKFE	ELCSDLLDRL	KRPVENSLRD	AKLSFK**DIDE**	**VILVGGSTR**I
**401**	PAVIEVVKSL	TGKEPNVTVN	PDEVVALGAA	VQAGVLAGDV	SDIVLLGVTP
**451**	LSIGLETLGG	VMTKIIPRNT	TLPTSK**SEVF**	**STAADGQTSV**	**EINVLQGER**E
**501**	FVRDNKSLGS	FRLDGIPPAP	RGVPQIEVKF	DIDANGILSV	TAIDKGTGK**K**
**551**	**QDITITGAST**	**LPSDEVER**MV	AEAEKYAKED	KEKREAIDTK	NQAESAVYQC
**601**	EKQLKEVGEK	VPGEVKEKVE	AKLKELKDAI	SGGTTQSMKD	AMAALNQEMM
**651**	QVGQAIYSQG	GGGGAAAAAS	GGEGGKASGS	SGEGDGEVID	ADFSDSQ

Matched peptides information:

Start	End	Observed	Mr(expt)	Mr(calc)	ppm	Miss	Peptide
168	180	1461.7534	1460.7461	1460.7623	-11.1	0	K.QFAAEEISAQVLR.K
197	210	1566.7687	1565.7614	1565.7838	-14.3	0	K.AVVTVPAYFNDSQR.T
227	242	1751.8806	1750.8733	1750.8890	-8.93	0	R.IINEPTAASLAYGFER.K
387	399	1373.7142	1372.7069	1372.7198	-9.39	0	K.DIDEVILVGGSTR.I
477	499	2437.1777	2436.1704	2436.1769	-2.64	0	K.SEVFSTAADGQTSVEINVLQGER.E
550	568	2060.0366	2059.0293	2059.0433	-6.80	1	K.KQDITITGASTLPSDEVER.M

Spot No.: 76 Representative gel number: A

Protein: PREDICTED: glyceraldehyde-3-phosphate dehydrogenase A, chloroplastic [*Eucalyptus grandis*]

Mascot score: 149 Sequence coverage %: 8

NCBI accession No.: gi|702464438

Matched peptides No.: 3

Calculated MW: 43121 Calculated pI: 8.45

Matched peptide sequences: shown in Bold Red

**1**	MASAALSVAK	PSLQANGKGF	AEFSGLRNSA	SVSFGRKNSE	DFLSVIAFQT
**51**	SAVGSNGGYR	KGVVEAKLKV	AINGFGRIGR	NFLRCWHGRK	DSPLDIIAIN
**101**	DTGGIKQASH	LLKYDSTLGI	FEADVKPVGT	DGISVDGKVI	KVVSDRNPLN
**151**	LPWKEMGIDL	VIEGTGVFVD	RDGAGKHIEA	GAKKVLITAP	GKGDIPTYVV
**201**	GVNADLYNPA	EPIISNASCT	TNCLAPFVKV	LDQKFGIIKG	TMTTTHSYTG
**251**	DQR**LLDASHR**	DLRRARAAAL	NIVPTSTGAA	KAVALVLPTL	KGKLNGIALR
**301**	VPTPNVSVVD	LVVQVSKK**TF**	**AEEVNAGFR**E	SADNELK**GIL**	**SVCDEPLVSV**
**351**	**DFR**CSDVSST	VDASLTMVMG	DDMVKVIAWY	DNEWGYSQRV	VDLAHIVADN
**401**	WK				

Matched peptides information:

Start	End	Observed	Mr(expt)	Mr(calc)	ppm	Miss	Peptide
254	260	811.4355	810.4282	810.4348	-8.11	0	R.LLDASHR.D
319	329	1240.5851	1239.5778	1239.5884	-8.53	0	K.TFAEEVNAGFR.E
338	353	1805.8970	1804.8897	1804.9030	-7.33	0	K.GILSVCDEPLVSVDFR.C

Spot No.: 49 Representative gel number: A

Protein: Chaperonin 21, chloroplast [*Cynara cardunculus*]

Mascot score: 99 Sequence coverage %: 17

NCBI accession No.: gi|976914264

Matched peptides No.: 3

Calculated MW: 26314 Calculated pI: 6.02

Matched peptide sequences: shown in Bold Red

**1**	MAAAQLNSTS	VLPSFEGLRS	STSKTSSVVV	SFPFRNLTLR	SARGLVVKSA
**51**	TTVAPK**YTSL**	**KPLGDR**VLVK	IKAAEEK**SIG**	**GILLPSSAQT**	**KPQGGEVVAV**
**101**	**GEGR**TIGEKK	VDIGVKTGTP	VVYSKYAGTE	VEFNGSNHLI	LKEDDIVGIL
**151**	ETDDVK**DLKP**	**LNDR**VLIKVE	EAEETTAGGL	LLTQASKEKP	SIGTVIAVGP
**201**	GPLDEEGNRT	GLPLSPGNTV	LYSKYAGNDF	KGPDGSEYIA	LRSSDVMAVL
**251**	S				

Matched peptides information:

Start	End	Observed	Mr(expt)	Mr(calc)	ppm	Miss	Peptide
57	66	1149.6161	1148.6088	1148.6190	-8.82	0	K.YTSLKPLGDR.V
78	104	2607.3953	2606.3880	2606.4028	-5.66	0	K.SIGGILLPSSAQTKPQGGEVVAVGEGR.T
157	164	970.5404	969.5331	969.5243	9.09	0	K.DLKPLNDR.V

Spot No.: 39 Representative gel number: B

Protein: hypothetical protein AXG93_4542s1060 [*Marchantia polymorpha*]

BlastP Protein: Fructose-bisphosphate aldolase 1 [*Theobroma cacao*]

BlastP GI: gi|590685167

Mascot score: 179 Sequence coverage %: 16

NCBI accession No.: gi|1026765976

Matched peptides No.: 4

Calculated MW: 42947 Calculated pI: 8.18

Matched peptide sequences: shown in Bold Red

**1**	MAMMAVSSGM	ALKSSVCVGK	SDFMGKTALR	QSAAPSSSKT	NVSFTIKAAG
**51**	YDDELVKTAK	SIASPGRGIL	AMDESNATCG	LR**LASIGLEN**	**TEANR**QAYRQ
**101**	LLVQTPGLGE	YISGAILFEE	TLYQSTTDGK	KMVDVLVDQK	IVPGIKVDKG
**151**	LVPLAGSNNE	SWCQGLDGLS	SR**TAAYYKQG**	**AR**FAKWRTVI	SIPNGPSELA
**201**	VQEAAWGLAR	**YAAISQDSGL**	**VPIVEPEILL**	**DGEHGIDR**TL	EIASKVWAAT
**251**	FHYLAQNNVL	FEGILLKPSM	VTPGAGCKER	**ATPEEVAKYT**	**LNLLR**RRVPP
**301**	AVPGIMFLSG	GQSEVEATLN	LNAMNQSPNP	WHVSFSYARA	LQNSVLKTWK
**351**	GQPENVEAAQ	KALLIRAKAN	SLAQLGKYSA	EGEAEEAKKG	MFVEGYTY

Matched peptides information:

Start	End	Observed	Mr(expt)	Mr(calc)	ppm	Miss	Peptide
83	95	1387.7150	1386.7077	1386.7103	-1.83	0	R.LASIGLENTEANR.Q
173	182	1128.5396	1127.5323	1127.5723	-35.5	1	R.TAAYYKQGAR.F
211	238	3006.5420	3005.5347	3005.5346	0.054	0	R.YAAISQDSGLVPIVEPEILLDGEHGIDR.T
281	295	1717.9114	1716.9041	1716.9410	-21.5	1	R.ATPEEVAKYTLNLLR.R

Spot No.: 14 Representative gel number: B

Protein: ATP synthase CF1 alpha subunit protein, chloroplast [*Ilex cornuta*]

Mascot score: 344 Sequence coverage %: 19

NCBI accession No.: gi|290490174

Matched peptides No.: 7

Calculated MW: 55594 Calculated pI: 5.19

Matched peptide sequences: shown in Bold Red

**1**	MVTIRADEIS	NIIRERIEQY	NREVK**IVNTG**	**TVLQVGDGIA**	**R**IHGLDEVMA
**51**	GELVEFEEGT	IGIALNLESN	NVGVVLMGDG	LMIQEGSSVK	ATGK**IAQIPV**
**101**	**SEAYLGR**VIN	ALAKPIDGRG	EISASESR**LI**	**ESPAPGIISR**	R**SVYEPLQTG**
**151**	**LIAIDSMIPI**	**GR**GQRELIIG	DRQTGKTAVA	TDTILNQQGQ	NVICVYVAIG
**201**	QK**ASSVAQVV**	**TTFQER**GAMD	YTIVVAETAD	SPATLQYLAP	YTGASLAEYF
**251**	MYRKEHTLII	YDDPSKQAQA	YRQMSLLLRR	PPGR**EAYPGD**	**VFYLHSR**LLE
**301**	RAAKSSSHLG	EGSMTALPIV	ETQSGDVSAY	IPTNVISITD	GQIFLSADLF
**351**	NSGIRPAINV	GISVSRVGSA	AQIKAMKQVA	GKLKLELAQF	AELEAFAQFS
**401**	SDLDKATQNQ	LARGQRLREL	LKQSQAAPLT	VEEQIMTIYT	GTNGYLDSLE
**451**	IGQVRKFLVE	LRTYLKSNKP	QFQEIISSTK	TFTEEAEALL	K**EAIQEQLER**
**501**	FFMLQEQA				

Matched peptides information:

Start	End	Observed	Mr(expt)	Mr(calc)	ppm	Miss	Peptide
26	41	1612.8903	1611.8830	1611.8944	-7.08	0	K.IVNTGTVLQVGDGIAR.I
95	107	1416.7755	1415.7682	1415.7772	-6.37	0	K.IAQIPVSEAYLGR.V
129	140	1252.7190	1251.7117	1251.7187	-5.54	0	R.LIESPAPGIISR.R
142	162	2289.2146	2288.2073	2288.2086	-0.56	0	R.SVYEPLQTGLIAIDSMIPIGR.G + Oxidation (M)
203	216	1522.7726	1521.7653	1521.7787	-8.81	0	K.ASSVAQVVTTFQER.G
285	297	1553.7267	1552.7194	1552.7310	-7.48	0	R.EAYPGDVFYLHSR.L
492	500	1115.5603	1114.5530	1114.5618	-7.89	0	K.EAIQEQLER.F

Spot No.: 33 Representative gel number: C

Protein: PREDICTED: glutamine synthetase nodule isozyme [*Tarenaya hassleriana*]

Mascot score: 119 Sequence coverage %: 8

NCBI accession No.: gi|729459487

Matched peptides No.: 2

Calculated MW: 39374 Calculated pI: 5.61

Matched peptide sequences: shown in Bold Red

**1**	MSLLSDLINL	NLSDSTEKVI	AEYIWIGGSG	LDMRSKARTL	PGPVTDPSEL
**51**	PKWNYDGSST	GQAPGNDSEV	ILYPQAIFRD	PFRRGNNILV	MCDAYTPAGE
**101**	PIPTNNRHAA	AKIFSHSDVV	AEEPWYGIEQ	EYTLLQKDIK	WPVGWPVGGF
**151**	PGPQGPYYCG	VGADKAFGRD	IVDSHYKACL	YAGISVSGIN	GEVMPGQWEY
**201**	QVGPVVGISA	GDQLWVSRYI	LERITEIAGV	ILSFDPKPIK	GDWNGAGAHT
**251**	NYSTKSMRNE	GGMEIIKKAI	EKLGLR**HKEH**	**ISAYGEGNER**	RLTGK**HETAD**
**301**	**INTFLWGVAN**	**R**GASIRVGRD	TEKEGKGYFE	DRRPASNMDP	YIVTSMIAET
**351**	TILWKP				

Matched peptides information:

Start	End	Observed	Mr(expt)	Mr(calc)	ppm	Miss	Peptide
277	290	1626.7626	1625.7553	1625.7546	0.45	1	R.HKEHISAYGEGNER.R
296	311	1843.9094	1842.9021	1842.9013	0.45	0	K.HETADINTFLWGVANR.G

Spot No.: 32 Representative gel number: C

Protein: Elongation factor, GTP-binding domain-containing protein [*Cynara cardunculus*]

Mascot score: 388 Sequence coverage %: 18

NCBI accession No.: gi|976921619

Matched peptides No.: 6

Calculated MW: 52863 Calculated pI: 6.06

Matched peptide sequences: shown in Bold Red

**1**	MASISAAATA	AASSKPPFSF	ASPSSSSSSS	TPLFTLSKPT	TKLVLSSSFI
**51**	SKSTTNLFLH	TPSSSSAAPS	HRRRSLTVRA	ARGKYERTKP	HVNIGTIGHV
**101**	DHGKTTLTAA	LTMALASTGG	GVAK**KYDEID**	**AAPEER**ARGI	**TINTATVEYE**
**151**	**TENR**HYAHVD	CPGHADYVKN	MITGAAQMDG	AILVVSGADG	PMPQTKEHVL
**201**	LAKQVGVPNM	VVFLNK**QDQV**	**DDEELLELVE**	**LEVR**DLLSSY	EFPGDDIPII
**251**	SGSALLALEA	LTENPKITKG	ENKWVDKIYE	LMAAVDEYIP	IPQR**QTELPF**
**301**	**LCAIEDVFSI**	**TGR**GTVATGR	VERGTVRVGE	SVEIVGLKDT	RTTIVTGVEM
**351**	FQK**ILDEALA**	**GDNVGLLLR**G	IQKIDIQRGM	VLAKPGSITP	HTK**FEALVYV**
**401**	**LK**KEEGGRHS	PFFAGYRPQF	YMRTTDVTGK	VNSIMNDKDE	ESKMVMPGDR
**451**	VKMVVELIMP	VACEQGMRFA	IREGGKTVGA	GVIQSIIE	

Matched peptides information:

Start	End	Observed	Mr(expt)	Mr(calc)	ppm	Miss	Peptide
125	136	1435.6674	1434.6601	1434.6626	-1.76	1	K.KYDEIDAAPEER.A
139	154	1810.8832	1809.8759	1809.8745	0.81	0	R.GITINTATVEYETENR.H
217	234	2171.0703	2170.0630	2170.0641	-0.50	0	K.QDQVDDEELLELVELEVR.D
295	313	2196.0977	2195.0904	2195.0933	-1.29	0	R.QTELPFLCAIEDVFSITGR.G
354	369	1681.9517	1680.9444	1680.9410	2.03	0	K.ILDEALAGDNVGLLLR.G
394	402	1081.5541	1080.5468	1080.6219	-69.5	0	K.FEALVYVLK.K

Spot No.: 20 Representative gel number: C

Protein: RubisCO activase alpha form precursor [*Deschampsia antarctica*]

Mascot score: 308 Sequence coverage %: 12

NCBI accession No.: gi|32481061

Matched peptides No.: 5

Calculated MW: 51381 Calculated pI: 5.96

Matched peptide sequences: shown in Bold Red

**1**	MAAAFSSTVG	APASTPTSFL	GNKLKKQVTS	AVNYHGKSFK	ANRFTVMAKD
**51**	IDEGKQTDGD	KWK**GLAYDIS**	**DDQQDITR**GK	GIVDSLFQAP	MGDGTHEAVL
**101**	SSYEYVSQGL	KKYDFDNTMG	GFYIAPAFMD	KLVVHLSKNF	MTLPNIKIPL
**151**	ILGIWGGKGQ	GKSFQCELVF	AKMGINPIMM	SAGELESGNA	GEPAKLIRQR
**201**	YREAADMIKK	GKMCCLFIND	LDAGAGRMGG	TTQYTVNNQM	VNATLMNIAD
**251**	APTNVQLPGM	YNKEENPRVP	IXVTGNDFST	LYAPLIPDGR	MEK**FYWAPTR**
**301**	EDRIGVCKGI	FQTDNVSDES	VVK**IVDTFPG**	**QSIDFFGALR**	AR**VYDDEVRK**
**351**	WVSSTGIENI	GKRLVNSRDG	PVTFEQPKMT	VEKLLEYGHM	LVQEQDNVKR
**401**	VQLADTYMSQ	AALGDANKDA	MKTGSFYGKG	AQQGTLPVPE	GCTDR**DAKNF**
**451**	**DPTAR**SDDGS	CLYTF			

Matched peptides information:

Start	End	Observed	Mr(expt)	Mr(calc)	ppm	Miss	Peptide
64	78	1709.7980	1708.7907	1708.7904	0.19	0	K.GLAYDISDDQQDITR.G
294	300	940.4675	939.4603	939.4603	-0.029	0	K.FYWAPTR.E
324	340	1882.9674	1881.9601	1881.9625	-1.28	0	K.IVDTFPGQSIDFFGALR.A
343	350	1023.5103	1022.5030	1022.5033	-0.24	1	R.VYDDEVRK.W
446	455	1134.5182	1133.5109	1133.5465	-31.4	1	R.DAKNFDPTAR.S

Spot No.: 62 Representative gel number: C

Protein: carbonic anhydrase 2-like isoform X2 [*Eucalyptus grandis*]

Mascot score: 208 Sequence coverage %: 16

NCBI accession No.: gi|702465377

Matched peptides No.: 3

Calculated MW: 29595 Calculated pI: 6.53

Matched peptide sequences: shown in Bold Red

**1**	MGKDYDEAIE	ALKKLLSEKG	DLKATAAAKV	EQITAELQTA	SPDIKPSSSV
**51**	DRIKTGFTFF	KKEKYDKNPA	LYGELAKGQS	PK**FMVFACSD**	**SRVCPSHVLD**
**101**	**FQPGEAFVVR**	NVANMVPPYD	QTKYAGTGSA	IEYAVLHLKV	QEIVVIGHSA
**151**	CGGIKGLMTF	KYDGPNSTDF	IEDWVKICLP	AKAKVNSQFN	GAALPDLCGH
**201**	CEK**EAVNVSL**	**GNLLTYPFVR**	DGLVNKTLSL	KGGYYDFVNG	AFELWGLDFG
**251**	LSPPLSMKDV	ATILHWKL			

Matched peptides information:

Start	End	Observed	Mr(expt)	Mr(calc)	ppm	Miss	Peptide
83	92	1235.5022	1234.4949	1234.5111	-13.1	0	K.FMVFACSDSR.V + Oxidation (M)
93	110	2057.0195	2056.0122	2056.0201	-3.81	0	R.VCPSHVLDFQPGEAFVVR.N
204	220	1892.0178	1891.0105	1891.0203	-5.19	0	K.EAVNVSLGNLLTYPFVR.D

Spot No.: 77 Representative gel number: D

Protein: glyceraldehyde-3-phosphate dehydrogenase GAPC1, cytosolic-like [*Tarenaya hassleriana*]

Mascot score: 406 Sequence coverage %: 22

NCBI accession No.: gi|729304835

Matched peptides No.: 5

Calculated MW: 36769 Calculated pI: 7.06

Matched peptide sequences: shown in Bold Red

**1**	MADKKIRIGI	NGFGRIGRLV	ARVVLQRSDV	ELVAVNDPFI	TTEYMTYMFK
**51**	**YDSVHGQWK**H	HELKVKDDK**T**	**LLFGEKPVTV**	**FGIR**NPEDIP	WGEAGADFVV
**101**	ESTGVFTDKD	KAAAHLKGGA	KKVVISAPSK	DAPMFVVGVN	EHEYKSDLNI
**151**	VSNASCTTNC	LAPLAKVIND	R**FGIVEGLMT**	**TVHSITATQK**	TVDGPSMKDW
**201**	RGGRAASFNI	IPSSTGAAKA	VGKVLPELNG	KLTGMSFR**VP**	**TVDVSVVDLT**
**251**	**VR**LEKAATYD	EIKKAIKEES	EGKLK**GILGY**	**TEDDVVSTDF**	**VGDSR**SSIFD
**301**	AKAGIALSDN	FVKLVSWYDN	EWGYSTRVVD	LIVHMSKA	

Matched peptides information:

Start	End	Observed	Mr(expt)	Mr(calc)	ppm	Miss	Peptide
51	59	1119.5277	1118.5204	1118.5145	5.29	0	K.YDSVHGQWK.H
70	84	1676.9814	1675.9741	1675.9661	4.76	0	K.TLLFGEKPVTVFGIR.N
172	190	2049.0737	2048.0664	2048.0612	2.53	0	R.FGIVEGLMTTVHSITATQK.T + Oxidation (M)
239	252	1498.8535	1497.8462	1497.8403	3.95	0	R.VPTVDVSVVDLTVR.L
276	295	2145.0090	2144.0017	2143.9910	5.00	0	K.GILGYTEDDVVSTDFVGDSR.S

Spot No.: 15 Representative gel number: D

Protein: ATP synthase CF1 alpha subunit, partial, chloroplast [*Viburnum acerifolium*]

Mascot score: 453 Sequence coverage %: 22

NCBI accession No.: gi|669218688

Matched peptides No.: 9

Calculated MW: 55648 Calculated pI: 5.21

Matched peptide sequences: shown in Bold Red

**1**	MVTIRADEIS	NIIRERIEQY	NREVK**IVNTG**	**TVLQVGDGIA**	**R**IHGLDEVMA
**51**	GELVEFEEGT	IGIALNLESN	NVGVVLMGDG	LMIQEGSSVR	ATGR**IAQIPV**
**101**	**SEAYLGR**VVN	ALAKPIDGRG	EISASEYR**LI**	**ESPAPGIISR**	R**SVYEPLQTG**
**151**	**LIAIDSMIPI**	**GR**GQRELIIG	DRQTGKTAVA	TDTILNQQGQ	NVICVYVAIG
**201**	QK**ASSVAQVV**	**TTFQER**GAME	YTIVVAETAD	SPATLQYLAP	YTGAALAEYF
**251**	MYRERHTSII	YDDLSKQAQA	YRQMSLLLRR	PPGR**EAYPGD**	**VFYLHSR**LLE
**301**	RAAKLSSRLG	EGSMTALPIV	ETQSGDVSAY	IPTNVISITD	GQIFLSADLF
**351**	NAGIRPAINV	GISVSRVGSA	AQIKAMKQVA	GKLKLELAQF	AELEAFAQFS
**401**	SDLDKATQNQ	LARGQRLREL	LKQSQAAPLT	VEEQIMTIYT	GTNGYLDSLE
**451**	IVQVRKFLVE	LRTYLK**TNKP**	**QFQEIISSTK**	TFTEEAEALL	K**EAIQEQLER**

Matched peptides information:

Start	End	Observed	Mr(expt)	Mr(calc)	ppm	Miss	Peptide
26	41	1612.8982	1611.8909	1611.8944	-2.18	0	K.IVNTGTVLQVGDGIAR.I
95	107	1416.7802	1415.7729	1415.7772	-3.05	0	R.IAQIPVSEAYLGR.V
129	140	1252.7271	1251.7198	1251.7187	0.93	0	R.LIESPAPGIISR.R
142	162	2273.2300	2272.2227	2272.2137	3.98	0	R.SVYEPLQTGLIAIDSMIPIGR.G
142	162	2289.2153	2288.2080	2288.2086	-0.25	0	R.SVYEPLQTGLIAIDSMIPIGR.G + Oxidation (M)
203	216	1522.7872	1521.7799	1521.7787	0.78	0	K.ASSVAQVVTTFQER.G
285	297	1553.7362	1552.7289	1552.7310	-1.36	0	R.EAYPGDVFYLHSR.L
467	480	1620.8452	1619.8379	1619.8519	-8.61	0	K.TNKPQFQEIISSTK.T
492	500	1115.5646	1114.5573	1114.5618	-4.03	0	K.EAIQEQLER.F

Table S3. The identified proteins are listed based on the spectra accquired on the LC-LTQ Orbitrap. The MS/MS spectra were generated and searched against the Viridiplantae dabase from the National Center for Biotechnology Information (November, 2013) using the Sorcerer SEQUEST-based search algorithm (version 2.0, SAGE-N). Searching parameters included maximum peptide ion mass tolerance is 50 ppm, and with methionine oxidation (M)and carbamidomethylation of cysteine (C), and allowed up to 2 missed cleavages. The scoffard4 program were used to verify identities. For each identification, a minimum of two peptides; peptide threshold (p < 0.05) and protein threshold (p < 0.001); Xcorr 2.0, 3.0 and above for the charge states and +2, +3 and above respectively, and minimum Delta CN (Delta correlation) of 0.1.

					LC-MS/MS									
Pos.	Pro.	Accession Num.	Num. Mathced Pep.	Sequence Coverage	Sequence	P(Pep.)	SEQUEST XCorr	SEQUEST deltaCn	Modifications	Observed	Actual Mass	Charge	Delta Da	Delta PPM
1	hypothetical protein C	gi|557543325	16	15%										
					(K)STLTDSLVAAAC	100%	5.69	0.35		1129.1047	2,256.19	2	-0.00145	-0.64
					(K)STLTDSLVAAAC	100%	5.04	0.28		753.0708	2,256.19	3	-0.00580	-2.57
					(R)GFVQFcYEPIK(Q	100%	3.01	0.37	Carbamidomethyl (+57)	694.3373	1,386.66	2	-0.00421	-3.03
					(R)NcDPEGPLmLYV	100%	3.68	0.38	Carbamidomethyl (+57), Oxidation (+16)	819.8778	1,637.74	2	-0.00209	-1.27
					(R)NcDPEGPLMLYV	100%	3.57	0.39	Carbamidomethyl (+57)	811.8810	1,621.75	2	-0.00070	-0.43
					(K)NATLTNEKEVDA	99%	2.61	0.21		904.4684	1,806.92	2	-0.00023	-0.13
					(K)NATLTNEKEVDA	99%	2.61	0.21		904.4684	1,806.92	2	-0.00023	-0.13
					(K)DLQDDFmGGAE	100%	4.47	0.46	Oxidation (+16)	784.3691	1,566.72	2	-0.00026	-0.16
					(R)GGGQVIPTAR(R	98%	2.22	0.22		478.2685	954.52	2	-0.00244	-2.55
					(R)RVIYASQLTAKP	100%	4.17	0.34		501.6303	1,501.87	3	-0.00387	-2.57
					(R)RVIYASQLTAKP	100%	4.17	0.34		501.6303	1,501.87	3	-0.00387	-2.57
					(R)VIYASQLTAKPR	100%	2.97	0.44		673.8920	1,345.77	2	-0.00249	-1.85
					(R)VIYASQLTAKPR	100%	2.97	0.44		673.8920	1,345.77	2	-0.00249	-1.85
					(K)AYLPVVESFGFS	100%	4.02	0.47		886.9644	1,771.91	2	-0.00019	-0.11
					(K)EQmTPLSEYEDK	100%	2.30	0.31	Oxidation (+16)	743.3231	1,484.63	2	-0.00260	-1.75
					(K)EQmTPLSEYEDK	100%	2.96	0.34	Oxidation (+16)	799.8660	1,597.72	2	-0.00088	-0.55
100	hypothetical protein E	gi|557101657	7	26%										
					(K)KYEPTIGVEVHP	99%	3.12	0.14	Carbamidomethyl (+57)	817.7398	2,450.20	3	0.00326	1.33
					(R)FYcWDTAGQEK(	100%	3.09	0.41	Carbamidomethyl (+57)	702.7970	1,403.58	2	-0.00229	-1.63
					(K)KNLQYYEISAK(	100%	2.96	0.38		678.8601	1,355.71	2	-0.00291	-2.14
					(K)KNLQYYEISAK(	100%	2.96	0.38		678.8601	1,355.71	2	-0.00291	-2.14
					(K)NLQYYEISAK(S)	100%	2.85	0.30		614.8130	1,227.61	2	-0.00215	-1.75
					(K)NLQYYEISAK(S)	100%	2.85	0.30		614.8130	1,227.61	2	-0.00215	-1.75
					(K)SNYNFEKPFLYL	100%	2.73	0.23		881.4532	1,760.89	2	0.00337	1.91
17	Mitochondrial lipoami	gi|508714937	9	12%										
					(R)GIEGLFK(K)	98%	2.19	0.17		382.2205	762.43	2	-0.00128	-1.68
					(K)NIIIATGSDVK(S)	100%	3.13	0.29		565.8225	1,129.63	2	-0.00400	-3.54
					(K)NIIIATGSDVK(S)	100%	2.63	0.19		565.8226	1,129.63	2	-0.00388	-3.43
					(K)NIIIATGSDVK(S)	100%	2.71	0.17		565.8240	1,129.63	2	-0.00095	-0.84
					(K)SLPGITIDEK(R)	100%	2.32	0.38		536.7961	1,071.58	2	-0.00386	-3.60
					(K)SLPGITIDEK(R)	100%	2.32	0.38		536.7961	1,071.58	2	-0.00386	-3.60
					(R)LGSEVTVVEFAP	100%	4.59	0.13		863.4466	2,587.32	3	-0.00272	-1.05
					(K)AIDDAEGIVK(I)	100%	3.58	0.14		515.7724	1,029.53	2	-0.00422	-4.10
					(K)AIDDAEGIVK(I)	100%	3.58	0.14		515.7724	1,029.53	2	-0.00422	-4.10
19	hypothetical protein E	gi|557113323	14	20%										
					(R)APGVQTPVIVR(I	100%	3.16	0.11		568.8413	1,135.67	2	-0.00341	-3.00
					(R)APGVQTPVIVR(I	97%	2.49	0.13		568.8404	1,135.67	2	-0.00524	-4.61
					(R)FSTVIHER(G)	100%	2.45	0.22		494.7631	987.51	2	-0.00214	-2.16
					(K)FPDMVHALKPN	100%	3.22	0.37		747.4009	1,492.79	2	0.00094	0.63
					(K)FPDmVHALKPN	100%	3.16	0.26	Oxidation (+16)	755.3963	1,508.78	2	-0.00314	-2.08
					(K)SHIQENWR(V)	100%	2.51	0.27		535.2606	1,068.51	2	-0.00350	-3.28
					(K)SHIQENWR(V)	100%	2.51	0.27		535.2606	1,068.51	2	-0.00350	-3.28
					(K)FHWKPTcGVK(S	95%	2.13	0.16	Carbamidomethyl (+57)	630.3174	1,258.62	2	-0.00802	-6.37
					(Q)DLYDSIAAGNYI	100%	3.23	0.46		871.4083	1,740.80	2	0.00243	1.39
					(R)LGPNYLQLPVNA	100%	4.24	0.45		762.4307	1,522.85	2	-0.00419	-2.75
					(R)LGPNYLQLPVNA	100%	4.24	0.45		762.4307	1,522.85	2	-0.00419	-2.75
					(R)DEEVNYFPSR(Y	100%	2.97	0.18		628.2792	1,254.54	2	-0.00775	-6.17
					(R)LNVRPSI(-)	100%	2.16	0.23		399.7454	797.48	2	0.00032	0.40
					(R)LNVRPSI(-)	100%	2.19	0.24		399.7452	797.48	2	-0.00011	-0.13
22	hypothetical protein C	gi|557528577	8	14%										
					(R)GIYAYGFEKPSA	100%	3.47	0.45		914.4744	1,826.93	2	0.00263	1.44
					(R)GIYAYGFEKPSA	100%	3.47	0.45		914.4744	1,826.93	2	0.00263	1.44
					(R)GIYAYGFEKPSA	100%	3.17	0.20		609.9830	1,826.93	3	-0.00459	-2.51
					(R)GIYAYGFEKPSA	100%	3.17	0.20		609.9830	1,826.93	3	-0.00459	-2.51
					(K)GLDVIQQAQSGT	100%	3.97	0.44		701.3697	1,400.72	2	-0.00144	-1.03
					(K)GLDVIQQAQSGT	100%	3.97	0.44		701.3697	1,400.72	2	-0.00144	-1.03
					(K)VQVGVFSATmPI	100%	3.62	0.31	Oxidation (+16)	1031.0435	2,060.07	2	0.01098	5.33
					(R)VLITTDLLAR(G)	100%	2.68	0.32		557.8434	1,113.67	2	-0.00376	-3.37
25	hypothetical protein E	gi|557091672	8	24%										
					(R)ISITGAGGFIASH	100%	3.75	0.46		785.9363	1,569.86	2	-0.00486	-3.10
					(R)ISITGAGGFIASH	100%	3.75	0.46		785.9363	1,569.86	2	-0.00486	-3.10
					(R)FFYASSAcIYPEF	100%	4.40	0.47	Carbamidomethyl (+57)	865.3992	1,728.78	2	-0.00183	-1.06
					(K)ESDAWPAEPQDA	100%	3.03	0.27		953.4288	1,904.84	2	0.00004	0.02
					(K)DFGIEcR(I)	100%	2.31	0.21	Carbamidomethyl (+57)	448.6990	895.38	2	-0.00242	-2.70
					(R)FHNIYGPFGTWK	96%	2.13	0.19		733.8626	1,465.71	2	-0.00370	-2.53
					(R)FEMWGDGLQTR	99%	2.10	0.20		670.3068	1,338.60	2	-0.00390	-2.91
					(R)SFTFIDEcVEGVL	100%	2.48	0.37	Carbamidomethyl (+57)	836.4059	1,670.80	2	-0.00032	-0.19
36	hypothetical protein C	gi|557552355	4	13%										
					(K)SAEVVNLVTQEI	100%	4.16	0.23		772.4193	1,542.82	2	-0.00140	-0.91
					(K)SAEVVNLVTQEI	100%	4.16	0.23		772.4193	1,542.82	2	-0.00140	-0.91
					(K)ITSFLDPDGWK(	100%	2.41	0.29		639.8204	1,277.63	2	-0.00328	-2.56
					(K)TVLVDNEDFLK(	99%	2.31	0.23		646.8392	1,291.66	2	-0.00238	-1.84
37	RecName: Full=Photo	gi|75252730	26	22%										
					(R)SIPSAEDEDFNYI	100%	3.60	0.44		771.8377	1,541.66	2	-0.00261	-1.69
					(R)SIPSAEDEDFNYI	100%	2.72	0.47		771.8360	1,541.66	2	-0.00603	-3.91
					(R)SIPSAEDEDFNYI	100%	2.48	0.41		771.8365	1,541.66	2	-0.00493	-3.19

					(R)SIPSAEDEDFNYI	95%	2.07	0.20		771.8382	1,541.66	2	-0.00163	-1.06
					(Q)SAEmVTDEGAIY	100%	3.70	0.37	Oxidation (+16)	929.9274	1,857.84	2	-0.00118	-0.63
					(Q)SAEmVTDEGAIY	100%	3.78	0.35	Oxidation (+16)	929.9291	1,857.84	2	0.00212	1.14
					(Q)SAEMVTDEGAIY	100%	3.83	0.28		614.9540	1,841.84	3	-0.00646	-3.51
					(Q)SAEmVTDEGAIY	100%	4.46	0.29	Oxidation (+16)	929.9268	1,857.84	2	-0.00240	-1.29
					(Q)SAEmVTDEGAIY	100%	3.66	0.36	Oxidation (+16)	929.9275	1,857.84	2	-0.00106	-0.57
					(Q)SAEMVTDEGAIY	100%	3.82	0.33		921.9318	1,841.85	2	0.00253	1.37
					(Q)SAEMVTDEGAIY	100%	3.58	0.31		921.9312	1,841.85	2	0.00118	0.64
					(Q)SAEmVTDEGAIY	100%	3.71	0.21	Oxidation (+16)	620.2841	1,857.83	3	-0.01114	-5.99
					(K)AAVQETVSATLI	100%	3.77	0.17		680.3629	1,358.71	2	-0.00421	-3.10
					(K)AAVQETVSATLI	100%	3.83	0.15		680.3629	1,358.71	2	-0.00421	-3.10
					(K)AAVQETVSATLI	100%	3.34	0.12		680.3638	1,358.71	2	-0.00226	-1.66
					(K)AAVQETVSATLI	95%	2.53	0.13		680.3626	1,358.71	2	-0.00470	-3.46
					(R)TVSSGISGASYY	100%	6.12	0.57		1091.5239	2,181.03	2	-0.00047	-0.22
					(R)TVSSGISGASYY	100%	4.90	0.58		1091.5265	2,181.04	2	0.00466	2.13
					(R)TVSSGISGASYY	100%	4.81	0.55		1091.5243	2,181.03	2	0.00026	0.12
					(R)TVSSGISGASYY	100%	4.61	0.52		1091.5242	2,181.03	2	0.00002	0.01
					(R)TVSSGISGASYY	100%	4.31	0.42		728.0158	2,181.03	3	-0.00818	-3.75
					(R)RIQNMGWR(A)	99%	2.60	0.13		530.7753	1,059.54	2	-0.00356	-3.36
					(R)IQNMGWR(A)	100%	2.65	0.14		452.7248	903.44	2	-0.00360	-3.98
					(R)IQNmGWR(A)	99%	2.16	0.17	Oxidation (+16)	460.7231	919.43	2	-0.00193	-2.10
					(R)ADGGLWLLVR((	100%	3.69	0.29		550.3146	1,098.61	2	-0.00405	-3.68
					(R)GFGILDVGYR(S)	100%	3.31	0.36		548.7906	1,095.57	2	-0.00485	-4.42
40	RecName: Full=Photo	gi|75252730	4	12%										
					(Q)SAEMVTDEGAIY	100%	3.25	0.37		921.9338	1,841.85	2	0.00655	3.56
					(Q)SAEmVTDEGAIY	100%	2.96	0.30	Oxidation (+16)	929.9296	1,857.84	2	0.00322	1.73
					(K)AAVQETVSATLI	100%	3.28	0.17		680.3648	1,358.72	2	-0.00031	-0.23
					(R)TVSSGISGASYY	100%	2.57	0.23		1091.5283	2,181.04	2	0.00832	3.81
41	RecName: Full=Oxyg	gi|12644171	3	13%										
					(R)LTYDEIQSK(T)	99%	2.66	0.16		548.7779	1,095.54	2	-0.00374	-3.41
					(K)DGIDYAAVTVQI	100%	3.34	0.37		880.9467	1,759.88	2	0.00451	2.56
					(R)GGSTGYDNAVA	100%	3.59	0.41		781.8802	1,561.75	2	-0.00272	-1.74
45	hypothetical protein C	gi|557548431	3	14%										
					(K)cPDTNYLFmGDY	100%	2.66	0.44	Carbamidomethyl (+57), Oxidation (+16)	941.3982	1,880.78	2	0.01068	5.68
					(R)AHQLVmEGYNW	100%	3.53	0.38	Oxidation (+16)	648.2989	1,941.87	3	-0.00445	-2.29
					(K)VVTIFSAPNYcYI	100%	2.77	0.24	Carbamidomethyl (+57)	795.3916	1,588.77	2	-0.00208	-1.31
47	PREDICTED: chlorop	gi|225436257	9	14%										
					(R)RFQDWANPGSm	100%	3.71	0.42	Oxidation (+16)	755.3474	1,508.68	2	-0.00304	-2.01
					(R)RFQDWANPGSM	100%	3.25	0.38		747.3506	1,492.69	2	-0.00165	-1.11
					(R)FQDWANPGSMC	100%	3.72	0.43		669.3001	1,336.59	2	-0.00150	-1.12
					(R)FQDWANPGSmG	100%	3.73	0.40	Oxidation (+16)	677.2955	1,352.58	2	-0.00557	-4.12
					(R)FQDWANPGSmG	100%	3.60	0.40	Oxidation (+16)	677.2966	1,352.58	2	-0.00350	-2.58
					(R)FQDWANPGSmG	100%	3.31	0.36	Oxidation (+16)	677.2960	1,352.58	2	-0.00472	-3.49
					(K)YLGGSGDPAYP(	100%	4.11	0.53		1141.0681	2,280.12	2	-0.00029	-0.13
					(K)YLGGSGDPAYP(	100%	4.97	0.47		885.1031	2,652.29	3	0.00087	0.33
					(K)YLGGSGDPAYP(	100%	3.48	0.52		1327.1498	2,652.29	2	-0.00156	-0.59
51	Triosephosphate isom	gi|508705864	3	10%										
					(K)TFDVcFQQLK(A	100%	2.37	0.27	Carbamidomethyl (+57)	643.3162	1,284.62	2	0.00046	0.36
					(K)EEDIDGFLVGGA	100%	3.33	0.18		775.3874	1,548.76	2	-0.00724	-4.67
					(K)GPEFATIINSVTS	100%	3.78	0.46		732.3890	1,462.76	2	-0.00347	-2.37
57	RecName: Full=Oxyg	gi|12644171	7	17%										
					(K)RLTYDEIQSK(T)	100%	3.08	0.21		418.2227	1,251.65	3	0.00016	0.13
					(R)LTYDEIQSK(T)	99%	2.58	0.17		548.7783	1,095.54	2	-0.00288	-2.63
					(K)KFcLEPTSFTVK(	100%	4.07	0.28	Carbamidomethyl (+57)	728.8771	1,455.74	2	-0.00362	-2.48
					(K)FcLEPTSFTVK(A	100%	2.83	0.21	Carbamidomethyl (+57)	664.8295	1,327.64	2	-0.00383	-2.88
					(K)DGIDYAAVTVQI	99%	2.54	0.23		880.9461	1,759.88	2	0.00329	1.87
					(R)GGSTGYDNAVA	100%	3.04	0.30		781.8807	1,561.75	2	-0.00187	-1.20
					(R)GGSTGYDNAVA	100%	2.72	0.30		781.8801	1,561.75	2	-0.00297	-1.90
58	RecName: Full=Oxyg	gi|12644171	6	17%										
					(K)RLTYDEIQSK(T)	100%	3.50	0.15		418.2223	1,251.65	3	-0.00094	-0.75
					(R)LTYDEIQSK(T)	100%	2.68	0.25		548.7777	1,095.54	2	-0.00410	-3.74
					(K)KFcLEPTSFTVK(	100%	3.21	0.26	Carbamidomethyl (+57)	728.8779	1,455.74	2	-0.00203	-1.39
					(K)FcLEPTSFTVK(A	100%	2.79	0.19	Carbamidomethyl (+57)	664.8290	1,327.64	2	-0.00493	-3.71
					(K)DGIDYAAVTVQI	100%	3.07	0.29		880.9484	1,759.88	2	0.00793	4.50
					(R)GGSTGYDNAVA	100%	3.17	0.20		781.8798	1,561.75	2	-0.00358	-2.29
6	PREDICTED: phosph	gi|225424316	4	11%										
					(K)ASGAFILTASHN	100%	4.41	0.30		767.3805	2,299.12	3	-0.00392	-1.70
					(K)SIFDFQSIQK(L)	100%	2.66	0.19		606.8151	1,211.62	2	-0.00320	-2.64
					(R)IFVEELGAQESSI	100%	6.39	0.47	Carbamidomethyl (+57)	1067.0494	2,132.08	2	0.00179	0.84
					(R)YDYENVDAGAA	100%	2.83	0.51		658.2910	1,314.57	2	-0.00551	-4.19
63	PREDICTED: outer p	gi|502090241	11	9%										
					(K)KGELFLADVNT(	100%	4.90	0.37		788.4399	1,574.87	2	-0.00174	-1.10
					(K)KGELFLADVNT(	100%	4.90	0.37		788.4399	1,574.87	2	-0.00174	-1.10
					(K)KGELFLADVNT(	100%	4.29	0.20		525.9615	1,574.86	3	-0.00444	-2.82
					(K)KGELFLADVNT(	100%	4.29	0.20		525.9615	1,574.86	3	-0.00444	-2.82
					(K)KGELFLADVNT(	100%	2.61	0.28		788.4395	1,574.86	2	-0.00272	-1.72
					(K)KGELFLADVNT(	100%	2.61	0.28		788.4395	1,574.86	2	-0.00272	-1.72
					(K)GELFLADVNTQI	100%	3.81	0.25		724.3915	1,446.77	2	-0.00366	-2.53
					(K)GELFLADVNTQI	100%	3.81	0.25		724.3915	1,446.77	2	-0.00366	-2.53
					(K)SLFTISGEVDTK(	100%	3.20	0.14		648.8364	1,295.66	2	-0.00277	-2.14
					(K)SLFTISGEVDTK(	98%	2.76	0.12		648.8358	1,295.66	2	-0.00411	-3.17
					(K)SLFTISGEVDTK(	97%	2.75	0.16		648.8377	1,295.66	2	-0.00021	-0.16

96	PREDICTED: triosepl	gi|225449541	2	11%										
					(R)ESGSTMEVVAA(	97%	2.92	0.10		719.3463	1,436.68	2	-0.00370	-2.57
					(K)VATPAQAQEVH	100%	2.55	0.37		818.4238	1,634.83	2	-0.00469	-2.87
101	hypothetical protein E	gi|557092174	3	9%										
					(K)GTMTTTHSYTGI	98%	2.40	0.18		778.3405	1,554.67	2	-0.00699	-4.50
					(K)AVALVLPNLK(C	98%	2.60	0.16		519.3380	1,036.66	2	-0.00322	-3.10
					(R)VVDLADIVANN	100%	3.65	0.31		728.8916	1,455.77	2	-0.00362	-2.48
11	hypothetical protein Z	gi|413943084	13	22%										
					(K)LELQEVVDFLK(	100%	3.81	0.35		666.8718	1,331.73	2	-0.00477	-3.58
					(K)GcLLVGPPGTGK	100%	2.96	0.29	Carbamidomethyl (+57)	578.3112	1,154.61	2	-0.00425	-3.68
					(K)APcIVFIDEIDAV	100%	5.12	0.35	Carbamidomethyl (+57)	837.9275	1,673.84	2	-0.00451	-2.69
					(R)TPGFTGADLQNI	100%	4.05	0.33	Oxidation (+16)	1145.5796	2,289.14	2	0.00204	0.89
					(R)TPGFTGADLQNI	100%	3.78	0.11	Oxidation (+16)	764.0533	2,289.14	3	-0.00456	-1.99
					(R)LVAYHEAGHAL	100%	4.90	0.29	Oxidation (+16)	856.4362	2,566.29	3	-0.00254	-0.99
					(R)LVAYHEAGHAL	100%	4.90	0.29	Oxidation (+16)	856.4362	2,566.29	3	-0.00254	-0.99
					(R)GQAGGLTFFAPS	100%	3.27	0.49		783.8770	1,565.74	2	-0.00809	-5.17
					(R)SYLENQMAVAL	100%	4.07	0.41		754.8779	1,507.74	2	-0.00419	-2.77
					(R)SYLENQmAVAL(	100%	2.73	0.19	Oxidation (+16)	762.8778	1,523.74	2	0.00078	0.51
					(R)VAEEVIFGQDNV	100%	4.31	0.17	Oxidation (+16)	1365.6346	2,729.25	2	-0.00565	-2.07
					(K)DYSmATADVVD	100%	3.93	0.51	Oxidation (+16)	829.3700	1,656.73	2	-0.00475	-2.86
					(K)DYSMATADVVD	100%	3.01	0.38		821.3732	1,640.73	2	-0.00336	-2.05
13	ATP synthase CF1 alp	gi|545718810	47	18%										
					(R)ADEISNIIR(E)	100%	3.25	0.18		515.7784	1,029.54	2	-0.00324	-3.14
					(R)ADEISNIIR(E)	100%	3.25	0.18		515.7784	1,029.54	2	-0.00324	-3.14
					(R)ADEISNIIR(E)	100%	3.25	0.18		515.7784	1,029.54	2	-0.00324	-3.14
					(R)ADEISNIIR(E)	100%	3.05	0.13		515.7783	1,029.54	2	-0.00348	-3.38
					(R)ADEISNIIR(E)	100%	3.05	0.13		515.7783	1,029.54	2	-0.00348	-3.38
					(R)ADEISNIIR(E)	100%	3.05	0.13		515.7783	1,029.54	2	-0.00348	-3.38
					(R)ADEISNIIR(E)	99%	2.81	0.10		515.7778	1,029.54	2	-0.00446	-4.33
					(R)ADEISNIIR(E)	99%	2.81	0.10		515.7778	1,029.54	2	-0.00446	-4.33
					(R)ADEISNIIR(E)	99%	2.81	0.10		515.7778	1,029.54	2	-0.00446	-4.33
					(R)IAQIPVSEAYLGI	100%	3.12	0.14		708.8938	1,415.77	2	-0.00433	-3.05
					(R)IAQIPVSEAYLGI	100%	3.12	0.14		708.8938	1,415.77	2	-0.00433	-3.05
					(R)IAQIPVSEAYLGI	100%	3.12	0.14		708.8938	1,415.77	2	-0.00433	-3.05
					(R)IAQIPVSEAYLGI	100%	2.90	0.13		708.8932	1,415.77	2	-0.00542	-3.83
					(R)IAQIPVSEAYLGI	100%	2.90	0.13		708.8932	1,415.77	2	-0.00542	-3.83
					(R)IAQIPVSEAYLGI	100%	2.90	0.13		708.8932	1,415.77	2	-0.00542	-3.83
					(R)LIESPAPGIISR(R	100%	3.09	0.39		626.8639	1,251.71	2	-0.00564	-4.50
					(R)LIESPAPGIISR(R	100%	3.09	0.39		626.8639	1,251.71	2	-0.00564	-4.50
					(R)LIESPAPGIISR(R	100%	3.09	0.39		626.8639	1,251.71	2	-0.00564	-4.50
					(R)LIESPAPGIISR(R	100%	2.81	0.35		626.8643	1,251.71	2	-0.00491	-3.92
					(R)LIESPAPGIISR(R	100%	2.81	0.35		626.8643	1,251.71	2	-0.00491	-3.92
					(R)LIESPAPGIISR(R	100%	2.81	0.35		626.8643	1,251.71	2	-0.00491	-3.92
					(R)LIESPAPGIISR(R	100%	2.61	0.32		626.8638	1,251.71	2	-0.00576	-4.60
					(R)LIESPAPGIISR(R	100%	2.61	0.32		626.8638	1,251.71	2	-0.00576	-4.60
					(R)LIESPAPGIISR(R	100%	2.61	0.32		626.8638	1,251.71	2	-0.00576	-4.60
					(R)LIESPAPGIISR(R	100%	2.15	0.31		626.8653	1,251.72	2	-0.00283	-2.26
					(R)LIESPAPGIISR(R	100%	2.15	0.31		626.8653	1,251.72	2	-0.00283	-2.26
					(R)LIESPAPGIISR(R	100%	2.15	0.31		626.8653	1,251.72	2	-0.00283	-2.26
					(R)LIESPAPGIISR(R	100%	2.51	0.23		626.8646	1,251.71	2	-0.00430	-3.43
					(R)LIESPAPGIISR(R	100%	2.51	0.23		626.8646	1,251.71	2	-0.00430	-3.43
					(R)LIESPAPGIISR(R	100%	2.51	0.23		626.8646	1,251.71	2	-0.00430	-3.43
					(R)LIESPAPGIISR(R	99%	2.18	0.20		626.8637	1,251.71	2	-0.00601	-4.79
					(R)LIESPAPGIISR(R	99%	2.18	0.20		626.8637	1,251.71	2	-0.00601	-4.79
					(R)LIESPAPGIISR(R	99%	2.18	0.20		626.8637	1,251.71	2	-0.00601	-4.79
					(R)SVYEPLQTGLIA	100%	4.43	0.26	Oxidation (+16)	763.7399	2,288.20	3	-0.01103	-4.82
					(R)SVYEPLQTGLIA	100%	4.43	0.26	Oxidation (+16)	763.7399	2,288.20	3	-0.01103	-4.82
					(R)SVYEPLQTGLIA	100%	4.43	0.26	Oxidation (+16)	763.7399	2,288.20	3	-0.01103	-4.82
					(R)HTLIIYDDLSK(Q	100%	3.25	0.52		659.3539	1,316.69	2	-0.00466	-3.54
					(R)HTLIIYDDLSK(Q	100%	3.20	0.52		659.3545	1,316.69	2	-0.00344	-2.61
					(R)HTLIIYDDLSK(Q	100%	2.56	0.49		659.3534	1,316.69	2	-0.00564	-4.28
					(R)HTLIIYDDLSK(Q	100%	2.12	0.30		659.3543	1,316.69	2	-0.00380	-2.89
					(N)GYLDSLEIGQVR	100%	2.43	0.20		675.3539	1,348.69	2	-0.00554	-4.10
					(K)TFTEEAEALLK(I	100%	3.00	0.36		626.3244	1,250.63	2	-0.00532	-4.25
					(K)TFTEEAEALLK(I	100%	3.00	0.36		626.3244	1,250.63	2	-0.00532	-4.25
					(K)TFTEEAEALLK(I	100%	2.60	0.23		626.3242	1,250.63	2	-0.00569	-4.54
					(K)TFTEEAEALLK(I	100%	2.60	0.23		626.3242	1,250.63	2	-0.00569	-4.54
					(K)TFTEEAEALLK(I	100%	2.55	0.20		626.3254	1,250.64	2	-0.00337	-2.69
					(K)TFTEEAEALLK(I	100%	2.55	0.20		626.3254	1,250.64	2	-0.00337	-2.69
16	hypothetical protein P	gi|550327651	13	15%										
					(R)DLFHLLPDAFK(	100%	3.49	0.34		658.3545	1,314.69	2	-0.00304	-2.31
					(K)QIGVIGWGSQGI	100%	4.45	0.40		660.6838	1,979.03	3	-0.00429	-2.16
					(K)QIGVIGWGSQGI	100%	4.45	0.40		660.6838	1,979.03	3	-0.00429	-2.16
					(K)QIGVIGWGSQGI	100%	3.73	0.49		990.5239	1,979.03	2	-0.00079	-0.40
					(K)QIGVIGWGSQGI	100%	3.73	0.49		990.5239	1,979.03	2	-0.00079	-0.40
					(K)NISVIAVcPK(G)	100%	2.61	0.25	Carbamidomethyl (+57)	550.8080	1,099.60	2	-0.00450	-4.09
					(K)EGLPAFPMGK(I)	98%	2.17	0.22		523.7687	1,045.52	2	-0.00407	-3.89
					(K)EGLPAFPmGK(I)	96%	2.13	0.24	Oxidation (+16)	531.7660	1,061.52	2	-0.00436	-4.10
					(K)KGHSYSEIINESL	100%	3.11	0.18	Oxidation (+16)	618.7043	3,088.49	5	-0.00744	-2.41
					(K)KGHSYSEIINESL	99%	3.14	0.12	Oxidation (+16)	773.1289	3,088.49	4	-0.00597	-1.93
					(K)GHSYSEIINESLI	100%	3.73	0.18	Oxidation (+16)	741.1047	2,960.39	4	-0.00765	-2.58
					(R)GVSFmVDNcSTT	100%	3.38	0.49	Oxidation (+16), Carbamidomethyl (+57)	780.8407	1,559.67	2	-0.00401	-2.57
					(R)GVSFMVDNcSTT	100%	3.12	0.12	Carbamidomethyl (+57)	772.8454	1,543.68	2	0.00031	0.20
23	Citrate synthase famil	gi|550348021	4	12%										
					(K)VQLGNITVDmVI	100%	2.57	0.44	Oxidation (+16), Oxidation (+16)	867.9444	1,733.87	2	-0.00646	-3.72

					(K)VQLGNITVDmVI	100%	2.57	0.44	Oxidation (+16), Oxidation (+16)	867.9444	1,733.87	2	-0.00646	-3.72
					(R)GmTGLLWETSLI	100%	4.28	0.45	Oxidation (+16)	1060.0225	2,118.03	2	-0.00042	-0.20
					(K)NPWPNVDAHSG	100%	3.92	0.26		862.7628	2,585.27	3	0.00025	0.10
27	3-phosphoglycerate ki	gi|500229492	6	17%										
					(K)FLKPSVAGFLLQ	98%	2.36	0.17		724.4347	1,446.85	2	-0.00524	-3.62
					(K)RPFAAIVGGSK(V	100%	3.75	0.11		368.2156	1,101.63	3	-0.00449	-4.07
					(K)IGVIESLLEK(C)	100%	3.38	0.22		550.8298	1,099.65	2	-0.00396	-3.59
					(K)IGVIESLLEK(C)	100%	3.38	0.22		550.8298	1,099.65	2	-0.00396	-3.59
					(K)GVTTIIGGGDSV.	100%	3.41	0.32		787.4233	1,572.83	2	-0.00417	-2.65
					(K)GVTTIIGGGDSV.	100%	3.67	0.18		787.4232	1,572.83	2	-0.00441	-2.80
3	hypothetical protein A	gi|548847307	18	10%										
					(K)NGNTGYDEIR(A	100%	2.83	0.40		569.7571	1,137.50	2	-0.00544	-4.78
					(K)VTTTIGYGSPNK	100%	2.75	0.39		619.3217	1,236.63	2	-0.00641	-5.18
					(K)ANSYSVHGSAL(	100%	2.97	0.36		681.3415	1,360.67	2	-0.00504	-3.70
					(R)NLSQQcLNALAK	100%	3.07	0.33	Carbamidomethyl (+57)	680.3541	1,358.69	2	-0.00416	-3.06
					(R)NLSQQcLNALAK	100%	3.07	0.33	Carbamidomethyl (+57)	680.3541	1,358.69	2	-0.00416	-3.06
					(R)NLSQQcLNALAK	100%	3.07	0.33	Carbamidomethyl (+57)	680.3541	1,358.69	2	-0.00416	-3.06
					(K)VLPGLLGGSADI	100%	6.26	0.60	Oxidation (+16)	1037.0663	2,072.12	2	-0.00107	-0.52
					(K)VLPGLLGGSADI	100%	6.26	0.60	Oxidation (+16)	1037.0663	2,072.12	2	-0.00107	-0.52
					(K)VLPGLLGGSADI	100%	6.26	0.60	Oxidation (+16)	1037.0663	2,072.12	2	-0.00107	-0.52
					(K)VLPGLLGGSADI	100%	6.26	0.60	Oxidation (+16)	1037.0663	2,072.12	2	-0.00107	-0.52
					(K)VLPGLLGGSADI	100%	3.68	0.45	Oxidation (+16)	691.7108	2,072.11	3	-0.00865	-4.17
					(K)VLPGLLGGSADI	100%	3.68	0.45	Oxidation (+16)	691.7108	2,072.11	3	-0.00865	-4.17
					(K)VLPGLLGGSADI	100%	3.68	0.45	Oxidation (+16)	691.7108	2,072.11	3	-0.00865	-4.17
					(K)VLPGLLGGSADI	100%	3.68	0.45	Oxidation (+16)	691.7108	2,072.11	3	-0.00865	-4.17
					(K)VLPGLLGGSADI	100%	2.29	0.29		1029.0679	2,056.12	2	-0.00298	-1.45
					(K)VLPGLLGGSADI	100%	2.29	0.29		1029.0679	2,056.12	2	-0.00298	-1.45
					(K)VLPGLLGGSADI	100%	2.29	0.29		1029.0679	2,056.12	2	-0.00298	-1.45
					(K)VLPGLLGGSADI	100%	2.29	0.29		1029.0679	2,056.12	2	-0.00298	-1.45
31	3-phosphoglycerate ki	gi|500229492	12	29%										
					(R)FYKEEEKNDPEF	100%	3.60	0.19		887.4192	1,772.82	2	-0.00204	-1.15
					(R)FYKEEEKNDPEF	99%	3.31	0.12		591.9463	1,772.82	3	-0.00883	-4.98
					(K)FLKPSVAGFLLQ	100%	3.89	0.11		483.2929	1,446.86	3	-0.00308	-2.13
					(K)FLKPSVAGFLLQ	99%	2.50	0.21		724.4348	1,446.86	2	-0.00512	-3.53
					(K)RPFAAIVGGSK(V	100%	3.74	0.12		551.8198	1,101.63	2	-0.00447	-4.06
					(K)RPFAAIVGGSK(V	100%	2.59	0.19		368.2157	1,101.63	3	-0.00430	-3.90
					(K)cDILLLGGGmIFT	100%	5.73	0.53	Carbamidomethyl (+57), Oxidation (+16)	932.4728	1,862.93	2	-0.00045	-0.24
					(K)AQGLSVGSSLVE	100%	3.02	0.45		759.8851	1,517.76	2	-0.00188	-1.24
					(K)AQGLSVGSSLVE	100%	3.02	0.45		759.8851	1,517.76	2	-0.00188	-1.24
					(K)GVTTIIGGGDSV.	100%	4.88	0.21		787.4224	1,572.83	2	-0.00600	-3.81
					(K)GVTTIIGGGDSV.	100%	4.25	0.22		787.4229	1,572.83	2	-0.00502	-3.19
					(K)GVTTIIGGGDSV.	100%	3.26	0.18		787.4229	1,572.83	2	-0.00502	-3.19
35	RecName: Full=Photo	gi|75252730	8	20%										
					(R)SIPSAEDEDFNYI	100%	2.62	0.39		771.8370	1,541.66	2	-0.00407	-2.64
					(Q)SAEmVTDEGAIY	100%	3.60	0.30	Oxidation (+16)	929.9269	1,857.84	2	-0.00228	-1.23
					(Q)SAEMVTDEGAIY	100%	3.33	0.25		921.9329	1,841.85	2	0.00460	2.50
					(K)AAVQETVSATLI	100%	3.63	0.16		680.3615	1,358.71	2	-0.00690	-5.08
					(R)TVSSGISGASYY	100%	5.02	0.56		1091.5233	2,181.03	2	-0.00169	-0.78
					(R)TVSSGISGASYY	100%	3.47	0.20		728.0157	2,181.03	3	-0.00836	-3.83
					(R)ADGGLWLLVR(	100%	3.53	0.25		550.3145	1,098.61	2	-0.00429	-3.91
					(R)GFGILDVGYR(S)	100%	3.34	0.34		548.7913	1,095.57	2	-0.00350	-3.19
43	4-nitrophenylphosphat	gi|413918758	6	16%										
					(R)LVFVTNNSTK(S)	99%	2.25	0.24		561.8091	1,121.60	2	-0.00446	-3.97
					(K)KVYVIGEEGILK	99%	3.18	0.10		449.9331	1,346.78	3	-0.00359	-2.66
					(K)VYVIGEEGILK(E	100%	3.22	0.35		610.3468	1,218.68	2	-0.00712	-5.84
					(R)ENPGcLFIATNR(	100%	3.24	0.30	Carbamidomethyl (+57)	696.3388	1,390.66	2	-0.00338	-2.43
					(T)SQIcMVGDR(L)	100%	2.91	0.18	Carbamidomethyl (+57)	533.2408	1,064.47	2	-0.00742	-6.96
					(R)LDTDILFGQNGG	100%	3.35	0.36	Carbamidomethyl (+57)	769.3657	1,536.72	2	-0.00774	-5.04
48	chloroplast pigment-bi	gi|558695556	8	14%										
					(R)WAmLGAAGFIIP	100%	4.90	0.49	Oxidation (+16)	926.4759	1,850.94	2	-0.00186	-1.00
					(R)WAMLGAAGFIIP	100%	5.36	0.45		918.4788	1,834.94	2	-0.00120	-0.66
					(K)YGANcGPEAVW	100%	4.28	0.47	Carbamidomethyl (+57)	749.8409	1,497.67	2	-0.00393	-2.62
					(N)cGPEAVWFK(T)	100%	2.20	0.32	Carbamidomethyl (+57)	547.2599	1,092.51	2	-0.00111	-1.02
					(K)DPDQAALLK(V)	100%	2.85	0.31		485.7617	969.51	2	-0.00442	-4.56
					(K)DPDQAALLK(V)	100%	2.85	0.31		485.7617	969.51	2	-0.00442	-4.56
					(K)DPDQAALLK(V)	100%	2.62	0.33		485.7621	969.51	2	-0.00369	-3.80
					(K)DPDQAALLK(V)	100%	2.62	0.33		485.7621	969.51	2	-0.00369	-3.80
52	chloroplast pigment-bi	gi|558695556	7	14%										
					(R)WAmLGAAGFIIP	100%	4.87	0.49	Oxidation (+16)	926.4741	1,850.93	2	-0.00540	-2.91
					(K)YGANcGPEAVW	100%	3.58	0.39	Carbamidomethyl (+57)	749.8378	1,497.66	2	-0.01015	-6.77
					(N)cGPEAVWFK(T)	100%	2.78	0.35	Carbamidomethyl (+57)	547.2565	1,092.50	2	-0.00795	-7.27
					(K)DPDQAALLK(V)	100%	3.19	0.32		485.7603	969.51	2	-0.00723	-7.45
					(K)DPDQAALLK(V)	100%	3.19	0.32		485.7603	969.51	2	-0.00723	-7.45
					(K)DPDQAALLK(V)	100%	2.65	0.27		485.7623	969.51	2	-0.00339	-3.49
					(K)DPDQAALLK(V)	100%	2.65	0.27		485.7623	969.51	2	-0.00339	-3.49
54	ascorbate peroxidase [	gi|6651272	6	17%										
					(R)LPNATLGNDHLI	100%	3.46	0.27		660.8518	1,319.69	2	-0.00571	-4.32
					(R)LPNATLGNDHLI	100%	3.47	0.24		440.9041	1,319.69	3	-0.00444	-3.36
					(K)DIVTLSGGHTLG	100%	3.18	0.30		663.3607	1,324.71	2	-0.00341	-2.57
					(K)DIVTLSGGHTLG	100%	3.18	0.30		663.3607	1,324.71	2	-0.00341	-2.57
					(K)YAcDEDAFFADY	100%	3.11	0.46	Carbamidomethyl (+57)	1085.4286	2,168.84	2	-0.00301	-1.39
					(K)YAcDEDAFFADY	100%	2.27	0.24	Carbamidomethyl (+57), Oxidation (+16)	1093.4266	2,184.84	2	-0.00183	-0.84

59	unnamed protein prod	gi|297741493	4	17%										
					(R)LAWHSAGTYDV	100%	2.93	0.34		674.3366	1,346.66	2	-0.00331	-2.45
					(R)LAWHSAGTYDV	100%	2.93	0.34		674.3366	1,346.66	2	-0.00331	-2.45
					(G)HmGLSDKDIVAI	100%	3.17	0.17	Oxidation (+16)	694.0213	2,079.04	3	-0.01141	-5.49
					(K)EGLIQLPSDK(A)	99%	2.51	0.16		550.3009	1,098.59	2	-0.00512	-4.65
64	TPA: hypothetical pro	gi|414878632	4	11%										
					(R)LYSIASSALGDF	100%	3.97	0.49		815.9008	1,629.79	2	-0.00169	-1.03
					(R)LYSIASSALGDF	100%	3.97	0.49		815.9008	1,629.79	2	-0.00169	-1.03
					(R)LVYTNDQGEVV	100%	2.46	0.27		682.8525	1,363.69	2	-0.00794	-5.82
					(K)DNTYVYmcGLK	100%	2.14	0.22	Oxidation (+16), Carbamidomethyl (+57)	690.3005	1,378.59	2	-0.00345	-2.50
66	hypothetical protein E	gi|557113323	6	9%										
					(Q)DLYDSIAAGNYI	98%	2.39	0.21		871.4099	1,740.81	2	0.00548	3.15
					(K)TWPEDILPLQPV	100%	3.20	0.28		810.9430	1,619.87	2	0.00396	2.45
					(K)TWPEDILPLQPV	100%	3.20	0.28		810.9430	1,619.87	2	0.00396	2.45
					(K)TWPEDILPLQPV	100%	3.20	0.28		810.9430	1,619.87	2	0.00396	2.45
					(R)LGPNYLQLPVN	100%	3.17	0.24		762.4320	1,522.85	2	-0.00151	-0.99
					(R)LGPNYLQLPVN	100%	3.17	0.24		762.4320	1,522.85	2	-0.00151	-0.99
69	TPA: hypothetical pro	gi|414878632	7	13%										
					(R)LYSIASSALGDF	100%	2.46	0.28		815.8979	1,629.78	2	-0.00755	-4.63
					(R)LYSIASSALGDF	100%	2.46	0.28		815.8979	1,629.78	2	-0.00755	-4.63
					(R)LYSIASSALGDF	100%	2.43	0.26		815.9016	1,629.79	2	-0.00010	-0.06
					(R)LYSIASSALGDF	100%	2.43	0.26		815.9016	1,629.79	2	-0.00010	-0.06
					(R)LVYTNDQGEVV	100%	3.49	0.25		682.8532	1,363.69	2	-0.00660	-4.84
					(R)LDFAVSR(E)	95%	2.23	0.13		404.2205	806.43	2	-0.00224	-2.78
					(K)DNTYVYmcGLK	100%	2.28	0.37	Oxidation (+16), Carbamidomethyl (+57)	690.3008	1,378.59	2	-0.00272	-1.97
70	hypothetical protein E	gi|557090446	19	12%										
					(R)LYSIASSAIGDFC	100%	4.86	0.56		815.8990	1,629.78	2	-0.00535	-3.28
					(R)LYSIASSAIGDFC	100%	4.86	0.56		815.8990	1,629.78	2	-0.00535	-3.28
					(R)LYSIASSAIGDFC	100%	3.77	0.56		815.8971	1,629.78	2	-0.00913	-5.60
					(R)LYSIASSAIGDFC	100%	3.77	0.56		815.8971	1,629.78	2	-0.00913	-5.60
					(R)LYSIASSAIGDFC	100%	4.13	0.47		815.9008	1,629.79	2	-0.00181	-1.11
					(R)LYSIASSAIGDFC	100%	4.13	0.47		815.9008	1,629.79	2	-0.00181	-1.11
					(R)LYSIASSAIGDFC	100%	3.97	0.47		815.8992	1,629.78	2	-0.00498	-3.06
					(R)LYSIASSAIGDFC	100%	3.97	0.47		815.8992	1,629.78	2	-0.00498	-3.06
					(R)LYSIASSAIGDFC	100%	3.47	0.48		815.8973	1,629.78	2	-0.00877	-5.38
					(R)LYSIASSAIGDFC	100%	3.47	0.48		815.8973	1,629.78	2	-0.00877	-5.38
					(R)LYSIASSAIGDFC	100%	2.15	0.36		815.8979	1,629.78	2	-0.00742	-4.55
					(R)LYSIASSAIGDFC	100%	2.15	0.36		815.8979	1,629.78	2	-0.00742	-4.55
					(R)LDFAVSR(E)	96%	2.24	0.11		404.2203	806.43	2	-0.00255	-3.16
					(K)KDNTFVYMcGLI	98%	2.21	0.18	Carbamidomethyl (+57)	738.3526	1,474.69	2	-0.00439	-2.97
					(K)KDNTFVYmcGLI	96%	2.39	0.13	Oxidation (+16), Carbamidomethyl (+57)	746.3494	1,490.68	2	-0.00577	-3.87
					(K)DNTFVYMcGLK	100%	2.93	0.40	Carbamidomethyl (+57)	674.3029	1,346.59	2	-0.00888	-6.59
					(K)DNTFVYmcGLK(	100%	3.08	0.31	Oxidation (+16), Carbamidomethyl (+57)	682.3014	1,362.59	2	-0.00672	-4.93
					(K)DGIDWLEYK(K)	100%	2.51	0.25		569.7696	1,137.52	2	-0.00984	-8.64
					(K)DGIDWLEYK(K)	100%	2.51	0.25		569.7696	1,137.52	2	-0.00984	-8.64
75	RecName: Full=Glyce	gi|120665	20	15%										
					(R)KDSPLDVVVVN	100%	3.95	0.29		576.6409	1,726.90	3	-0.00941	-5.45
					(R)KDSPLDVVVVN	100%	4.96	0.19		864.4598	1,726.91	2	-0.00537	-3.11
					(R)KDSPLDVVVVN	100%	3.34	0.14		576.6409	1,726.90	3	-0.00941	-5.45
					(K)DSPLDVVVVND	100%	5.55	0.20		800.4120	1,598.81	2	-0.00595	-3.72
					(K)DSPLDVVVVND	100%	3.96	0.25		800.4124	1,598.81	2	-0.00509	-3.18
					(K)DSPLDVVVVND	100%	4.11	0.23		800.4119	1,598.81	2	-0.00607	-3.79
					(K)DSPLDVVVVND	100%	3.06	0.31		800.4122	1,598.81	2	-0.00546	-3.41
					(K)DSPLDVVVVND	100%	3.31	0.25		800.4116	1,598.81	2	-0.00668	-4.18
					(K)DSPLDVVVVND	100%	3.35	0.20		800.4122	1,598.81	2	-0.00558	-3.49
					(K)DSPLDVVVVND	100%	2.87	0.18		800.4125	1,598.81	2	-0.00485	-3.03
					(K)YDSmLGTFK(A)	100%	2.53	0.44	Oxidation (+16)	539.2469	1,076.48	2	-0.00576	-5.35
					(K)YDSMLGTFK(A)	100%	2.53	0.38		531.2494	1,060.48	2	-0.00584	-5.51
					(K)YDSmLGTFK(A)	100%	2.13	0.45	Oxidation (+16)	539.2476	1,076.48	2	-0.00442	-4.10
					(K)KVIITAPAK(G)	100%	2.41	0.19		470.8115	939.61	2	-0.00344	-3.65
					(K)KVIITAPAK(G)	100%	2.41	0.19		470.8115	939.61	2	-0.00344	-3.65
					(R)VPTPNVSVVDL	100%	5.61	0.54		875.5062	1,749.00	2	-0.00577	-3.30
					(R)VPTPNVSVVDL	100%	4.66	0.34		584.0050	1,748.99	3	-0.01048	-5.99
					(R)VPTPNVSVVDL	100%	3.04	0.34		875.5067	1,749.00	2	-0.00492	-2.81
					(K)GITAEDVNAAF	99%	2.41	0.20		632.3181	1,262.62	2	-0.00389	-3.08
					(K)GITAEDVNAAF	99%	2.41	0.20		632.3181	1,262.62	2	-0.00389	-3.08
78	hypothetical protein C	gi|557540310	10	12%										
					(K)IGINGFGR(I)	99%	2.27	0.16		417.2336	832.45	2	-0.00300	-3.60
					(K)IGINGFGR(I)	99%	2.27	0.16		417.2336	832.45	2	-0.00300	-3.60
					(K)YDSVHGQWK(H	100%	2.72	0.41		560.2598	1,118.51	2	-0.00959	-8.57
					(K)KVVISAPSK(D)	99%	2.23	0.20		464.7926	927.57	2	-0.00472	-5.08
					(K)LVSWYDNEWG	100%	3.86	0.52		881.3944	1,760.77	2	-0.00510	-2.90
					(K)LVSWYDNEWG	100%	3.86	0.52		881.3944	1,760.77	2	-0.00510	-2.90
					(K)LVSWYDNEWG	100%	3.13	0.44		881.3928	1,760.77	2	-0.00828	-4.70
					(K)LVSWYDNEWG	100%	3.13	0.44		881.3928	1,760.77	2	-0.00828	-4.70
					(K)LVSWYDNEWG	100%	2.40	0.30		881.3956	1,760.78	2	-0.00266	-1.51
					(K)LVSWYDNEWG	100%	2.40	0.30		881.3956	1,760.78	2	-0.00266	-1.51
80	hypothetical protein C	gi|557524521	15	21%										
					(R)RIVEADEFMR(A	100%	2.28	0.29		633.3174	1,264.62	2	-0.00326	-2.57
					(R)IVEADEFMR(A)	100%	2.59	0.26		555.2667	1,108.52	2	-0.00348	-3.13
					(R)IVEADEFmR(A)	100%	2.43	0.27	Oxidation (+16)	563.2636	1,124.51	2	-0.00462	-4.10
					(R)IVEADEFmR(A)	100%	2.27	0.20	Oxidation (+16)	563.2631	1,124.51	2	-0.00559	-4.97
					(K)GQTVGVIGAGR(	100%	3.09	0.38		507.7857	1,013.56	2	-0.00505	-4.98
					(K)GQTVGVIGAGR(	100%	2.53	0.32		507.7861	1,013.56	2	-0.00438	-4.32

					(K)MNLIYYDLYQA	100%	4.40	0.34		832.4086	1,662.80	2	-0.00496	-2.98
					(K)ANGEQPVTWK(	100%	2.64	0.14		565.2829	1,128.55	2	-0.00523	-4.63
					(K)KEAILVNcSR(G)	100%	2.22	0.29	Carbamidomethyl (+57)	595.3184	1,188.62	2	-0.00625	-5.25
					(K)EAILVNcSR(G)	100%	2.28	0.22	Carbamidomethyl (+57)	531.2716	1,060.53	2	-0.00488	-4.59
					(K)NAIVVPHIASAS	100%	2.66	0.22		653.8754	1,305.74	2	-0.00429	-3.28
					(R)EGmATLAALNV	100%	3.49	0.38	Oxidation (+16)	702.3790	1,402.74	2	-0.00568	-4.05
					(R)EGmATLAALNV	100%	3.72	0.31	Oxidation (+16)	702.3796	1,402.74	2	-0.00446	-3.18
					(R)EGMATLAALNV	100%	2.91	0.38		694.3824	1,386.75	2	-0.00405	-2.92
					(R)EGmATLAALNV	100%	2.81	0.36	Oxidation (+16)	702.3785	1,402.74	2	-0.00666	-4.74
82	hypothetical protein C	gi|557548860	6	11%										
					(R)MDEWGIDVALT	100%	3.20	0.37		825.3907	1,648.77	2	-0.01015	-6.15
					(K)ALSLPTGMGIVc	100%	2.57	0.32	Carbamidomethyl (+57)	801.4200	1,600.83	2	-0.00640	-4.00
					(Q)RYNLSLGLGLN	98%	3.02	0.11		449.9285	1,346.76	3	-0.00360	-2.67
					(R)YNLSLGLGLNK(	100%	3.13	0.23		596.3389	1,190.66	2	-0.00289	-2.42
					(R)YNLSLGLGLNK(	100%	3.13	0.23		596.3389	1,190.66	2	-0.00289	-2.42
					(R)YNLSLGLGLNK(	100%	3.13	0.23		596.3389	1,190.66	2	-0.00289	-2.42
83	RecName: Full=3-ket	gi|73919871	6	17%										
					(K)DTYPDDLLAPVI	100%	3.36	0.39		744.3883	1,486.76	2	-0.00501	-3.37
					(K)TNLNPSEVGDIV	100%	4.39	0.45		775.0761	2,322.21	3	-0.01167	-5.02
					(R)QcSSGLQAVAD	100%	4.48	0.46	Carbamidomethyl (+57)	844.9334	1,687.85	2	-0.00417	-2.47
					(R)TFAAVGVDPAI	100%	5.39	0.32	Oxidation (+16)	784.7681	2,351.28	3	-0.01022	-4.34
					(R)TFAAVGVDPAI	100%	5.25	0.34	Oxidation (+16)	1176.6528	2,351.29	2	-0.00147	-0.63
					(R)TFAAVGVDPAI	100%	4.13	0.36		1168.6522	2,335.29	2	-0.00778	-3.33
84	hypothetical protein C	gi|557524521	15	19%										
					(R)RIVEADEFMR(A	100%	2.78	0.35		633.3182	1,264.62	2	-0.00167	-1.32
					(R)IVEADEFmR(A)	100%	3.13	0.25	Oxidation (+16)	563.2633	1,124.51	2	-0.00523	-4.64
					(R)IVEADEFMR(A)	100%	2.74	0.30		555.2675	1,108.52	2	-0.00201	-1.81
					(R)IVEADEFmR(A)	100%	2.14	0.24	Oxidation (+16)	563.2642	1,124.51	2	-0.00340	-3.02
					(K)GQTVGVIGAGR(	100%	2.96	0.45		507.7859	1,013.56	2	-0.00481	-4.74
					(K)GQTVGVIGAGR(	100%	3.23	0.36		507.7860	1,013.56	2	-0.00456	-4.50
					(K)GQTVGVIGAGR(	96%	2.08	0.25		507.7862	1,013.56	2	-0.00420	-4.14
					(K)MNLIYYDLYQA	100%	4.30	0.35		832.4101	1,662.81	2	-0.00203	-1.22
					(K)mNLIYYDLYQA	100%	4.23	0.29	Oxidation (+16)	840.4072	1,678.80	2	-0.00281	-1.67
					(K)EAILVNcSR(G)	100%	2.65	0.19	Carbamidomethyl (+57)	531.2716	1,060.53	2	-0.00488	-4.59
					(K)NAIVVPHIASAS	100%	2.76	0.19		653.8754	1,305.74	2	-0.00417	-3.19
					(R)EGmATLAALNV	100%	3.83	0.37	Oxidation (+16)	702.3798	1,402.75	2	-0.00422	-3.00
					(R)EGmATLAALNV	100%	3.61	0.37	Oxidation (+16)	702.3802	1,402.75	2	-0.00336	-2.39
					(R)EGmATLAALNV	100%	2.47	0.33	Oxidation (+16)	702.3765	1,402.74	2	-0.01069	-7.61
					(R)EGmATLAALNV	100%	2.31	0.28	Oxidation (+16)	702.3793	1,402.74	2	-0.00507	-3.61
85	PREDICTED: outer p	gi|502090241	15	9%										
					(K)KGELFLADVNT	100%	3.59	0.32		788.4385	1,574.86	2	-0.00467	-2.96
					(K)KGELFLADVNT	100%	3.59	0.32		788.4385	1,574.86	2	-0.00467	-2.96
					(K)KGELFLADVNT	100%	3.70	0.24		525.9606	1,574.86	3	-0.00719	-4.56
					(K)KGELFLADVNT	100%	3.70	0.24		525.9606	1,574.86	3	-0.00719	-4.56
					(K)KGELFLADVNT	100%	3.95	0.18		525.9610	1,574.86	3	-0.00590	-3.75
					(K)KGELFLADVNT	100%	3.95	0.18		525.9610	1,574.86	3	-0.00590	-3.75
					(K)KGELFLADVNT	100%	3.11	0.26		788.4363	1,574.86	2	-0.00894	-5.67
					(K)KGELFLADVNT	100%	3.11	0.26		788.4363	1,574.86	2	-0.00894	-5.67
					(K)KGELFLADVNT	95%	2.85	0.13		788.4365	1,574.86	2	-0.00870	-5.52
					(K)KGELFLADVNT	95%	2.85	0.13		788.4365	1,574.86	2	-0.00870	-5.52
					(K)GELFLADVNTQ	100%	3.07	0.36		724.3915	1,446.77	2	-0.00354	-2.45
					(K)GELFLADVNTQ	100%	3.07	0.36		724.3915	1,446.77	2	-0.00354	-2.45
					(K)SLFTISGEVDTK(	100%	3.43	0.13		648.8352	1,295.66	2	-0.00521	-4.02
					(K)SLFTISGEVDTK(	100%	3.39	0.12		648.8347	1,295.65	2	-0.00631	-4.87
					(K)SLFTISGEVDTK(	100%	3.05	0.12		648.8358	1,295.66	2	-0.00399	-3.08
88	triosephosphate isome	gi|223531284	2	8%										
					(K)TFDVcFQQLK(A	100%	2.54	0.32	Carbamidomethyl (+57)	643.3142	1,284.61	2	-0.00357	-2.78
					(K)GPEFATIINSVTS	100%	4.19	0.53		732.3867	1,462.76	2	-0.00811	-5.54
91	hypothetical protein E	gi|557090446	7	10%										
					(R)LYSIASSAIGDF	100%	3.27	0.50		815.8986	1,629.78	2	-0.00608	-3.73
					(R)LYSIASSAIGDF	100%	3.27	0.50		815.8986	1,629.78	2	-0.00608	-3.73
					(R)LYSIASSAIGDF	100%	2.35	0.36		815.8975	1,629.78	2	-0.00840	-5.15
					(R)LYSIASSAIGDF	100%	2.35	0.36		815.8975	1,629.78	2	-0.00840	-5.15
					(K)DNTFVYmcGLK(	100%	2.58	0.26	Oxidation (+16), Carbamidomethyl (+57)	682.3007	1,362.59	2	-0.00806	-5.91
					(K)DGIDWLEYK(K)	100%	3.03	0.36		569.7711	1,137.53	2	-0.00691	-6.07
					(K)DGIDWLEYK(K)	100%	3.03	0.36		569.7711	1,137.53	2	-0.00691	-6.07
93	hypothetical protein C	gi|557553557	11	11%										
					(K)LNLGVGAYR(T)	100%	2.82	0.21		481.7729	961.53	2	-0.00341	-3.54
					(K)LNLGVGAYR(T)	100%	2.82	0.21		481.7729	961.53	2	-0.00341	-3.54
					(K)LNLGVGAYR(T)	97%	2.27	0.17		481.7725	961.53	2	-0.00408	-4.24
					(K)LNLGVGAYR(T)	97%	2.27	0.17		481.7725	961.53	2	-0.00408	-4.24
					(K)EYLPIEGLAAFN	100%	3.04	0.34		732.8871	1,463.76	2	-0.00655	-4.47
					(Q)RVATVQGLSGT	100%	3.00	0.32		751.4224	1,500.83	2	-0.00707	-4.71
					(R)VATVQGLSGTG	100%	3.48	0.41		673.3708	1,344.73	2	-0.00912	-6.78
					(R)VATVQGLSGTG	100%	2.92	0.27		673.3724	1,344.73	2	-0.00607	-4.51
					(R)LAAALIER(Y)	99%	2.61	0.10		428.7645	855.51	2	-0.00337	-3.94
					(R)LAAALIER(Y)	99%	2.61	0.10		428.7645	855.51	2	-0.00337	-3.94
					(K)WHVYMTK(D)	100%	2.01	0.28		482.7380	963.46	2	-0.00221	-2.29
94	hypothetical protein C	gi|557534509	8	12%										
					(K)VANPIVEmDGD	100%	3.02	0.41	Oxidation (+16), Oxidation (+16)	854.8769	1,707.74	2	-0.00537	-3.14
					(R)LIDDMVAYALK	100%	2.30	0.31		626.3328	1,250.65	2	-0.00730	-5.83
					(R)LIDDMVAYALK	100%	2.30	0.31		626.3328	1,250.65	2	-0.00730	-5.83
					(K)TIEAEAAHGTVT	100%	2.84	0.40		678.3448	1,354.68	2	-0.00915	-6.75

					(K)TIEAEAAHGTVT	100%	2.84	0.40		678.3448	1,354.68	2	-0.00915	-6.75
					(K)DLALIIHGSK(M)	97%	2.21	0.18		533.8135	1,065.61	2	-0.00607	-5.69
					(K)DLALIIHGSK(M)	97%	2.21	0.18		533.8135	1,065.61	2	-0.00607	-5.69
					(K)DLALIIHGSK(M)	97%	2.21	0.18		533.8135	1,065.61	2	-0.00607	-5.69
97	RecName: Full=Sedol	gi|1173347	17	24%										
					(R)TAScGGTAcVNS	100%	4.57	0.46	Carbamidomethyl (+57), Carbamidomethyl (+57)	906.3978	2,716.17	3	-0.00673	-2.48
					(R)TAScGGTAcVNS	100%	4.60	0.36	Carbamidomethyl (+57), Carbamidomethyl (+57), Oxi	911.7311	2,732.17	3	-0.00153	-0.56
					(R)TAScGGTAcVNS	100%	2.77	0.50	Carbamidomethyl (+57), Carbamidomethyl (+57), Oxi	1367.0898	2,732.17	2	-0.00798	-2.92
					(R)TAScGGTAcVNS	100%	2.98	0.44	Carbamidomethyl (+57), Carbamidomethyl (+57)	1359.0878	2,716.16	2	-0.01722	-6.34
					(G)DQVAAAmGIYG	100%	3.94	0.41	Oxidation (+16)	682.8324	1,363.65	2	-0.00534	-3.92
					(G)DQVAAAmGIYG	100%	2.89	0.44	Oxidation (+16)	682.8304	1,363.65	2	-0.00937	-6.87
					(K)mFSPGNLR(A)	97%	2.17	0.21	Oxidation (+16)	469.2300	936.45	2	-0.00332	-3.54
					(R)ATFDNPDYDK(L	100%	2.68	0.38		593.2537	1,184.49	2	-0.00588	-4.96
					(R)YTGGmVPDVNQ	100%	4.03	0.43	Oxidation (+16)	825.4319	1,648.85	2	-0.00035	-0.21
					(R)YTGGmVPDVNQ	100%	4.03	0.43	Oxidation (+16)	825.4319	1,648.85	2	-0.00035	-0.21
					(R)YTGGmVPDVNQ	100%	3.23	0.31	Oxidation (+16)	825.4310	1,648.85	2	-0.00218	-1.32
					(R)YTGGmVPDVNQ	100%	3.23	0.31	Oxidation (+16)	825.4310	1,648.85	2	-0.00218	-1.32
					(R)YTGGMVPDVNQ	100%	3.11	0.31		817.4323	1,632.85	2	-0.00482	-2.95
					(R)YTGGMVPDVNQ	100%	3.11	0.31		817.4323	1,632.85	2	-0.00482	-2.95
					(R)LLFEVAPLGFLIE	100%	3.83	0.34		794.9688	1,587.92	2	-0.00492	-3.10
					(R)FEETLYGSSR(L)	100%	2.75	0.27		594.7776	1,187.54	2	-0.00525	-4.42
					(R)FEETLYGSSR(L)	99%	2.80	0.14		594.7771	1,187.54	2	-0.00622	-5.24

Table S4: Identification of significantly altered proteins after 56 kg N/ha treatment by LC-MS/MS

Pos. No.[a]	Protein Name	*Organism*	Sequence Similarity (%) [b]	Expression Profile [c]	Two-way ANOVA [d]			Cellular location [e]	Functional Class
				A0 B0 W0 A56 B56 W56	Genotype	Nitrogen	Interaction		
2	Transketolase family protein	*P. trichocarpa*		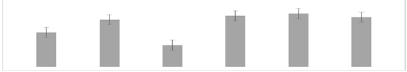	NS	*	NS	CHL	OPP
3	Transketolase	*A. trichopoda*	100	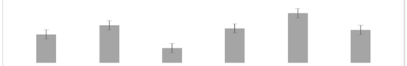	*	*	NS	CHL	OPP
4	Transketolase family protein	*P. trichocarpa*		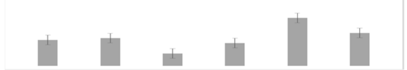	NS	*	NS	CHL	OPP
6	Phosphoglucomutase	*V. vinifera*		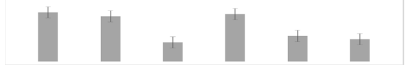	*	NS	NS	CYT	GS
8	Phosphoglucomutase	*P. tremula*		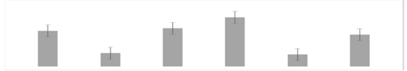	*	NS	NS	CYT	GS
9	Phosphoglucomutase	*P. tremula*		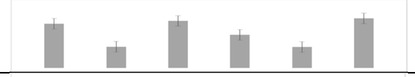	*	NS	NS	CYT	GS
10	Chaperonin 60 subunit β 1	*C. sativa*	97	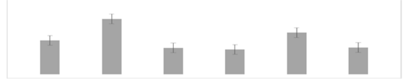	*	NS	NS	CHL	PM
11	ATP-dependent zinc metalloprotease FTSH 1	*S. italica*	97	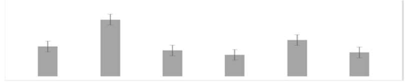	*	NS	NS	CHL	PM
13	ATP synthase CF1 α subunit	*C. maculata*		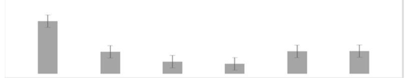	NS	NS	*	CHL	PS
14	ATP synthase CF1 α subunit	*I. cornuta*		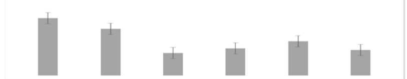	*	*	*	CHL	PS
15	ATP synthase CF1 α subunit, partial	*V. acerifolium*		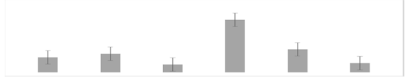	*	*	NS	CHL	PS
18	Catalase, partial	*B. juncea*		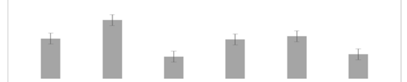	*	NS	NS	PER	RX
19	Catalase	*A. alpina*	99	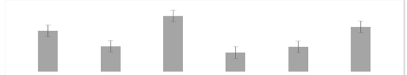	*	NS	NS	PER	RX

20	RubisCO activase α form precursor	*D. antarctica*		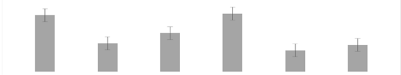	*	NS	NS	CHL	PS
25	GDP-mannose 3,5-epimerase isoform X2	*C. sativa*	98	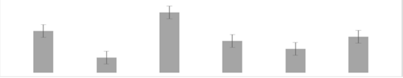	*	NS	*	GA	RX
27	Phosphoglycerate kinase	*Z. mays*		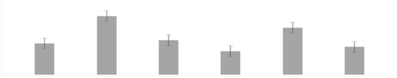	*	NS	NS	CHL	PS
29	Monodehydroascorbate reductase family protein	*P. trichocarpa*		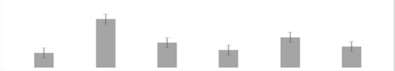	*	NS	NS	CYT	RX
30	Phosphoglycerate kinase	*N. tabacum*		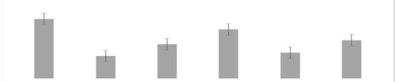	*	NS	NS	CHL	PS
32	Elongation factor, GTP-binding domain-containing protein	*C. cardunculus*		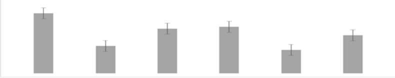	*	NS	NS	MC	PM
33	Glutamine synthetase nodule isozyme	*T. hassleriana*		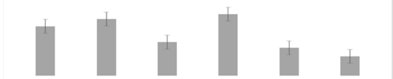	*	*	*	CYT	NM
35	Chloroplast RubisCO activase large protein isoform	*A. rubrum*	91	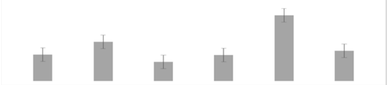	*	*	*	CHL	PS
36	Putative lactoylglutathione lyase	*C. sinensis*	98	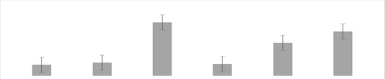	*	NS	NS	PER	BX
37	Photosystem II stability/assembly factor HCF136	*O. sativa subsp. japonica*		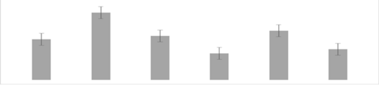	*	*	NS	CHL	PM
38	Probable fructose-bisphosphate aldolase 1	*B. oleracea var. oleracea*	96	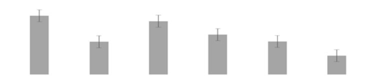	NS	*	NS	CHL	PS
39	Fructose-bisphosphate aldolase 1	*T. cacao*	80	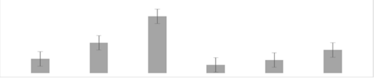	*	*	NS	CHL	PS
40	Photosystem II stability/assembly factor HCF136	*O. sativa subsp. japonica*		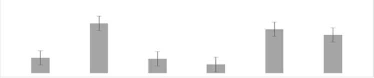	*	NS	*	CHL	PM
41	Oxygen-evolving enhancer protein 1	*S. lycopersicum*		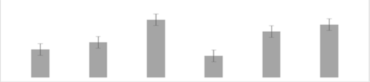	*	NS	NS	CHL	PS

42	Monomeric lectin SNAlm precursor	*S. nigra*		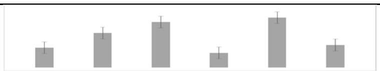	*	NS	*	Unkown	NA
43	4-Nitrophenylphosphatase	*Z. mays*		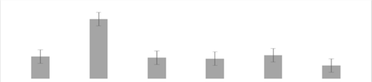	*	*	*	CHL	MCM
46	Chlorophyll a-b binding protein of LHCII type 1-like	*V. radiata*		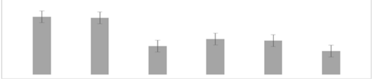	*	*	NS	CHL	PS
47	Ras-related protein Rab7 isoform X2	*C. sinensis*	99	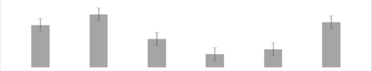	NS	*	*	MC	SI
48	Chloroplast pigment-binding protein CP26	*P. vulgaris*		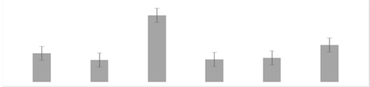	*	NS	NS	CHL	PS
49	Chaperonin 21	*C. cardunculus*		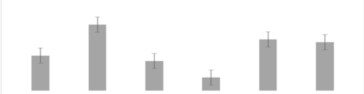	*	NS	*	CHL	PM
54	Ascorbate peroxidase	*P. brachycarpa*		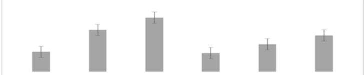	*	*	NS	CYT	RX
55	Cytosolic ascorbate peroxidase	*V. vinifera*		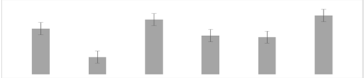	*	NS	NS	CYT	RX
56	20 kDa Chaperonin family protein	*P. trichocarpa*		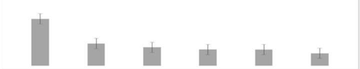	*	*	*	MC	PM
57	Oxygen-evolving enhancer protein 1	*S. lycopersicum*		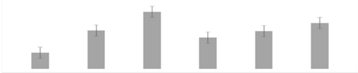	*	NS	NS	CHL	PS
58	Oxygen-evolving enhancer protein 1	*S. lycopersicum*		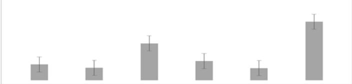	*	NS	NS	CHL	PS
59	Cytosolic ascorbate peroxidase	*V. vinifera*	99	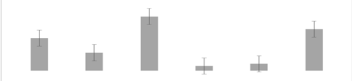	*	*	NS	CYT	RX
60	20S Proteasome α subunit G1	*T. cacao*	96	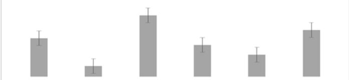	*	NS	NS	CYT/ NUC	PM
61	20 kDa Chaperonin family protein	*P. trichocarpa*		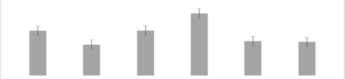	*	NS	NS	MC	PM
62	Carbonic anhydrase 2-like isoform X2	*E. grandis*		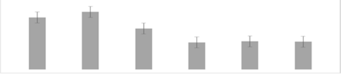	NS	*	NS	CHL	TCA

65	Glyceraldehyde-3-phosphate dehydrogenase A	*C. sinensis*	99	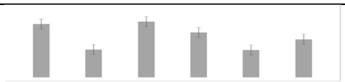	*	NS	NS	CHL	PS
66	Glyceraldehyde-3-phosphate dehydrogenase A subunit 1	*B. oleracea var. oleracea*	98	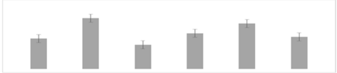	*	NS	NS	CHL	PS
67	Malate dehydrogenase	*M. sativa*		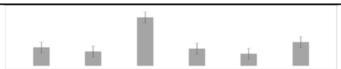	*	NS	NS	CYT	TCA
68	Malate dehydrogenase	*C. arietinum*		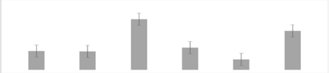	*	NS	NS	CYT	TCA
69	Ferredoxin--NADP reductase, leaf isozyme	*Z. mays*	87	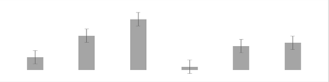	*	*	NS	CHL	PS
70	Ferredoxin--NADP reductase, leaf isozyme 1	*T. hassleriana*	90	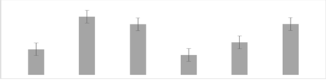	*	NS	NS	CHL	PS
71	Isoflavone reductase-like NAD(P)H-dependent oxidoreductase	*P. vulgaris*		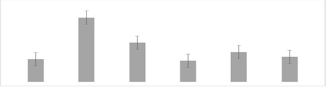	*	*	NS	CYT	SM
72	Guanine nucleotide-binding protein subunit β-like protein	*P. euphratica*	99	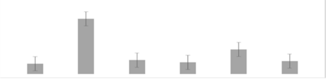	*	*	*	CYT	DE
73	Glyceraldehyde-3-phosphate dehydrogenase A	*C. sinensis*	99	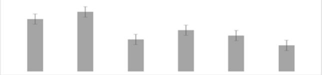	*	*	NS	CHL	PS
74	Glyceraldehyde-3-phosphate dehydrogenase C subunit1	*A. thaliana*	96	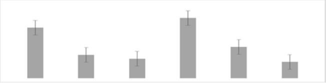	*	NS	NS	CYT	GS
75	Glyceraldehyde-3-phosphate dehydrogenase B	*N. tabacum*		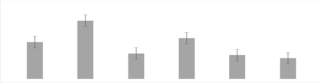	*	*	*	CHL	PS
76	Glyceraldehyde-3-phosphate dehydrogenase A	*E. grandis*		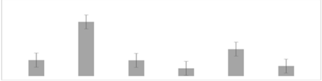	*	*	*	CHL	PS
77	Glyceraldehyde-3-phosphate	*T. hassleriana*		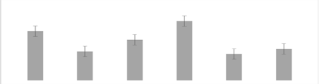	*	NS	NS	CYT	GS

	dehydrogenase C subunit1								
78	Glyceraldehyde-3-phosphate dehydrogenase C subunit1	*C. sinensis*	99	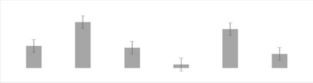	*	NS	NS	CYT	GS
79	26S Proteasome AAA-ATPase subunit family protein	*P. trichocarpa*		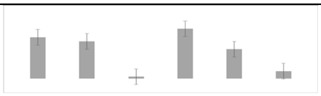	*	NS	*	CYT/ NUC	PM
80	Glycerate dehydrogenase	*R. communis*	95	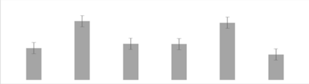	*	NS	NS	CHL	AAM
81	Glyceraldehyde-3-phosphate dehydrogenase C subunit1	*A. thaliana*		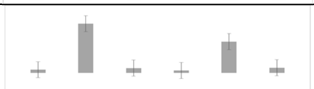	*	NS	NS	CYT	GS
82	Serine--glyoxylate aminotransferase	*C. sinensis*	99	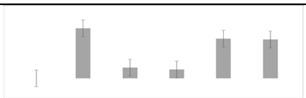	*	NS	*	PER	AAM
86	Actin 11, partial	*L. cubeba*		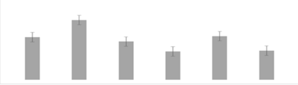	*	*	NS	CYT	CE

[a] Position numbers shown correspond to the specific numbers on the six representative gels in **Figure S2**. [b] The percentage of sequences similarity of BlastP search, unless it is identical protein by LC-MS/MS. [c] The average value of relative transformed abundance of each spot across four N treatments consist of three genotypes. Error bars indicate standard error of mean. [d] The N/genotype effects and interaction assigned on the **Table S1**, while asterisk (*) indicates $p < 0.05$ and "NS" indicates not significance. [e] Known/ predicated cellular locations based on the SUBA database. CHL, chloroplast; CYT, cytosol; MC, mitochondrion; PER, peroxisome; GA, glogi apparatus; NUC, nucleus. [e] The nonredundant functional classes were generated by using MapMan bin codes. PS, photosynthesis; PM, protein metabolism; GS, glycolysis; RX, redox; AAM, amino acid metabolism; TCA, tricarboxylic acid cycle; OPP, oxidative pentose phosphate; TR, transport; SI, signaling; CE, cell; SM, secondary metabolism; ST, stress; BX, biodegradation of Xenobiotics; DE, development; NA, not assigned; NM, N-metabolism; MCM, minor CHO metabolism.

Table S5: Identification of significantly altered proteins after 112 kg N/ha treatment by LC-MS/MS

Pos. NO. [a]	Protein Name	*Organism*	Sequence Similarity (%) [b]	Expression Profile [c]	Two-way ANOVA [d]			Cellular location [e]	Functional Class
				A0 B0 W0 A112 B112 W112	Genotype	Nitrogen	Interaction		
1	Elongation factor 2-like	*C. sinensis*	99	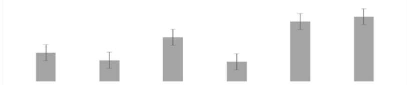	*	NS	NS	CYT	PM
2	Transketolase family protein	*P. trichocarpa*		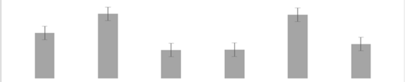	*	NS	NS	CHL	OPP
3	Transketolase	*A. trichopoda*	100	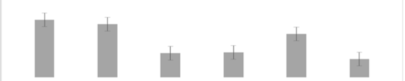	*	*	NS	CHL	OPP
5	Heat shock protein 70	*L. sativa*		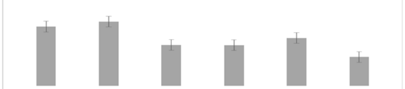	*	*	NS	CHL	ST
7	Phosphoglucomutase	*P. tremula*		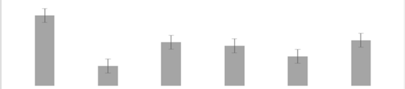	*	NS	NS	CYT	GS
10	Chaperonin 60 subunit β 1	*C. sativa*	97	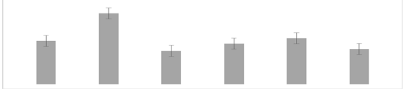	*	NS	NS	CHL	PM
13	ATP synthase CF1 α subunit	*C. maculata*		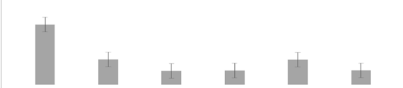	*	*	*	CHL	PS
14	ATP synthase CF1 α subunit	*I. cornuta*		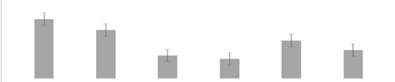	NS	*	*	CHL	PS
17	Mitochondrial lipoamide dehydrogenase 1	*T. cacao*		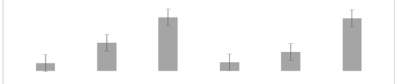	*	NS	NS	MC	TCA
18	Catalase, partial	*B. juncea*		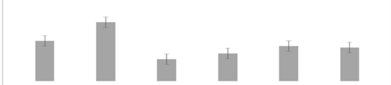	*	NS	*	PER	RX
20	RubisCO activase α form precursor	*D. antarctica*		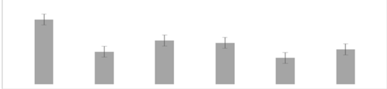	*	*	NS	CHL	PS

21	30S Ribosomal protein S1	*A. trichopoda*	100	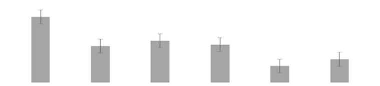	*	*	NS	CHL	PM
22	Eukaryotic initiation factor 4A-8	*Z. jujuba*	98	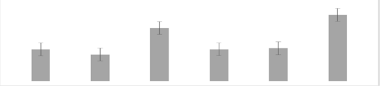	*	NS	NS	CYT	PM
23	Citrate synthase family protein	*P. trichocarpa*		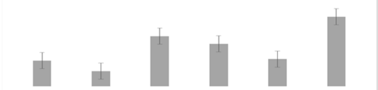	*	NS	NS	MC	TCA
24	S-adenosylmethionine synthase 1	*O. sativa subsp. japonica*		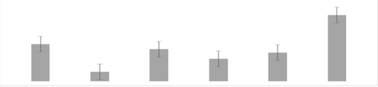	*	*	*	CYT	AAM
25	GDP-mannose 3,5-epimerase isoform X2	*C. sativa*	98	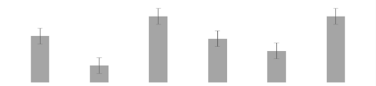	*	NS	NS	GA	RX
26	Glyceraldehyde-3-phosphate dehydrogenase B	*N. tabacum*		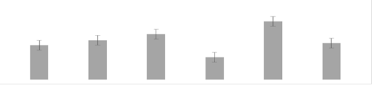	*	NS	*	CHL	PS
27	Phosphoglycerate kinase	*Z. mays*		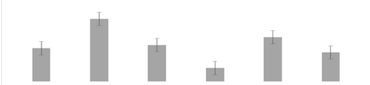	*	*	NS	CHL	PS
29	Monodehydroascorbate reductase family protein	*P. trichocarpa*		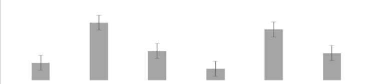	*	NS	NS	CYT	RX
30	Phosphoglycerate kinase	*N. tabacum*		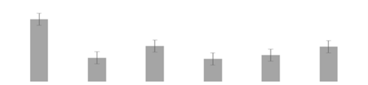	*	*	*	CHL	PS
31	3-phosphoglycerate kinase, partial	*C. nutans*		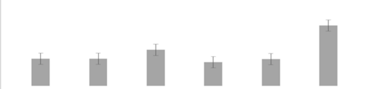	*	NS	NS	CHL	PS
32	Elongation factor, GTP-binding domain-containing protein	*C. cardunculus*		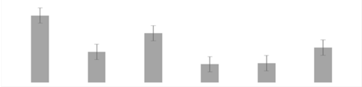	*	*	*	MC	PM
33	Glutamine synthetase nodule isozyme	*T. hassleriana*		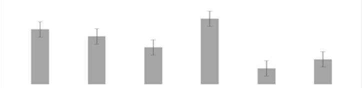	*	NS	NS	CYT	NM
35	Chloroplast RubisCO activase large protein isoform	*A. rubrum*	91	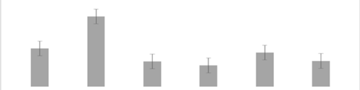	*	*	*	CHL	PS

36	Putative lactoylglutathione lyase	*C. sinensis*	98	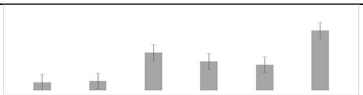	*	*	NS	PER	BX
40	Photosystem II stability/assembly factor HCF136	*O. sativa subsp. japonica*		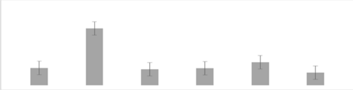	*	*	*	CHL	PM
42	Monomeric lectin SNAlm precursor	*S. nigra*		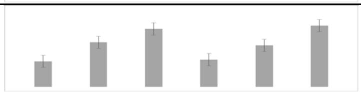	*	NS	NS	Unkown	NA
43	4-Nitrophenylphosphatase	*Z. mays*		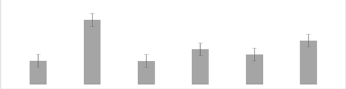	NS	NS	*	CHL	MCM
44	Oxygen-evolving enhancer protein 1	*N. tabacum*		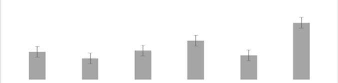	*	*	NS	CHL	PS
46	Chlorophyll a-b binding protein of LHCII type 1-like	*V. radiata*		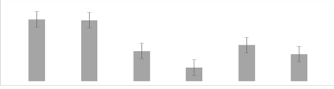	*	*	*	CHL	PS
50	2-Cys peroxiredoxin BAS1	*A. thaliana*		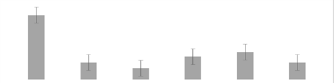	*	NS	*	CHL	RX
51	Ascorbate peroxidase	*P. brachycarpa*		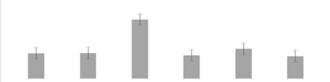	NS	NS	*	CYT	RX
52	Chloroplast pigment-binding protein CP26	*P. vulgaris*		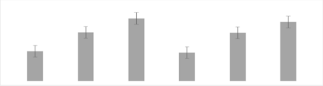	*	NS	NS	CHL	PS
53	Probable plastid-lipid-associated protein 6	*C. arietinum*		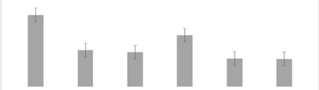	*	NS	NS	CHL	CE
54	Ascorbate peroxidase	*P. brachycarpa*		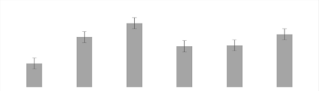	*	NS	NS	CYT	RX
55	Cytosolic ascorbate peroxidase	*V. vinifera*		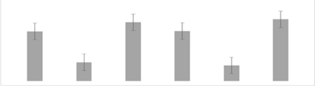	*	NS	NS	CYT	RX
56	20 kDa Chaperonin family protein	*P. trichocarpa*		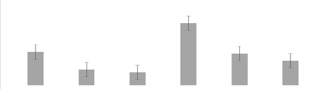	*	*	NS	MC	PM

57	Oxygen-evolving enhancer protein 1	*S. lycopersicum*		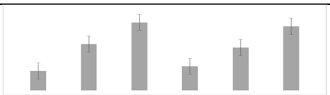	*	NS	NS	CHL	PS
63	Outer plastidial membrane protein porin-like	*C. arietinum*		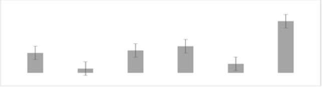	*	*	NS	MC	TR
69	Ferredoxin--NADP reductase, leaf isozyme	*Z. mays*	87	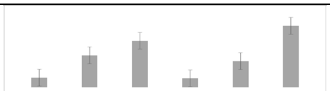	*	NS	NS	CHL	PS
72	Guanine nucleotide-binding protein subunit β-like protein	*P. euphratica*	99	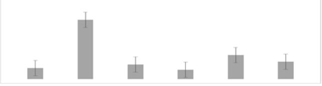	*	*	*	CYT	DE
76	Glyceraldehyde-3-phosphate dehydrogenase A	*E. grandis*		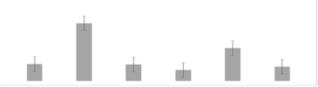	*	*	*	CHL	PS
77	Glyceraldehyde-3-phosphate dehydrogenase C subunit1	*T. hassleriana*		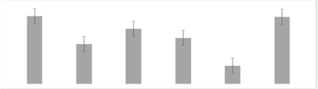	*	NS	NS	CYT	GS
78	Glyceraldehyde-3-phosphate dehydrogenase C subunit1	*C. sinensis*	99	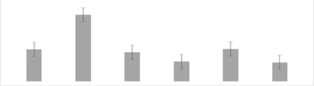	*	*	NS	CYT	GS
79	26S Proteasome AAA-ATPase subunit family protein	*P. trichocarpa*		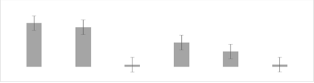	*	*	NS	CYT/ NUC	PM
81	Glyceraldehyde-3-phosphate dehydrogenase C subunit1	*A. thaliana*		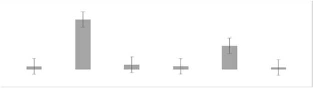	*	*	*	CYT	GS
82	Serine--glyoxylate aminotransferase	*C. sinensis*	99	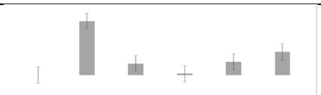	*	*	*	PER	AAM
83	3-Ketoacyl-CoA thiolase 2	*A. thaliana*		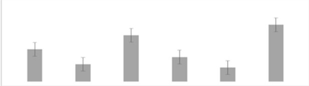	*	NS	NS	PER	AAM
84	Glycerate dehydrogenase	*R. communis*	95	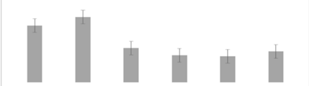	NS	*	*	CHL	AAM
85	Outer plastidial membrane protein porin-like	*C. arietinum*		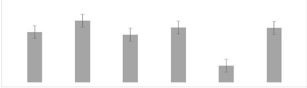	NS	NS	*	MC	TR

87	Aminomethyltransferase	*M. crystallinum*		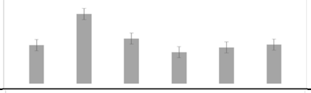	NS	*	NS	MC	AAM
88	Triosephosphate isomerase	*C. sinensis*	97	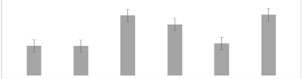	*	NS	NS	CYT	GS
89	Glyceraldehyde-3-phosphate dehydrogenase C subunit1	*A. thaliana*		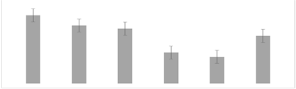	NS	*	NS	CYT	GS

[a] Position numbers shown correspond to the specific numbers on the six representative gels in **Figure S2**. [b] The percentage of sequences similarity of BlastP search, unless it is identical protein by LC-MS/MS. [c] The average value of relative transformed abundance of each spot across four N treatments consist of three genotypes. Error bars indicate standard error of mean. [d] The N/genotype effects and interaction assigned on the **Table S1**, while asterisk (*) indicates $p < 0.05$ and “NS” indicates not significance. [e] Known/ predicated cellular locations based on the SUBA database. CHL, chloroplast; CYT, cytosol; MC, mitochondrion; PER, peroxisome; GA, glogi apparatus; NUC, nucleus. [e] The nonredundant functional classes were generated by using MapMan bin codes. PS, photosynthesis; PM, protein metabolism; GS, glycolysis; RX, redox; AAM, amino acid metabolism; TCA, tricarboxylic acid cycle; OPP, oxidative pentose phosphate; TR, transport; SI, signaling; CE, cell; SM, secondary metabolism; ST, stress; BX, biodegradation of Xenobiotics; DE, development; NA, not assigned; NM, N-metabolism; MCM, minor CHO metabolism.

Table S6: Identification of significantly altered proteins after 169 kg N/ha treatment by LC-MS/MS

Pos. No. [a]	Protein Name	*Organism*	Sequence Similarity (%) [b]	Expression Profile [c]	Two-way ANOVA [d]			Cellular location [e]	Functional Class
				A0 B0 W0 A169 B169 W169	Genotype	Nitrogen	Interaction		
2	Transketolase family protein	*P. trichocarpa*		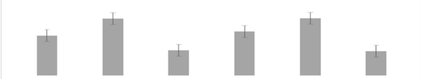	*	NS	NS	CHL	OPP
3	Transketolase	*A. trichopoda*	100	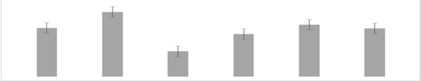	*	NS	*	CHL	OPP
6	Phosphoglucomutase	*V. vinifera*		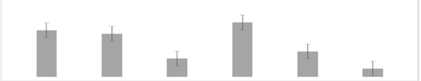	*	NS	NS	CYT	GS
10	Chaperonin 60 subunit β 1	*C. sativa*	97	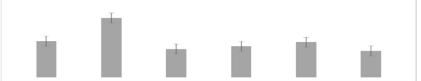	*	*	NS	CHL	PM
12	V-type proton ATPase catalytic subunit A	*B. rapa*	98	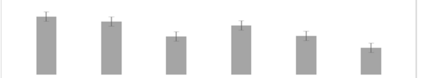	*	*	NS	MC	TR
16	Ketol-acid reductoisomerase	*P. euphratica*	99	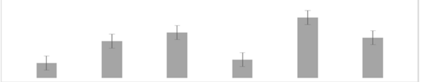	*	NS	*	CHL	AAM
17	Mitochondrial lipoamide dehydrogenase 1	*T. cacao*		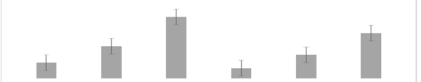	*	NS	NS	MC	TCA
18	Catalase, partial	*B. juncea*		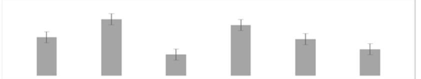	*	NS	*	PER	RX
20	RubisCO activase α form precursor	*D. antarctica*		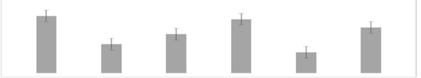	*	NS	NS	CHL	PS
28	Glyceraldehyde-3-phosphate dehydrogenase B	*N. tabacum*		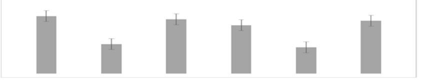	*	NS	NS	CHL	PS
29	Monodehydroascorbate reductase family protein	*P. trichocarpa*		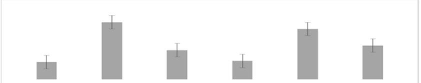	*	NS	NS	CYT	RX
30	Phosphoglycerate kinase	*N. tabacum*		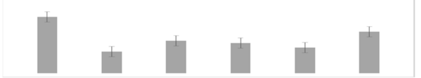	*	NS	*	CHL	PS

32	Elongation factor, GTP-binding domain-containing protein	*C. cardunculus*		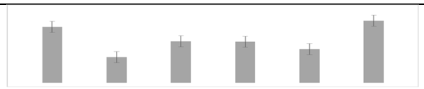	*	NS	*	MC	PM
34	Glutamine synthetase cytosolic isozyme 1-1-like	*B. napus*	96	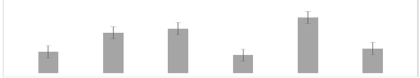	*	NS	*	CYT	NM
36	Putative lactoylglutathione lyase	*C. sinensis*	98	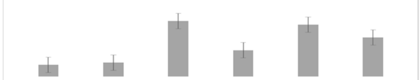	*	*	*	PER	BX
40	Photosystem II stability/assembly factor HCF136	*O. sativa subsp. japonica*		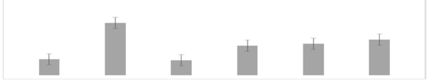	*	NS	*	CHL	PM
41	Oxygen-evolving enhancer protein 1	*S. lycopersicum*		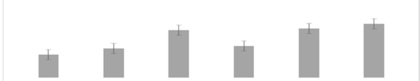	*	*	NS	CHL	PS
42	Monomeric lectin SNAlm precursor	*S. nigra*		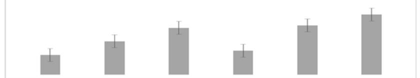	*	*	NS	Unkown	NA
43	4-Nitrophenylphosphatase	*Z. mays*		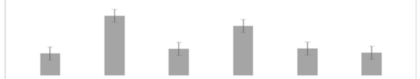	*	NS	*	CHL	MCM
44	Oxygen-evolving enhancer protein 1	*N. tabacum*		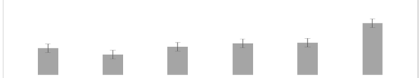	NS	*	NS	CHL	PS
45	Serine/threonine-protein phosphatase PP2A-4 catalytic subunit	*P. euphratica*	98	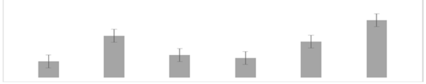	*	NS	*	CYT	PM
46	Chlorophyll a-b binding protein of LHCII type 1-like	*V. radiata*		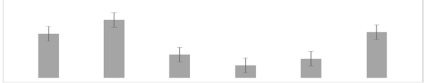	NS	*	*	CHL	PS
48	Chloroplast pigment-binding protein CP26	*P. vulgaris*		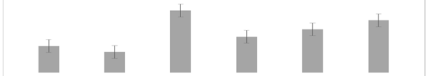	*	NS	NS	CHL	PS
49	Chaperonin 21	*C. cardunculus*		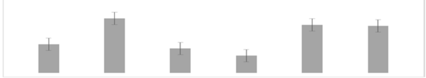	*	NS	*	CHL	PM
51	Ascorbate peroxidase	*P. brachycarpa*		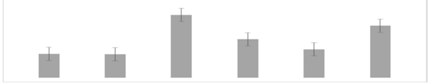	*	NS	NS	CYT	RX

55	Cytosolic ascorbate peroxidase	*V. vinifera*		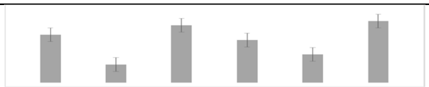	*	NS	NS	CYT	RX
64	Ferredoxin--NADP reductase, leaf isozyme	*Z. mays*	87	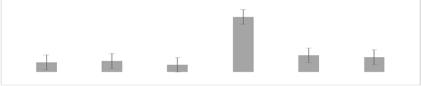	*	*	*	CHL	PS
65	Glyceraldehyde-3-phosphate dehydrogenase A	*C. sinensis*	99	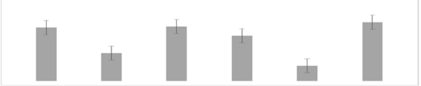	*	NS	NS	CHL	PS
66	Glyceraldehyde-3-phosphate dehydrogenase A subunit 1	*B. oleracea var. oleracea*	98	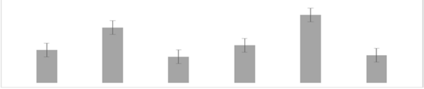	*	NS	NS	CHL	PS
67	Malate dehydrogenase	*M. sativa*		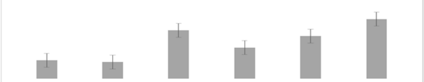	*	*	NS	CYT	TCA
69	Ferredoxin--NADP reductase, leaf isozyme	*Z. mays*	87	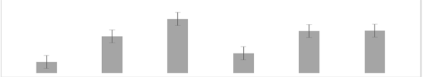	*	NS	NS	CHL	PS
71	Isoflavone reductase-like NAD(P)H-dependent oxidoreductase	*P. vulgaris*		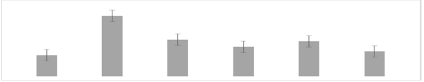	*	NS	*	CYT	SM
72	Guanine nucleotide-binding protein subunit β-like protein	*P. euphratica*	99	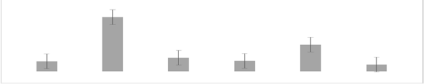	*	*	*	CYT	DE
73	Glyceraldehyde-3-phosphate dehydrogenase A	*C. sinensis*	99	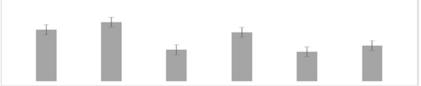	NS	NS	*	CHL	PS
74	Glyceraldehyde-3-phosphate dehydrogenase C subunit1	*A. thaliana*	96	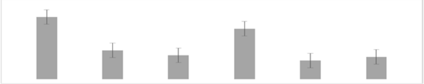	*	NS	NS	CYT	GS
75	Glyceraldehyde-3-phosphate dehydrogenase B	*N. tabacum*		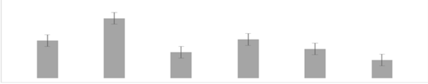	*	*	*	CHL	PS
76	Glyceraldehyde-3-phosphate dehydrogenase A	*E. grandis*		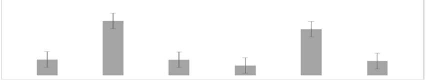	*	*	NS	CHL	PS
77	Glyceraldehyde-3-phosphate	*T. hassleriana*		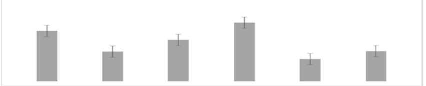	*	NS	NS	CYT	GS

	dehydrogenase C subunit1								
78	Glyceraldehyde-3-phosphate dehydrogenase C subunit1	*C. sinensis*	99	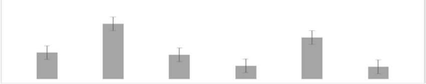	*	*	NS	CYT	GS
90	20 kDa Chaperonin	*S. italica*	92	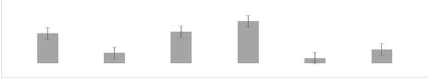	*	NS	NS	MC	PM
91	Ferredoxin--NADP reductase, leaf isozyme 1	*T. hassleriana*	90	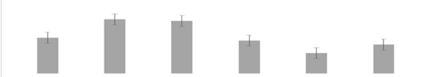	NS	*	NS	CHL	PS
92	Ferredoxin--NADP reductase, leaf isozyme	*Z. mays*	87	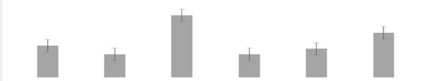	*	NS	NS	CHL	PS
93	Aspartate aminotransferase, chloroplastic isoform X1	*C. sinensis*	98	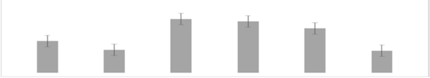	NS	NS	*	CHL	AAM
94	Isocitrate dehydrogenase [NADP]	*C. sinensis*	99	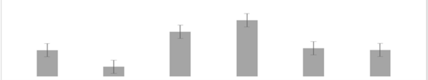	NS	NS	*	PER	TCA
95	Cysteine synthase	*T. cacao*	93	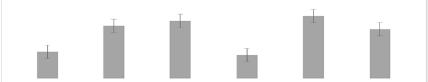	*	NS	NS	CYT	AAM
96	Triosephosphate isomerase	*V. vinifera*		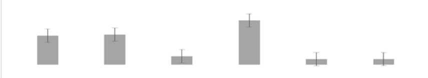	*	NS	NS	CYT	GS
97	Sedoheptulose-1,7-bisphosphatase	*T. aestivum*		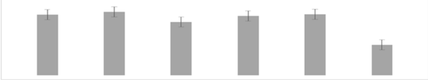	*	NS	NS	CHL	PS
98	20S Proteasome subunit PBA1	*A. thaliana*	99	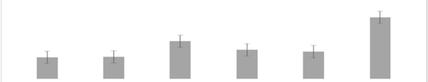	*	*	NS	CYT	PM
99	Probable ribose-5-phosphate isomerase-like	*C. sativus*		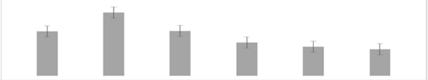	NS	*	NS	CYT	OPP
100	GTP-binding nuclear protein Ran-2	*A. thaliana*	98	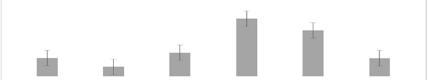	NS	*	NS	CYT	SI
101	Glyceraldehyde-3-phosphate dehydrogenase A subunit 1	*B. oleracea var. oleracea*	98	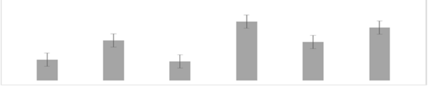	NS	*	*	CHL	PS

[a] Position numbers shown correspond to the specific numbers on the six representative gels in **Figure S2**. [b] The percentage of sequences similarity of BlastP search, unless it is identical protein by LC-MS/MS. [c] The average value of relative transformed abundance of each spot across four N treatments consist of three genotypes. Error bars indicate standard error of mean. [d] The N/genotype effects and interaction assigned on the **Table S1**, while asterisk (*) indicates $p < 0.05$ and "NS" indicates not significance. [e] Known/ predicated cellular locations based on the SUBA database. CHL, chloroplast; CYT, cytosol; MC, mitochondrion; PER, peroxisome; GA, glogi apparatus; NUC, nucleus. [e] The nonredundant functional classes were generated by using MapMan bin codes. PS, photosynthesis; PM, protein metabolism; GS, glycolysis; RX, redox; AAM, amino acid metabolism; TCA, tricarboxylic acid cycle; OPP, oxidative pentose phosphate; TR, transport; SI, signaling; CE, cell; SM, secondary metabolism; ST, stress; BX, biodegradation of Xenobiotics; DE, development; NA, not assigned; NM, N-metabolism; MCM, minor CHO metabolism.
